# Wetting Behaviour of Water, Ethanol, Rhamnolipid, and Triton X-165 Mixture in the Polymer–Solution Drop–Air System

**DOI:** 10.3390/molecules28155858

**Published:** 2023-08-03

**Authors:** Anna Zdziennicka, Edyta Rekiel, Katarzyna Szymczyk, Wojciech Zdziennicki, Bronisław Jańczuk

**Affiliations:** 1 Department of Interfacial Phenomena, Institute of Chemical Sciences, Faculty of Chemistry, Maria Curie-Skłodowska University in Lublin, Maria Curie-Skłodowska Sq. 3, 20-031 Lublin, Poland; rekieel@gmail.com (E.R.); katarzyna.szymczyk@mail.umcs.pl (K.S.); bronislaw.janczuk@mail.umcs.pl (B.J.); 2 University Clinical Hospital in Poznań, Przybyszewskiego 49, 60-355 Poznań, Poland; wojtekzdziennicki@gmail.com

**Keywords:** wettability, contact angle, adsorption, Gibbs free energy of adsorption, rhamnolipid, ethanol, Triton X-165

## Abstract

Despite the fact that the wetting properties of multicomponent mixtures including the surface active compounds play a very important role in many practical applications, they are not sufficiently known. Thus, the wettability of polytetrafluoroethylene (PTFE) and poly (methyl methacrylate) (PMMA) by the water + ethanol (ET) solution of rhamnolipid (RL) with Triton X-165 (TX165) mixture was studied. The investigations involved measuring the advancing contact angles of this solution on PTFE and PMMA by varying the concentration of TX165 while maintaining a constant concentration of ET and RL. Additionally, a thermodynamic analysis was conducted to obtain the compositions and concentrations of the ET, RL, and TX165 mixtures at the different interfaces. The composition and concentration of the interface mixed layer were considered using two different approaches to the wetting process. From these considerations, it follows that, depending on the ET concentration, it is possible to form the TX165 + RL layer at the solid–water + ET mixed solvent, as well as the water + ET–air interfaces, but not at the solid–water and water–air ones. This conclusion is in accordance with the Gibbs standard free energy of adsorption of particular components of the studied mixture at the solution–air and solid–solution interfaces.

## 1. Introduction

It is hard to find an area of industry or household activities where surfactants are not used. In many practical applications, the wetting properties of surfactants or their mixtures play an essential role [1,2,3,4,5]. More than 200 years have passed since Thomas Young considered the state of equilibrium in the solid–liquid drop–air system [6]. In turn, this state was mathematically described by Dupre and is commonly called Young’s equation [7]. Until now, this equation has been used to characterise the wetting properties of a liquid or solution in relation to a given solid [1,7,8,9]. The main problem in the application of Young’s equation for the analysis of the given solid’s wettability by various liquids or solutions is two thermodynamic quantities that are difficult to measure directly, namely the solid surface tension and the solid–liquid interface tension. 

To solve this problem, a number of theories have been developed regarding the relationship between the solid–liquid interface tension and the surface tension of liquids and solids [4,10,11,12,13,14]. Two fundamentally different approaches can be distinguished among them. One states that the solid–liquid interface tension is a function of the components and parameters of the surface tension of the solid and liquid, resulting from different intermolecular interactions [4,10,11,12,14]. The other states that the solid–liquid interface tension is a function of only the total values of the solid and liquid surface tension [13]. It should be mentioned that all the approaches to the solid–liquid interface tension were based on the contact angle of individual liquids on the given solid on the assumption that, for a contact angle higher than zero, the solid surface tension does not depend on the kind of liquid [4,10,11,12,13,14]. In other words, a liquid with a higher surface tension than a solid does not reduce its surface tension [4,12,15,16]. 

This is more complicated especially in the case of aqueous surfactant solutions. In such a case, the surface tension of the surfactant may be lower than that of the given solid, even though the surface tension of the solution is higher. It should be also taken into account that the surface tension of surfactants depends on the orientation of their molecules towards the air [1,17]. In the case of aqueous solutions of surfactants, the value of the contact angle is significantly influenced by their adsorption layers at the solution–air, solid–solution, and solid–air interfaces [1,2,3]. This influence is more complicated in the case of a mixture of surfactants or surfactants with various additives because, in addition to the orientation and packing of surfactant molecules in the adsorption layers, the composition of these layers has a great impact. Combining the properties of the mixed adsorption layers at different interfaces with the contact angle on a given solid can not only have theoretical, but also practical significance.

The literature reports few studies on this problem in the case of mixtures of surfactants with biosurfactants as additives. Among the biosurfactants, rhamnolipid (RL) is of great interest due to its high surface activity and low CMC, as well as its antibacterial and antiviral properties [18,19,20,21,22]. However, its practical application is limited, among others, due to the high cost of obtaining it. It is more realistic to use it as an addition to classic surfactants. Therefore, in our research, monorhamnolipid was used as an additive to non-ionic Triton X-165 (TX165). The RL and TX165 were dissolved in the mixed water–ethanol solvent at a constant ethanol (ET) concentration. In such a way, 16 series of solutions were obtained in which the ET and RL concentration was constant and the TX165 variable in the range of 0 to 4 × 10^−3^ mol/dm^3^ for each series. For such solutions, the contact angle was measured on the surfaces of PTFE and PMMA. The PTFE (PMMA)–solution drop–air system can be treated as a model one for the studies of the relationship between the contact angle and the properties of the mixed monolayers at three interfaces taking into account the physicochemical properties of individual components of the solution mixture and solid, as well as their practical application. It should be mentioned that both PTFE and PMMA are, among others, applied in medicine as implants, as well as a model of human skin in the study of its wettability [23,24,25].

The aim of our studies on such chosen systems was to establish the correlation between the contact angle, composition, and concentration of particular components of the mixture at the polymer–air, polymer–solution, and solution–air interfaces, as well the Gibbs free energy of adsorption. For this aim, the concepts of van Oss et al. [4,17,26,27], Neumann et al. [13,28], as well as the Frumkin equation were used [1,7]. The thermodynamic considerations were based on the interface area occupied by the particular mixture components and the proper model of the interface region.

Based on the concepts of van Oss et al. Neumann et al., and the Frumkin equation, it was expected to be able to explain whether the wettability of solids depends on the solid–air, solid–solution, and solution–air interface tensions or on the components of these tensions resulting from various types of intermolecular interactions. It was also expected to be able to explain the mechanism of the mixed surface layer formation at these interfaces. The mechanism of the mixed layer formation at the interfaces including polymers is not only important from the theoretical point of view, but also from the practical application point of view, for example in medicine to protect polymeric implants against bacteria.

## 2. Results and Discussion

### 2.1. Wetting and Adsorption Properties of ET, RL, and TX165

In order to understand the mutual influence of ET, RL, and TX165 on the wetting properties in the systems involving PTFE or PMMA, it is necessary to understand their individual wetting properties. PTFE and PMMA, which are widely used in practice, are excellent models for testing the wetting properties of various types of surfactants by measuring the contact angle in the polymer–solution drop–air system.

According to van Oss et al. [4,17,26,27], PTFE is an apolar and PMMA a monopolar solid. The surface tension of these solids results from only the Lifshitz–van der Waals (LW) intermolecular interactions, but PMMA can interact through the Lewis acid–base (AB) interactions with the adherent medium. Thus, the contact angle in the PTFE–solution drop–air system depends on only the LW component of PTFE and the surface tension of the aqueous solution of surfactants and their mixtures. On the other hand, the contact angle in the PMMA–solution drop–air system depends, apart from the LW component, on the electron donor parameter ($\gamma^{-}$) of the PMMA surface tension and the electron acceptor parameter ($\gamma^{+}$) of the solution.

The surface tension of the aqueous solutions of ET, RL, and TX165 depends on the surface tension components of the water and surfactants. However, in the case of the surfactants, their surface tension depends on the molecules’ orientation toward the water phase and is called the tail and head surface tension [1,17]. The LW components of the ET, RL, and TX165 tails is nearly the same and close to that of water determined from the *n*-alkane–water interface tension [2,12,29]. However, in the case of water, the LW component of its surface tension determined based on the contact angle is higher than that obtained from the interface tension [30,31]. Nevertheless, based on the LW component of the surface tension of the ET, TX165, and RL tails and water, it can be concluded that changes in the solution surface tension ($\gamma_{\mathrm{LV}})$ as a function of surfactant concentration, and thus the contact angle, result mainly from the reduction of the AB component of the solution surface tension.

**Figure 1:**
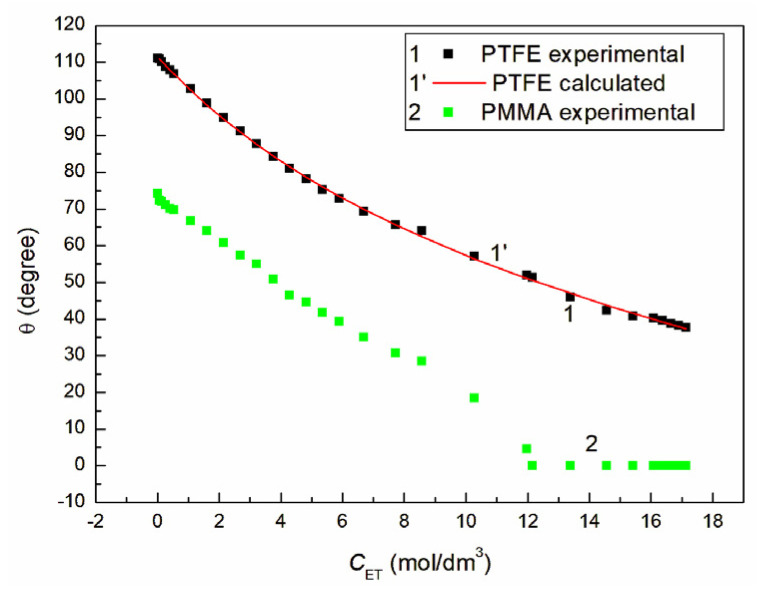
A plot of the contact angle (θ) of the ET aqueous solutions vs. its concentration ($C_{\mathrm{ET}}$ ). Points 1 and 2 correspond to the values measured on PTFE and PMMA, respectively. Curve 1′ corresponds to the values calculated for PTFE from the second-order exponential function.

**Figure 2:**
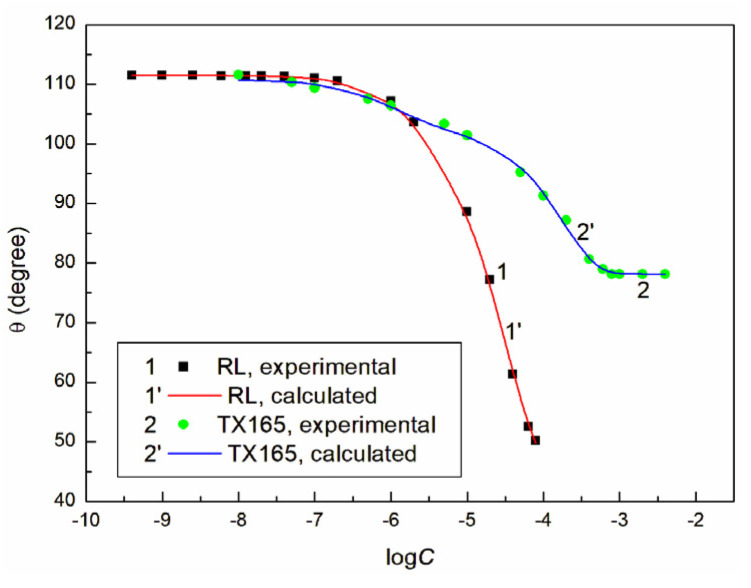
A plot of the contact angle (θ) of the aqueous solutions of RL (Point 1 and Curve 1′) and TX165 (Point 2 and Curve 2′) on PTFE vs. the logarithm of their concentration (logC ). Points 1 and 2 correspond to the measured values. Curves 1′ and 2′ correspond to the values calculated from the second-order exponential function.

The analysis of the surface tension ($\gamma_{\mathrm{LV}}$) of the aqueous solutions of ET, RL, and TX165 showed that the surface tension changes as a function of these compounds’ concentration can be described by the second-order exponential function. This analysis also proved that the constants of the second-order exponential function are related to the components and parameters of the solution surface tension. It is possible that such a relationship takes place in the case of the contact angle (θ), whose isotherms can be also described by the second-order exponential function (Figure 1, Figure 2 and Figure S1).

However, in the case of the contact angle of the aqueous solution of ET and RL on the PMMA surface, it was impossible to describe its isotherm by the exponential function. The values of the LW of ET, the tails of RL and TX165, as well as the constant in the exponential- function- described isotherm of the contact angle indicate clearly that complete spreading of the PTFE surface by the aqueous solutions of ET, RL, and TX165 is impossible. This is confirmed by the data in Figure 1, Figure 2 and Figure S1. However, in the case of PMMA, contrary to PTFE, the LW component of ET, the tails of RL and TX165, as well as the total surface tension of the heads of RL and TX165 are smaller than the PMMA surface tension ($\gamma_{\mathrm{SV}})$ [2,30], but only for the aqueous solution of ET having a surface tension much smaller than that of PMMA, its complete spreading on this polymer is observed (Figure 1). Similar to the aqueous solution of ET, the minimal surface tension of the RL and TX165 solutions is lower than that of PMMA [2,30] (Table 1). Despite this, there is no complete spreading of the aqueous solutions on the PMMA surface, contrary to the opinion of some authors [2,3,4,13].

To explain this problem, the Young–Dupre equation, as well as the Neumann et al. [13,28] and van Oss et al. concepts can be applied to the wetting process [4,17,26,27]. According to the Young–Dupre equation, the cosine of the contact angle (*θ*) fulfils the relationship [1,7]:

(1) $$\cos\theta =\frac{\gamma_{\mathrm{SV}}}{\gamma_{\mathrm{LV}}}-\frac{\gamma_{\mathrm{SL}}}{\gamma_{\mathrm{LV}}},$$

where $\gamma_{\mathrm{SL}}$ is the solid–liquid (solution) interface tension.

**Figure 3:**
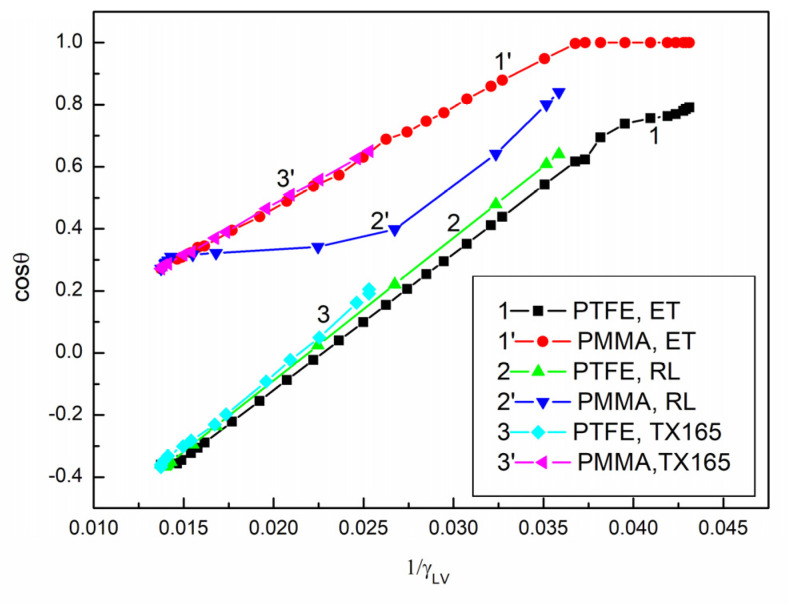
A plot of the cosine of the contact angle (cosθ) for ET (Curves 1 and 1′), RL (Curves 2 and 2′), and TX165 (Curves 3 and 3′) vs. the reciprocal of the surface tension ($\frac{1}{\gamma_{\mathrm{LV}}}$ ). Curves 1–3 correspond to the values measured on PTFE. Curves 1′–3′ correspond to the values measured on PMMA.

It follows from Equation (1) that cosθ, among others, is the function of $\frac{1}{\gamma_{\mathrm{LV}}}$. It appears that, in the case of PTFE, there is a linear relationship between cosθ and $\frac{1}{\gamma_{\mathrm{LV}}}$ ($\cos\theta =m\frac{1}{\gamma_{\mathrm{LV}}}+k$) for the ET, RL, and TX165 solutions and a nonlinear one for the RL solution in the case of PMMA (Figure 3, Curve 2′).

The constant *k* in this linear function for PTFE is close to −1 for all solutions, but the constant *m* is slightly different for the solutions of ET, RL, and TX165. The constant 𝑘 for PMMA is greater than for PTFE and the constant *m* smaller than for PTFE (Table 2). What information is proven by the relationship between the cosine of the contact angle and the reciprocal of the surface tension? The answer can be obtained based on the Young–Dupre equation for the work of adhesion of a liquid to a solid ($W_{a})$ and the van Oss et al. [4,17,26,27] and the Neumann et al. [13,28] equations. From the Young–Dupre equation, it results that:

(2) $$\cos\theta =-1+\frac{W_{a}}{\gamma_{\mathrm{LV}}},$$

From Equation (2) and $\cos\theta =m\frac{1}{\gamma_{\mathrm{LV}}}+k$, it results that, if the constant *k* is close to −1, then the constant *m* is equal to the adhesion work of the solution to the PTFE surface. This also indicates that the adhesion work of the aqueous solution of ET, RL, and TX165 to the PTFE surface does not depend on the type of surface active compound and its concentration. However, it takes place for ET not in its whole concentration range, but only from 0.067 to 14.57 mol/dm^3^. This means that, at the ET concentration higher than 14.57 M, the reduction of the LW component of water approaches the ET surface tension. In the case of PMMA, there is no correlation between the constant *k,* in the linear dependence between cosθ and $\frac{1}{\gamma_{\mathrm{LV}}}$, and the adhesion work of the ET and RL solution to the PMMA surface. Moreover, for the aqueous solution of RL, there is no linear dependence between cosθ and $\frac{1}{\gamma_{\mathrm{LV}}}$, as mentioned above. Thus, based on Equation (2), it is impossible to explain the wetting behaviour of the aqueous solution of ET, RL, and TX165 in the system including PMMA. It is also not possible to give an answer based on the Young–Dupre equation why, despite the high adsorption activity of RL and TX165 at the water–air interface, they show poor wetting properties in relation to PTFE and PMMA, as well as because the contact angle of ET on PTFE has higher values than excepted taking into account the small difference between the surface tension of PTFE and ET (Figure 1) [2,3].

van Oss et al. [4,17,26,27] proposed that, for the solid–liquid drop–air system, when the contact angle is higher than zero, the following relationship should be fulfilled:

(3) $$\gamma_{\mathrm{LV}}\left(\cos\theta +1\right)=2\left(\sqrt{\gamma_{\mathrm{LV}}^{\mathrm{LW}}\gamma_{\mathrm{SV}}^{\mathrm{LW}}}+\sqrt{\gamma_{\mathrm{LV}}^{+}\gamma_{\mathrm{SV}}^{-}}+\sqrt{\gamma_{\mathrm{LV}}^{-}\gamma_{\mathrm{SV}}^{+}}\;\right),$$

The left side of Equation (3) is the Young–Dupre and the right side the van Oss et al. [4,17,26,27] equation expressing the adhesion work of liquid to the solid surface. For PTFE and PMMA and the water + ET solution of the RL and TX165 mixtures, Equation (3) assumes the following forms:

(4) $$\gamma_{\mathrm{LV}}\left(\cos\theta +1\right)=2\sqrt{\gamma_{\mathrm{LV}}^{\mathrm{LW}}\gamma_{\mathrm{PTFE}}^{\mathrm{LW}}},$$

and:

(5) $$\gamma_{\mathrm{LV}}\left(\cos\theta +1\right)=2\left(\sqrt{\gamma_{\mathrm{LV}}^{\mathrm{LW}}\gamma_{\mathrm{PMMA}}^{\mathrm{LW}}}+\sqrt{\gamma_{\mathrm{LV}}^{+}\gamma_{\mathrm{PMMA}}^{-}}\right),$$

In turn, Neumann et al. [13,28] suggested that the surface tension of the solid can be determined based on the contact angle only for one liquid on the given solid surface using the equation:

(6) $$\frac{\cos\theta +1}{2}=\sqrt{\frac{\gamma_{\mathrm{SV}}}{\gamma_{\mathrm{LV}}}}\exp\left[-\beta {\left(\gamma_{\mathrm{LV}}-\gamma_{\mathrm{SV}}\right)}^{2}\right],$$

where *β* is the constant.

According to Neumann et al. [13,28], this constant does not depend on the kind of liquid and solid and is equal to 0.00012 (m^2^/mJ)^2^.

The values of $\gamma_{\mathrm{SV}}$ for PTFE calculated from Equation (6) using the contact angle of the aqueous solution of ET, RL, and TX165 are lower than that determined from Equation (4) from the contact angle for *n*-alkanes (20.24 mN/m) [30] (Figures S2 and S3). However, these values do not change much as a function of the surfactant and ET concentrations and between the solutions of these compounds. It is difficult to explain exactly why the PTFE surface tension values calculated from the contact angle of the aqueous surfactant solutions are lower than those determined from the contact angle for *n*-alkanes.

The surface tension of ET, RL, and TX165, regardless of the orientation of their molecules towards the gas phase, is higher than the PTFE surface tension determined from the contact angles for *n*-alkanes. As suggested by Neumann et al. [13,28] and other authors, the adsorption of such substances on the solid surface should not reduce its surface tension. Thus, it can be concluded that the contact angle depends on the ratio of the AB to LW components of $\gamma_{\mathrm{SV}}$, rather than on its total value. That is why the calculated $\gamma_{\mathrm{SV}}$ values in the range of higher concentrations of ET, RL, and TX165 with the much lower AB/LW ratio are not slightly higher than in the range of lower concentrations of these compounds (Figures S2 and S3). A more- visible proof of this is the fact that the work of adhesion of the water solutions of RL and TX165 throughout their range is constant and close to the work of adhesion of water calculated from $2\sqrt{\gamma_{\mathrm{LV}}^{\mathrm{LW}}\gamma_{\mathrm{PTFE}}^{\mathrm{LW}}}$ (Figure S3). In the case of the aqueous ET solution, the work of its adhesion to PTFE is constant in the concentration range from 0 to 14.57 mol/dm^3^ and is slightly smaller than the work of water adhesion. However, taking into account the value of the ET surface tension and its components and parameters, as well as the measurement error of the surface tension and the contact angle, it does not seem that this difference between the work of adhesion of water and the aqueous ET solution contradicts the above-mentioned conclusion. The quoted facts suggest that the possible adsorption film of the substances does not affect the value of the contact angle of the water solutions of ET, RL, and TX165 on PTFE.

In the case of PMMA, the wetting behaviour of the aqueous solutions of ET, RL, and TX165 is more complicated than for PTFE. The values of $\gamma_{\mathrm{SV}}$ calculated from Equation (6) decrease as a function of the ET, RL, and TX165 concentrations, especially clearly in the range of their concentrations corresponding to the saturated monolayer at the solution–air interface (Figure S4a–c) [3,32,33]. However, in the concentration range of ET, RL, or TX165 corresponding to the unsaturated monolayer at the solution–air interface, the values of $\gamma_{\mathrm{SV}}$ calculated from Equation (5) are not much different from those determined based on the contact angle for water, formamide, and diiodomethane (Figure S4a–c) [30]. Moreover, $\gamma_{\mathrm{LV}}\left(\cos\theta +1\right)<2\left(\sqrt{\gamma_{\mathrm{LV}}^{\mathrm{LW}}\gamma_{\mathrm{PMMA}}^{\mathrm{LW}}}+\sqrt{\gamma_{\mathrm{LV}}^{+}\gamma_{\mathrm{PMMA}}^{-}}\right)$. This is particularly evident in the concentration range of ET, RL, or TX165 corresponding to the saturated monolayer of these compounds at the solution–air interface (Figure S4a–c) [3,32,33]. This fact and the values of the surface tensions of ET and the tails and heads of RL and TX165, which are higher than the surface tension of PMMA [30], suggest that, in the PMMA–solution drop–air system, a film is formed on the PMMA surface, apart from the drop deposited on it, either by adsorption of ET or by penetrating of the RL or TX165 particles from the drop onto the PMMA surface. As a result of such a process, there is a decrease in $\gamma_{\mathrm{SV}}$ by a value equal to the film pressure ($\pi_{\mathrm{SV}})$ on the PMMA surface, which should satisfy the condition:

(7) $$2\left(\sqrt{\gamma_{\mathrm{LV}}^{\mathrm{LW}}\gamma_{\mathrm{PMMA}}^{\mathrm{LW}}}+\sqrt{\gamma_{\mathrm{LV}}^{+}\gamma_{\mathrm{PMMA}}^{-}}\right)-\gamma_{LV}\left(\cos\theta +1\right)=\pi_{\mathrm{SV}},$$

Hence, the PMMA–solution interface tension fulfils the equation [2,3]:

(8) $$\gamma_{\mathrm{SL}}=\gamma_{\mathrm{SV}}-\pi_{\mathrm{SV}}-\gamma_{\mathrm{LV}}\cos\theta ,$$

In the case of the PTFE–solution interface tension, Equation (8) assumes the form [1]:

(9) $$\gamma_{\mathrm{SL}}=\gamma_{\mathrm{SV}}-\gamma_{\mathrm{LV}}\cos\theta ,$$

The difference between the PTFE (PMMA)–water interface tension ($\gamma_{\mathrm{SW}}$) and the PTFE (PMMA)–solution one ($\gamma_{\mathrm{SL}}$) can be treated as the adsorption layer pressure ($\pi_{SL})$.

Knowing the values of $\pi_{\mathrm{SV}}$ and $\pi_{\mathrm{SL}}$, it is possible to determine the concentration of a given surface active agent (Γ) at the solid–air and solid–solution interface, respectively, using, among others, the Frumkin equation, whose general form is as follows [1,7,34]:

(10) $$\pi =-R{T\Gamma }^{\max}\ln\left(1-\frac{\Gamma }{\Gamma^{\max}}\right),$$

where π is the difference between the solvent–air (solid) and solution–air (solid), as well as between the solid–air and solid covered by the adsorption layer–air interface tension, and $\Gamma^{\max}$ refers to the maximal possible adsorption of a given substance surface- active at a given interface.

The calculations performed using Equation (10) show that the concentrations of ET, RL, and TX165 at the PTFE–solution interface are close to that at the solution–air interface (Figure S5a,b). This fact confirms the conclusion drawn earlier based on the relationship between the adhesion and surface tension and the Lucassen–Reynders equation [35].

In the case of PMMA, the relationship between the adsorption of ET, RL, and TX165 at the interfaces including PMMA and the solution–air one is more complicated than for PTFE (Figure S6a–c). In the entire range of ET concentrations in the bulk phase, its concentration at the PMMA–A interface is the lowest and at the solution–air (S–A) interface is the highest (Figure S6a). The same relationship exists between the adsorption of RL and TX165 at different interfaces as in the case of ET, but only in the range of their concentrations in the bulk phase corresponding to the unsaturated monolayer at the S–A interface (Figure S6b,c). In the concentration range of RL and TX165 in the bulk phase corresponding to the saturated monolayer at the solution–air interface, their concentrations at the individual interfaces are similar. To explain this problem, the fraction of the area occupied by the given substrate ($X^{S}$) and the Gibbs standard free energy of adsorption were considered. The values of $X^{S}$ can be calculated from the equation having the form:

(11) $$X^{S}=\Gamma NA^{0},$$

where *N* is the Avogadro number and $A^{0}$ is the limiting area occupied by one molecule of the substance.

The ratio of the substance concentration in the bulk phase (C) to the fraction of the surface area occupied by that substance is equal to the constant a in the Langmuir isotherm equation ($a=\frac{C}{X^{S}}$). The constant a is related to the standard Gibbs adsorption free energy ($\Delta G_{\mathrm{ads}}^{0}$) based on the equation [1]:

(12) $$a=\frac{C}{X^{S}}=\omega \exp\left(\frac{\Delta G_{\mathrm{ads}}^{0}}{RT}\right),$$

where ω is the number of solvent moles in 1 dm^3^.

The calculations of the concentration ratio in the bulk phase of a given surfactant to the fraction of the surface area occupied by it showed that, in the case of ET, this ratio is not constant and increases with the increasing concentration, and it changes slightly for RL and TX165 in the concentration range from zero to CMC (Figure S7a–c). Moreover, in the case of RL and TX165, $\frac{C}{X^{S}}$ does not depend on the type of interface and the polymer (Figure S7b,c). It should be pointed out that, in the case of ET, the sum of moles of water and ET in 1 dm^3^ decreases with the increasing ET concentration and that the ET activity is not equal to the mole fraction in the bulk phase [33]. Probably, in the range of ET concentration from 0 to 0.01 M at which, similar to surfactants, the number of the water moles can be assumed to be constant, its $\frac{C}{X^{S}}$ value should be also constant. However, in such a concentration range, changes in the surface tension are small and difficult to measure precisely. Within this concentration range, ET can be treated as a co-surfactant, but not as a co-solvent [1]. It turned out that, in contrast to $\frac{C}{X^{S}}$, the ratio of the ET activity to the fraction of the area occupied by it changes to a small extent as a function of the ET concentration in the bulk phase and does not depend on the type of interface and polymer (Figure S7a).

As a matter of fact, the $\frac{C}{X^{S}}$ values influence the Gibbs standard free energy of adsorption (Figure S8a–c). For RL and TX165, two series of $X^{S}$ values were used to calculate $\Delta G_{\mathrm{ads}}^{0}$ because they depend on the surfactant molecules’ orientation at the interfaces. As was shown earlier, the ET molecules occupy the same area at the interfaces regardless of their orientation because they can be inscribed in an equilateral cube [36]. However, the molecules of RL and TX165 occupy different areas depending on their orientation towards the interface [3,32,36]. It turned out that the calculated $X^{S}$ values for the vertical and horizontal orientations of the RL and TX165 molecules are adequate for their concentrations at different interfaces.

It follows that, for ET, $\Delta G_{\mathrm{ads}}^{0}$ depends on the type of interface at which its molecules adsorb, as well as the type of polymer and concentration (Figure S8a). In the case of RL and TX165, there are insignificant differences between the $\Delta G_{\mathrm{ads}}^{0}$ values calculated at their molecules’ vertical and horizontal orientations at the given interfaces, as well as depending on the type of interface and polymer (Figure S8b,c). It is known that the tendency to adsorb of a given surfactant at the interface depends, among others, on the number of the water molecules surrounding the tail and head of the surfactant molecules, as well as their interactions with the solid surface. In the case of ET, the small number of water molecules surround its molecules, and probably, the interactions between the ET molecules and PTFE or PMMA play a main role in the ET adsorption process. As these interactions are considerably different for PMMA compared to PTFE, the differences in $\Delta G_{\mathrm{ads}}^{0}$ were observed. In the case of TX165 and RL, probably, the change in the free energy of the system related to that in the number of water molecules in contact with the RL and TX165 molecules plays a dominant role, rather than the energy of the interactions with the surface of PTFE and PMMA.

### 2.2. Wetting and Adsorption Properties of the ET, RL, and TX165 Mixture

**Figure 4:**
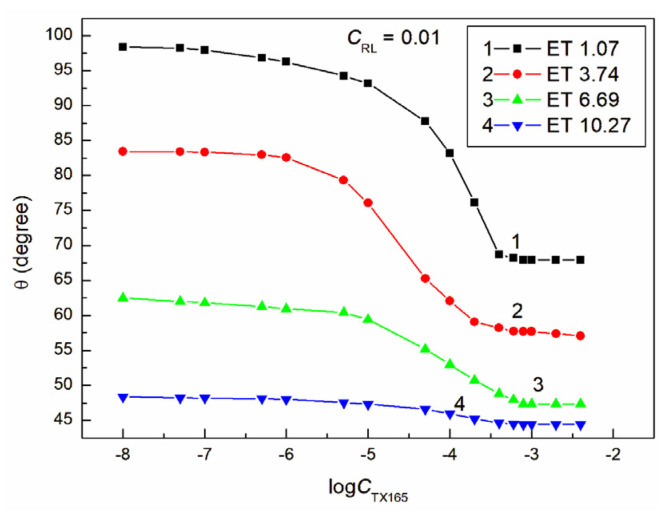
A plot of the contact angle (θ) of the ET + RL + TX165 mixture aqueous solutions on PTFE vs. the logarithm of the TX165 concentration $\left(\log C_{\mathrm{TX}165}\right)$ at a constant RL concentration equal to 0.01 mg/dm^3^. Curves 1–4 correspond to the ET constant concentrations equal to 1.07, 3.74, 6.69, and 10.27 mol/dm^3^.

**Figure 5:**
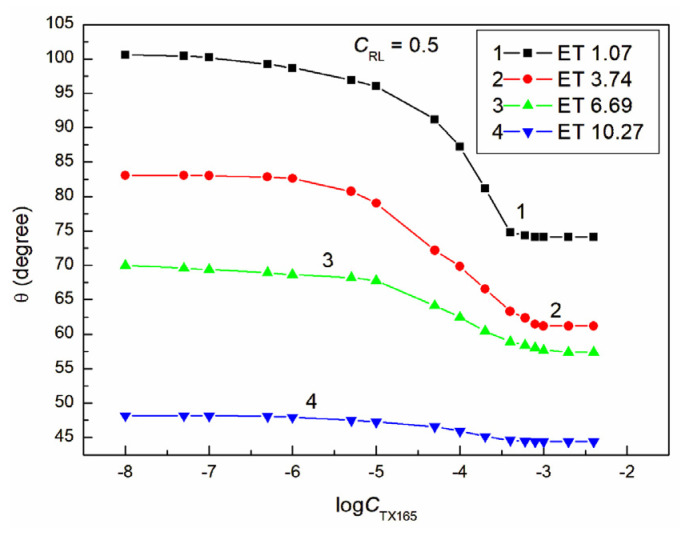
A plot of the contact angle (θ) of the ET + RL + TX165 mixture aqueous solutions on PTFE vs. the logarithm of the TX165 concentration $\left(\log C_{\mathrm{TX}165}\right)$ at a constant RL concentration equal to 0.5 mg/dm^3^. Curves 1–4 correspond to the ET constant concentrations equal to 1.07, 3.74, 6.69, and 10.27 mol/dm^3^.

**Figure 6:**
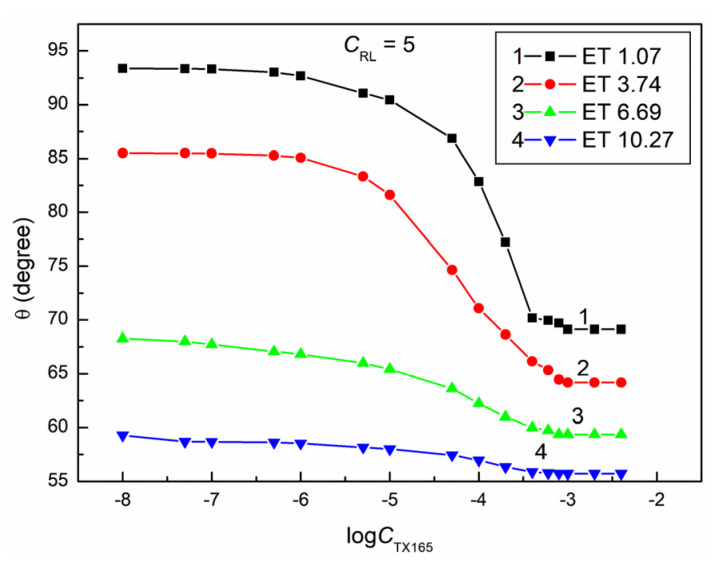
A plot of the contact angle (θ) of the ET + RL + TX165 mixture aqueous solutions on PTFE vs. the logarithm of the TX165 concentration $\left(\log C_{\mathrm{TX}165}\right)$ at a constant RL concentration equal to 5 mg/dm^3^. Curves 1–4 correspond to the ET constant concentrations equal to 1.07, 3.74, 6.69, and 10.27 mol/dm^3^.

**Figure 7:**
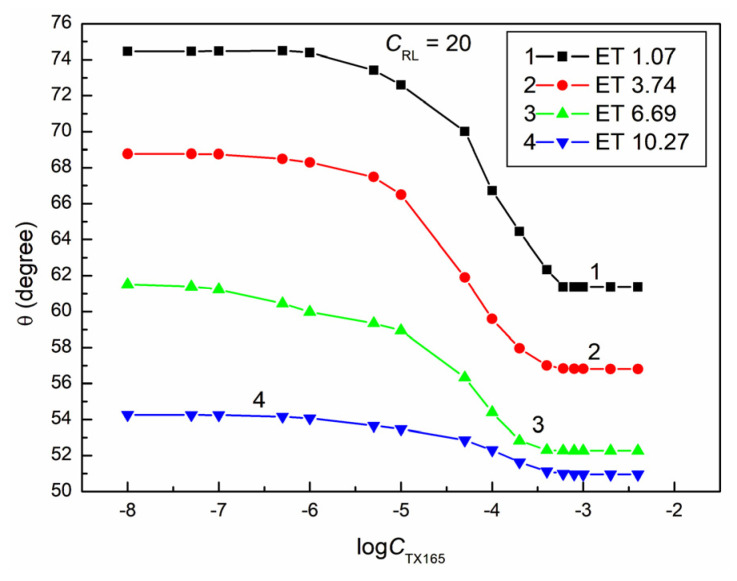
A plot of the contact angle (θ) of the ET + RL + TX165 mixture aqueous solutions on PTFE vs. the logarithm of the TX165 concentration $\left(\log C_{\mathrm{TX}165}\right)$ at a constant RL concentration equal to 20 mg/dm^3^. Curves 1–4 correspond to the ET constant concentrations equal to 1.07, 3.74, 6.69, and 10.27 mol/dm^3^.

**Figure 8:**
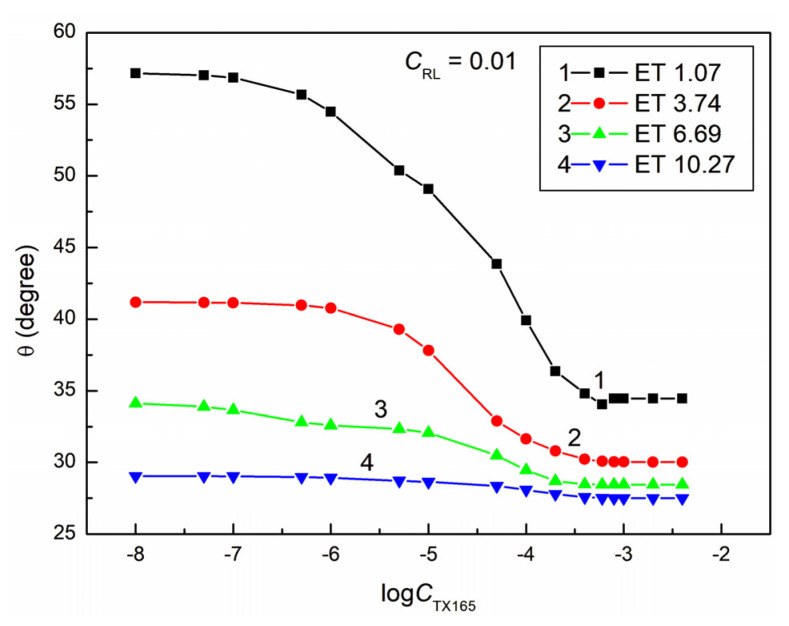
A plot of the contact angle (θ) of the ET + RL + TX165 mixture aqueous solutions on PMMA vs. the logarithm of the TX165 concentration $\left(\log C_{\mathrm{TX}165}\right)$ at a constant RL concentration equal to 0.01 mg/dm^3^. Curves 1–4 correspond to the ET constant concentrations equal to 1.07, 3.74, 6.69, and 10.27 mol/dm^3^.

**Figure 9:**
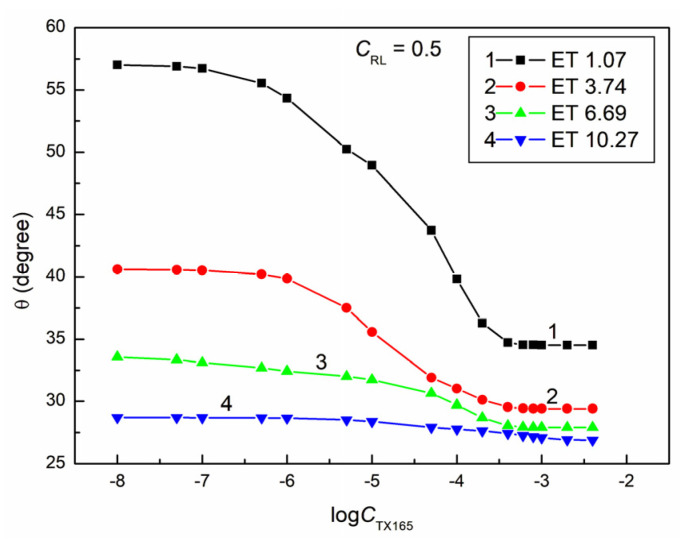
A plot of the contact angle (θ) of the ET + RL + TX165 mixture aqueous solutions on PMMA vs. the logarithm of the TX165 concentration $\left(\log C_{\mathrm{TX}165}\right)$ at a constant RL concentration equal to 0.5 mg/dm^3^. Curves 1–4 correspond to the ET constant concentrations equal to 1.07, 3.74, 6.69, and 10.27 mol/dm^3^.

**Figure 10:**
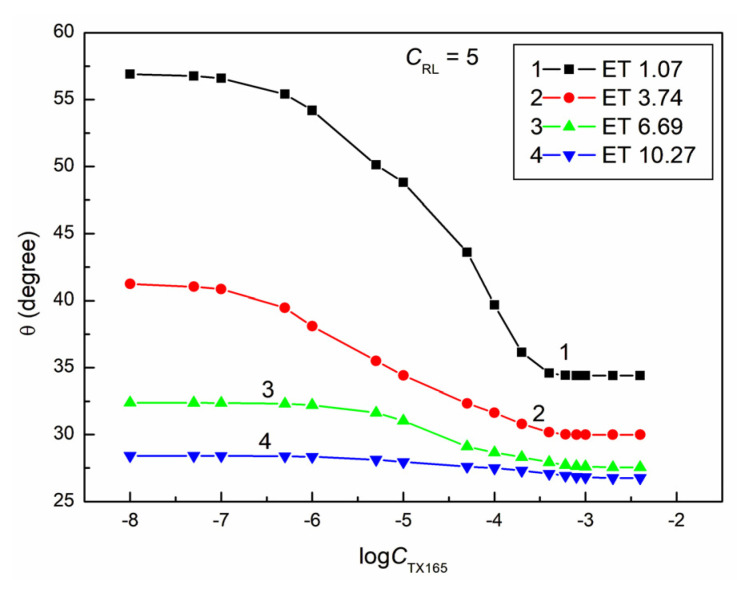
A plot of the contact angle (θ) of the ET + RL + TX165 mixture aqueous solutions on PMMA vs. the logarithm of the TX165 concentration $\left(\log C_{\mathrm{TX}165}\right)$ at a constant RL concentration equal to 5 mg/dm^3^. Curves 1–4 correspond to the ET constant concentrations equal to 1.07, 3.74, 6.69, and 10.27 mol/dm^3^.

**Figure 11:**
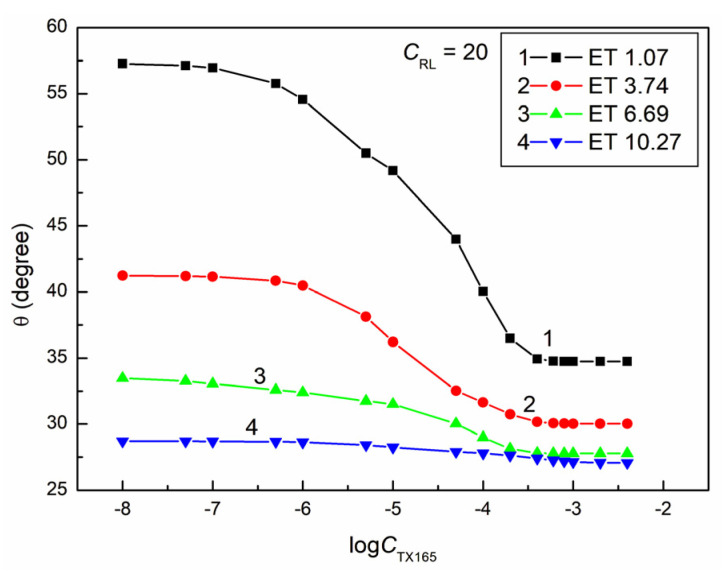
A plot of the contact angle (θ) of the ET + RL + TX165 mixture aqueous solutions on PMMA vs. the logarithm of the TX165 concentration $\left(\log C_{\mathrm{TX}165}\right)$ at a constant RL concentration equal to 20 mg/dm^3^. Curves 1–4 correspond to the ET constant concentrations equal to 1.07, 3.74, 6.69, and 10.27 mol/dm^3^.

The wetting behaviour of the mixture of ET, RL, and TX165 depends on the adsorption of the individual components of the mixture at three interfaces, as well as on the packing and orientation of their molecules in the adsorption layers. Considering the contact angle of the solution including water, ET, RL, and TX165 on the surface of PTFE and PMMA (Figure 4, Figure 5, Figure 6, Figure 7, Figure 8, Figure 9, Figure 10 and Figure 11) in terms of the adsorption of ET, RL, and TX165 at different interfaces, we can encounter the problem of describing the adsorption process. In the concentration range of ET from 0 to about 0.01 mol/dm^3^, it can be treated as a co-surfactant. Within this range of ET concentrations, a solution containing water, ET, RL, and TX165 can be treated as an aqueous solution of the ET + RL + TX165 mixture. In such a solution, the chemical potential of ET can be defined asymmetrically, i.e., as the concentration of ET is zero, the activity coefficient is equal to unity. However, the ET concentration used in our studies was much greater than 0.01 mol/dm^3^, and therefore, water with ET should be treated as a mixture. In this case, the chemical potential of ET should be determined symmetrically, i.e., the ET activity coefficient tends to unity if the ET fraction also tends to unity. Therefore, as was shown earlier, the ratio of the molar concentration of ET to the fraction of the area occupied by it at different interfaces is not constant, but depends on the ET concentration in the bulk phase. Thus, a solution containing water, ET, RL, and TX165 should be treated as an aqueous ethanol solution of the RL with TX165 mixture. This indicates that the competition between water and ET molecules in their contact with the tails and heads of the RL and TX165 molecules is of decisive importance in the adsorption of the RL and TX165 molecules at different interfaces, and this, in turn, affects the wetting process.

The proof of this can be the fact that, even at a constant ET concentration equal to 1.07 M and RL equal to 0.01 mg/dm^3^ and a variable concentration of TX165 from 0 to 4 × 10^−3^ mol/dm^3^, the minimal contact angle of the water–ethanol solution of the RL and TX165 mixture on PTFE is already 10 ° smaller than that for the aqueous TX165 solution (Figure 2 and Figure 4). However, at a constant ET concentration of 10.27 mol/dm^3^ and RL equal to 0.01 mg/dm^3^, the minimum value of the contact angle for the water–ethanol solution of the ET and RL mixture is more than 30° lower than the minimum value of the individual TX165 aqueous solution (Figure 1, Figure 2 and Figure 4). It is interesting that the value of the contact angle for the mixture of water and ET at an ET concentration equal to 10.27 mol/dm^3^ on PTFE without the surfactants is 57.1° and for the aqueous solution of RL at a concentration equal to 0.01 mg/dm^3^ about 111°, and the minimum value for the TX165 solution is 78.2°. The minimum value of the contact angle of the water–ethanol solution of the mixture of RL and TX165 on the PTFE surface is equal to 44.4° (Figure 4). This fact suggests synergism in the process of wetting PTFE with the water–ethanol solution of the RL and TX165 mixture. Unfortunately, an increase in the RL concentration in the solution with the same ET concentration reduces the synergistic effect of the PTFE wettability (Figure 4, Figure 5, Figure 6 and Figure 7). It is worth noting that the shape of all contact angle isotherms for PTFE is similar to that of the surface tension isotherms [36]. Therefore, the isotherms of the contact angle can be also described by the second-order exponential function (for example, Figure S9).

Most isotherms of the contact angle of the water–ethanol solution of the RL mixture with TX165 for PMMA (Figure 8, Figure 9, Figure 10 and Figure 11), similarly to PTFE, can be described by the second-order exponential function (example in Figure S9). Probably, the constants of the exponential functions describing the isotherms of the contact angle of the studied solutions for PTFE and PMMA depend on the components and parameters of the surface tension of all components of the PTFE (PMMA)–solution droplet–air system. It is interesting that, for all solutions including water, ET, RL, and TX165, with the exception of the solutions in which the constant ET concentration is equal to 10.27 mol/dm^3^, the minimum value of the contact angle on the PMMA surface is smaller than the contact angle of water solution of individual ET, RL, and TX165 at the concentration the same as in the studied solutions. For example, in the case of the solution at a constant concentration of RL equal to 0.01mg/dm^3^ and ET to 1.07 mol/dm^3^ and the variable concentration of TX165 from 0 to 4 × 10^−3^ mol/dm^3^, the minimal contact angle is equal to 34.5°. For such a system, the contact angle of individual solutions of ET and RL is equal to 73.2 and 66.8°, respectively. In turn, the minimal value of the contact angle for the individual aqueous solutions of TX165 is equal to 49.5° [2,3]. This fact proves the occurrence of a synergistic effect in the process of PMMA wetting by the water–ethanol solution of the RL and TX165 mixture.

The value of the contact angle for the W + ET solution of RL + TX165 on the PTFE or PMMA surfaces depends on the concentration and orientation of the ET, RL, and TX165 molecules in the surface layers at the solution–air, PTFE (PMMA)–air and PTFE (PMMA)–solution interfaces. The preliminary information on the cumulative ET, RL, and TX165 concentrations at the mentioned interfaces can be determined based on the dependence of the cosine of the contact angle and the reciprocal of the surface tension of the solution (Figure S10a,b).

In the case of PTFE, for all series of the solutions used in the studies at the constant concentration of ET and RL and the variable one of TX165, there is a linear relationship between the cosine of the contact angle and the reciprocal of the surface tension of the solution (($\cos\theta =m\frac{1}{\gamma_{\mathrm{LV}}}+k$) (Figure S10a). The constants k and m in the linear equation for the particular series of solutions differing in the values of the constant concentrations of ET and RL are close to each other. The average value of k is close to −1 and m up to 47 mJ/m^2^. According to the Young–Dupre equation (Equation (2)), this shows that the value m is equal to the work of adhesion of the solution to the PTFE surface and that this work depends on the composition and concentration of the water–ethanol solution of the RL and TX165 mixture to a small extent. Such values of the constants k and m also prove that the surface tension of PTFE after drop of solution is deposited on it does not change with the change of the composition and concentration of the solution. As mentioned above, t his is consistent with the suggestion made by Fowkes, Good, van Oss, and other authors that the substances with a higher surface tension than that of a solid one cannot reduce its surface tension [12,16,17,26,27]. This condition is satisfied for the PTFE–solution drop–air systems studied by us.

The relationship between the cosine of the contact angle and the reciprocal of the surface tension of the solution for PMMA is different compared to PTFE (Figure S10a,b). In the case of PMMA, there is not a linear relationship between cosθ and $\frac{1}{\gamma_{\mathrm{LV}}}$ for all series of the solutions. Moreover, the constant k in this relationship is different from −1. For this reason, the constant m does not equal the work of adhesion of the solution to PMMA. This constant can only prove that the work of adhesion of some series of solutions to the PMMA surface is constant. On the other hand, the nonlinear relationships prove that this work is not constant and that a change of the PMMA surface tension as a function of the TX165 concentration in the solution cannot be excluded.

The fact that the surface tension of PTFE does not change, but PMMA changes as a function of the TX165 concentration at the constant concentration of RL and ET was confirmed by the calculation of this tension using the Neumann et al. [13,28] equation (Equation (6)) (Figures S11a–S18d). For all the PTFE–solution drop–air systems, the calculated values of the PTFE surface tension are practically constant in the range of the TX165 concentration from 0 to 4 × 10^−3^ mol/dm^3^. However, these values depend on the constant concentration of RL and ET (Figures S11a–S14d). In most cases, similar to the solutions of individual components of the mixture, the surface tension of PTFE calculated from Equation (6) differs from that calculated from the angles of hydrocarbons applying the van Oss et al. equation [4,17,26,27]. On the other hand, the work of adhesion of the water–ethanol solution of RL and TX165 of the mixture ($W_{a}$) is constant in the range of the tested TX165 concentration and does not depend on the constant concentration of ET and RL. Moreover, the $W_{a}\;$values for PTFE calculated directly from the Young–Dupre equation are close to those obtained from the relationship between the cosine of the contact angle and the reciprocal of the solution surface tension and to those determined from the van Oss et al. [4,17,26,27] equation for water (Figures S11a–S14d). This fact indicates that the LW component of the tested series of solutions is constant regardless of the composition and concentration of the solution components. This means that the changes in the contact angle in the PTFE–solution drop –air system result only from those in the AB component of the solution as a function of the composition and concentration of its components. The convergence of the $W_{a}$ value for PTFE solutions with that of the water adhesion work to its surface calculated based on the PTFE surface tension determined from the contact angles of hydrocarbons [30] and the LW component of the water surface tension [30] proves that it is possible to determine the PTFE–solution interface tension based on the Young equation ($\gamma_{\mathrm{SL}}=\gamma_{\mathrm{SV}}-\gamma_{\mathrm{LV}}\cos\theta$) [1,7] (Figures S11a–S14d). In turn, according to the thermodynamic definition of the work of adhesion, the PTFE–solution interface tension fulfils the equation [7]:

(13) $$\gamma_{\mathrm{SL}}=\gamma_{\mathrm{SV}}+\gamma_{\mathrm{LV}}-W_{a},$$

According to van Oss et al. [4,17,26,27], $\gamma_{\mathrm{LV}}=\gamma_{\mathrm{LV}}^{\mathrm{LW}}+\gamma_{\mathrm{LV}}^{\mathrm{AB}}$. Hence:

(14) $$\gamma_{\mathrm{SL}}=\gamma_{\mathrm{SV}}+\gamma_{\mathrm{LV}}^{\mathrm{LW}}+\gamma_{\mathrm{LV}}^{\mathrm{AB}}-W_{a},$$

Because, as mentioned above, $\gamma_{\mathrm{SV}}$, $\gamma_{\mathrm{LV}}^{\mathrm{LW}}$, and $W_{a}$ for the studied PTFE–solution-drop–air systems are constant, we can write:

(15) $$\gamma_{\mathrm{SL}}=\gamma_{\mathrm{LV}}^{\mathrm{AB}}+l,$$

where *l* is constant.

As follows from Equation (15), the changes of the PTFE–solution interface tension as a function of the TX165 concentration in the range of the studied ET, RL, and TX165 concentrations occurred only due to the decrease of the AB component of the solution surface tension.

The determination of the relationship between the PMMA–solution interface tension and the solution surface tension, or more precisely, the AB component of this tension, for the solutions of different compositions and concentrations is more complicated than for the PTFE–solution. The $\gamma_{\mathrm{SV}}$ values for PMMA calculated from the Neumann equation (Equation (6)) vary significantly as a function of the TX165 concentration at the given composition and the constant ET and RL concentration (Figures S15a–S18d). As a function of the TX165 concentration with the constant composition and the concentration of RL and ET, the work of adhesion of the solution to the PMMA surface, calculated from both the Young–Dupre equation and van Oss et al., also changes considerably (Figures S15a–S18d). In the case of the solutions of the individual mixture components, this indicates that, after setting a drop of the solution on the PMMA surface, a film is formed, causing a change in the surface tension of PMMA. In such a case, $\gamma_{\mathrm{SL}}=\gamma_{\mathrm{SV}}-\pi -\gamma_{\mathrm{LV}}\cos\omega \theta$. Taking into account that $\gamma_{\mathrm{SV}}$ and $\gamma_{\mathrm{LV}}^{\mathrm{LW}}$ are constant, it can be written:

(16) $$\gamma_{\mathrm{SL}}=z-\pi +\gamma_{\mathrm{LV}}^{\mathrm{AB}}-W_{a},$$

where *z* is the constant.

As mentioned above, the π value should be equal to the difference between the work of adhesion of the solution to PMMA calculated from the van Oss et al. [4,17,26,27] equation and the work calculated from the Yung-Dupre equation (Figures S15a–S18d).

The knowledge of $\gamma_{\mathrm{SL}}$ for the PTFE–solution and PMMA–solution and π allowed calculating the concentration of individual components of the solution at the PTFE (PMMA)–solution and PMMA–air interfaces (Figures S19a–S26d) using the Frumkin equation (Equation (10)). For the calculation of Γ for ET, RL, and TX165 instead of π (the sum of the pressure of all components of the solution), $\pi_{\mathrm{ET}}$, $\pi_{\mathrm{RL}}$, and $\pi_{\mathrm{TX}}$ were used in the Frumkin equation, respectively. The sum of the pressure of ET, RL, and TX165 can be expressed as [36]:

(17) $$\pi =\sum_{i=n}^{i=1}\frac{\pi_{i}}{\sum_{i=n}^{i=1}\pi_{i}}\pi ,$$

where $\pi_{i}$ is the film pressure of the given component of the mixture at the proper concentration in the absence of other components, $\frac{\pi_{i}}{\sum_{i=n}^{i=1}\pi_{i}}$ is the fraction of the given component in the mixed layer at the interface, and *n* is the number of components in the mixture.

The values of $\pi_{\mathrm{ET}}$, $\pi_{\mathrm{RL}}$, and $\pi_{\mathrm{TX}}$ calculated from Equation (17) were used in Equation (10) for the determination of the ET, RL, and TX165 concentrations of the PTFE–solution, PMMA–solution, and PMMA–air interfaces (Figures S19a–S26d).

This proved that the sum of the concentrations of ET, RL, and TX165 at the PTFE–solution interface is close to that at the solution–air interface [36] (Figures S19a–S22d). However, the concentration of individual solution components was not the same for all solution series. This may be due to the fact that the adsorption of mixtures at the PTFE–solution interface can result additionally from the competitive interactions of all components of the solution with the PTFE surface. It should also be emphasised that for, some series of solutions, the total concentration is higher than the maximum concentration of individual components of the solution. This may result from the fact that, apart from self-adsorption, the ET molecules can also combine with the RL and TX165 molecules instead of the water molecule, and thus, the total adsorption of solution components increases.

As mentioned above, in the case of PMMA, the concentrations of ET, RL, and TX165 were calculated not only at the PMMA–solution, but also at the PMMA–air interfaces (Figures S23a–S26d). In general, it can be said that the adsorption of ET, RL, and TX165 at the PMMA–air interface is smaller than at the PMMA–solution one. For most series of solutions, the total adsorption of ET, RL, and TX165 at the PMMA–solution interface is similar to that at the solution–air interface [36]. With the exception of the series of solutions with the constant concentration of ET equal to 1.07 mol/dm^3^ and the RL concentration equal to 0.01 and 0.5 mg/dm^3^, on the isotherms of the total concentration of ET + RL + TX165, there are maxima at the PMMA–solution and PMMA–air interfaces. This explains the lack of a linear relationship between cosθ and $\frac{1}{\gamma_{\mathrm{LV}}}$ and indicates a change in the behaviour of ET molecules at the interfaces as a function of the TX165 concentration. Probably, in the range of the TX165 concentrations from zero to that corresponding to the maximum on the isotherms of the total concentration of the solution components, ET is adsorbed separately and together with the RL and TX165 molecules. Above this concentration, the ET adsorption occurs only in the RL and TX165 mixing layer. This may result from stronger interactions between the RL and TX65 molecules and PTFE than the PTFE–ET ones. This may be confirmed by a decrease in the concentration of ET at the interfaces involving PMMA with an increase in the concentration of TX165. On the basis of the Frumkin isotherms of the concentration of particular components of the solution, it is possible to determine the fraction of the interface area occupied by a given component using Equation (11). It appeared that in, many cases, the sum of $X^{S}$ values of ET, RL, and ET was higher than unity, which confirmed the suggestion that the ET layer can be formed on the RL and TX165 mixture layer. This can be observed at all studied interfaces.

Knowing the $X^{S}$ values, it is possible to determine the values of $\frac{C}{X^{S}}$ (Figures S27a–S32b). According to Langmuir, this value should be constant in the surfactant range concentration in the surface layer from zero to the maximum. For calculations of $X^{S}$ for RL and TX165, the limiting area occupied by one molecule was used in its parallel and perpendicular orientations towards the interfaces. Indeed, in the parallel orientation, only the tails of EL and TX165 were taken into account. The $\frac{C}{X^{S}}$ values for ET, RL, and TX165 at the PTFE–solution, PMMA–solution, and PMMA–air interfaces depend on their concentrations and depend on the mutual influence. Indeed, the $\frac{C}{X^{S}}$ values for RL and TX165 depend on the limiting area occupied by one molecule used for their calculations (Figures S27a–S32b). It should be emphasised that the $\frac{C}{X^{S}}$ values for TX165 are constant in the range of its concentration, corresponding to the unsaturated monolayer at the solution–air interface (Figures S27d,e, S31a and S32b) However, for the constant RL and ET concentrations, there is a continuous increase of the $\frac{C}{X^{S}}$ values in the range of the changing TX165 concentration from 0 to 4 × 10^−3^ mole/dm^3^.

Knowing the $\frac{C}{X^{S}}$ values (Figures S27a–S32b), it is possible to determine the standard Gibbs free energy of adsorption of each component of the solution at the solid–solution and solid–air interfaces (Figures S32a–S36d). As follows from the calculations, the $\Delta G_{\mathrm{ads}}^{0}$ of TX165 depends on the constant concentrations of RL and ET only slightly and is almost the same at the PTFE–solution, PMMA–solution, PMMA–air, and solution–air interfaces (Figure S33d,e) [36]. In fact, the absolute values of $\Delta G_{\mathrm{ads}}^{0}$ for TX165 are constant only in the small range of the TX165 concentrations and increase as a function of the concentration. The $\Delta G_{\mathrm{ads}}^{0}$ for ET and in contrast to TX165 depends on the value of their constant concentrations significantly. At the concentrations of RL equal to 0.01 and 0.5 mg/dm^3^, the values of $\Delta G_{\mathrm{ads}}^{0}$ are similar for all studied interfaces. The same can be stated for ET at its constant concentration equal to 1.07 mol/dm^3^. The TX165 influences the $\Delta G_{\mathrm{ads}}^{0}$ of RL and ET to a greater extent than vice versa.

## 3. Materials and Methods

### 3.1. Materials

The polytetrafluoroethylene (PTFE) and poly (methyl methacrylate) (PMMA) were purchased from Mega-Tech, Tomaszow Mazowiecki, Poland. The procedure of the PTFE and PMMA plates’ preparation for the contact angle measurements of the water + ethanol solution of rhamnolipid (RL) with the Triton X-165 (TX165) mixture on their surface was the same as described in the literature [30]. The surface topography of the chosen plates was monitored using an optical profilometer (Contour GT, Veeco, Plainview, NY, USA) and atomic force microscopy (AFM) (Nanoscope 3, Veeco, Plainview, NY, USA). For the contact angle measurements, the plates with the smallest roughness were used (PTFE: Ra ≈ 6 nm, Rq ≈ 7.5 nm (AFM), PMMA: Ra ≈ 1.5 nm, Rq ≈ 2 nm (AFM)). Additionally, the contact angles (θ) of water (W), diiodomethane (D), and formamide (F) on the chosen plates were measured to check the quality of the plates (θ for PTFE: W = 111.1° ± 1; D = 74.7° ± 1.0; F = 91.8° ± 1.1; θ for PMMA: W = 74.25° ± 1.3; D = 36.37° ± 1.1; F = 55.56° ± 1.2).

For the solution preparation, Triton X-165 (p-(1,1,3,3-tetramethylbutyl)-phenoxypolyoxyethylene glycol) of a purity greater than 99% and rhamnolipid (95%) (RL) were purchased from FLUKA and Sigma-Aldrich (St. Louis, MO, USA), respectively. They were used without further purification. Ethanol (ET) (96% pure) bought from POCH, Gliwice, Poland, was purified by the method described earlier [37].

The water used for the solution preparation was doubly distilled and deionised (Destamat Bi18E, Inkom Instruments, Warsaw, Poland); its surface tension at 293 K was 72.8 mN/m, and the internal specific resistance was 18.2⋅× 10^6^ Ω⋅m.

The water + ET solutions of the RL and TX165 mixture were prepared at the constant concentration of ET + RL and the variable TX165 concentration from 0 to 4 × 10^−3^ mol/dm^3^. Four series of constant ET + RL mixture concentrations were applied. Each series contained four cases of constant concentration of the ET + RL mixture of different compositions. The first series included the mixtures of RL + ET at the RL concentration equal to 0.01 mg/dm^3^, corresponding to the unsaturated monolayer at the water–air interface ($C_{\mathrm{RL}}^{\mathrm{unsat}}$) and the ET concentration equal to 1.07, 3.74, 6.69, and 10.27 mol/dm^3^. The ET concentration of 1.07 mol/dm^3^ corresponds to its unsaturated monolayer at the water–air interface ($C_{\mathrm{ET}}^{\mathrm{unsat}}$), 3.74 mol/dm^3^ to the maximal Gibbs surface excess concentration ($C_{\mathrm{ET}}^{\max,G}$), 6.69 mol/dm^3^ to the critical aggregation concentration (CAC), and 10.27 mol/dm^3^ to the concentration in the bulk phase at which the ET monolayer includes only ET ($C_{\mathrm{ET}}^{\mathrm{total},s}$). The second, third, and fourth series contained the same ET concentration, and the RL concentration was 0.5, 5, and 20 mg/dm^3^. The concentrations of RL equal to 0.5, 5, and 20 mg/dm^3^ correspond to the smallest concentration at which the saturated monolayer of RL at the water–air interface is formed ($C_{\mathrm{RL}}^{\mathrm{sat},1}$), the concentration in the range of the saturated RL monolayer ($C_{\mathrm{RL}}^{\mathrm{unsat},2}$), and the concentration close to the CMC.

### 3.2. Methods

The advancing contact angles (θ) of the water + ET solution of the RL and TX165 mixture at the constant concentration of ET + RL and the variable one of TX165 on the PTFE and PMMA surfaces were measured using the sessile drop method. The contact angle measurements were conducted at 293 ± 0.1 K applying the DSA30 measuring system (Krüss, Hamburg, Germany), equipped with the thermostated chamber. The procedure and conditions used for the contact angle measurements were described in the literature [30]. In each experiment, the measuring chamber was saturated by the atmosphere in order to avoid evaporation. For each constant concentration of ET + RL and the variable one of TX165, the contact angle of the water +ET solution of the RL + TX165 mixture on PTFE and PMMA was measured for 30 drops. The standard deviation of the contact angle values was in the range from 1 to 2°.

## 4. Conclusions

The contact angle isotherms of the aqueous ethanol (ET), rhamnolipid (RL), and Triton X-165 (TX165) solutions on the PTFE and PMMA surfaces can be described by the second-order exponential function, however, in the case of the water + ethanol solution of the RL and TX165 mixture, only at low constant RL and ET concentrations.

The cosine of the contact angle of the water + ethanol solution of the RL and TX165 mixture on the PTFE surface changes linearly as a function of the reciprocal of the surface tension of this solution at the constant concentrations of ET and RL and the variable TX165 concentration.

The constants of the linear function describing the isotherms of the contact angle in the PTFE–solution drop–air system as a function of the reciprocal of solution surface tension do not depend on the values of the constant concentrations of RL and ET.

The slope of the linear dependence between the cosine of the contact angle and the reciprocal of the solution surface tension is close to the adhesion work of the solution to the PTFE surface calculated from the Young–Dupre equation, as well as to that of water to PTFE.

The changes of PTFE–solution interface tensions as a function of the TX165 concentration at the constant ET and RL concentrations depend only on the acid–base component of the solution surface tension.

In the case of PMMA, there is no linear dependence between the contact angle of the solution and the reciprocal of the surface tension of the solution. This depends largely on the constant concentrations of RL and ET.

There are differences between the adhesion work of the solution to the PMMA surface calculated from the van Oss et al. equation and from the Young–Dupre one, which are equal to the film pressure formed behind the solution drop settled on the PMMA surface.

The PMMA–solution interface tension, contrary to the PTFE–solution one, depends not only on the acid–base component of the solution surface tension, but also on the film pressure and adhesion work.

The total adsorption of ET + RL + TX165 at the PTFE–solution and PMMA–solution interfaces is comparable to that at the solution–air interface, but the adsorption at the PMMA–air interface is lower than at the solution–air one.

The ratio of the RL and TX165 concentration to the fraction of the area occupied by RL and TX165 at different interfaces is constant only in a small range of their concentration, but for ET increases strongly as a function of its concentration.

The standard Gibbs free energy of adsorption of particular components of the water + ethanol solution of the RL and TX165 mixture at different interfaces is comparable to their adsorption from the individual aqueous solutions.

The obtained results and their thermodynamic analysis can be helpful for better understanding the dependence of the process of wetting solids by multicomponent solutions and the process of adsorption at different interfaces.

The proposed mechanism of adsorption of ethanol, rhamnolipid, and Triton X165 at the polymer–air and polymer–water–ethanol solution of the rhamnolipid and TX165 mixture interfaces can be used for the formation of antitoxic and antibacterial layers on the polymer implants.

## Figures and Tables

**Table 1:** Components and parameters of the ethanol, TX165, RL, PTFE, and PMMA surface tension at 293 K and the maximal concentration at the water–air interface and the limiting area occupied by water, TX165, ET, and RL.

Substance	$\gamma_{LV}^{LW}$ (mN/m)	$\gamma_{LV}^{+}$ (mN/m)	$\gamma_{LV}^{-}$ (mN/m)	$\gamma_{LV}^{AB}$ (mN/m)	$\gamma_{LV}$ (mN/m)	$A_{0}$ (Å^2^)	$\Gamma^{max}$ (×10^−6^ mol/m^2^)	$\Gamma^{0}$ (×10^−6^ mol/m^2^)	Ref.
Water from $\gamma_{\mathrm{WH}}$	21.80	25.60	25.50	51.00	72.80	10.00	16.60	16.60	[12,29]
Water from *θ*	26.85	22.975	22.975	45.95	72.80				[30]
Ethanol	21.40	0.09	9.00	1.8	24.20	21.00	7.91	7.91	[2]
TX-165 tail	22.00	-	-	-	22.00	35.70	2.12	4.65	[3]
TX-165 head	27.70	0.33	50.20	8.14	35.84				[3]
Rhamnolipid tail	21.80	-	-	-	21.80	69.09	2.01	2.403	[2]
Rhamnolipid head	35.38	0.04	56.74	3.01	38.39				[2]
PTFE	20.24	-0.00	0.00	0.00	20.24	-	-	-	[30]
PMMA	41,28	0.00	7.28	0.00	41.28	-	-	-	[30]

**Table 2:** The constant of the linear functions describing the relationship between the cosine of the contact angle and the reciprocal of the surface tension.

Compound	PTFE		PMMA	
	*k*	*m*	*k*	*m*
ET	−0.99189	43.72971	−0.16401	31.72324
RL	−1.00545	45.88958	-	-
TX165	−1.03069	48.49819	−0.17339	32.54060

## Data Availability

The data presented in this study are available in the Supplementary Materials.

## Supplementary Materials

The following Supporting Information can be downloaded at: https://www.mdpi.com/article/10.3390/molecules28155858/s1, Figure S1. A plot of the contact angle (θ) of the RL and TX165 aqueous solutions on the PMMA vs. the logarithm of its concentration (logC). Figure S2. A plot of the PTFE–ET interface tension ($\gamma_{\mathrm{SL}}$), film pressure (π = PTFE–water interface tension minus the PTFE–solution one), PTFE–air interface tension calculated from Equation (6) ($\gamma_{\mathrm{SV}}$), and adhesion work of the ET solution to PTFE ($W_{a}$) vs. its concentration ($C_{\mathrm{ET}}$). Figure S3. A plot of the PTFE–solution interface tension ($\gamma_{\mathrm{SL}}$), film pressure (π = PTFE–water interface tension minus the PTFE–solution one), PTFE–air interface tension ($\gamma_{\mathrm{SV}}$) calculated from Equation (6), and adhesion work ($W_{a}$) vs. the logarithm of its concentration (logC). Figure S4. A plot of the solution adhesion work to PMMA ($W_{a}$) calculated from Equation (3) and the Young–Dupre equation, film pressure (π) at the PMMA–air and PMMA–solution interfaces, as well as PMMA–air interface tension calculated from Equation (6) ($\gamma_{\mathrm{SV}}$) for ET vs. its concentration ($C_{\mathrm{ET}}$) (a), as well as for RL (b) and TX165 (c) vs. the logarithm of their concentration (logC). Figure S5. A plot of the Frumkin surface concentration (Γ) at the S–A and PTFE–solution for ET vs. its concentration (a), as well for RL (b) and TX165 (c) vs. the logarithm of their concentration (logC). Figure S6. A plot of the Frumkin surface concentration (Γ) at the S–A, PMMA–solution, and PMMA–air interfaces for ET vs. its concentration (a), as well for RL (b) and TX165 (c) vs. the logarithm of their concentration (logC). Figure S7. A plot of the $\frac{C}{X^{S}}$ at the solution–air, PTFE-S, PMMA–A, and PMMA-S interfaces, as well as $\frac{a^{a}}{X^{S}}$ for the S–A, PTFE-S, PMMA–A, and PMMA-S interfaces for ET vs. its concentration (a), as well for RL (b) and TX165 (c) vs. the logarithm of their concentration (logC). Figure S8. A plot of the standard Gibbs free energy of adsorption ($\Delta G_{\mathrm{ads}}^{0}$) for ET vs. its concentration ($C_{\mathrm{ET}}$) (a), as well as for RL (b) and TX165 (c) vs. the logarithm of their concentration (logC). Figure S9. A plot of the contact angle (θ) of the ET + RL + TX165 mixture aqueous solutions on the PTFE measured (Curves 1 and 2) and calculated from the second-order exponential function, as well as on PMMA measured and calculated form the second-order exponential function vs. the logarithm of the TX165 concentration $\left(\log C_{\mathrm{TX}165}\right)$. Figure S10. A plot of the cosine of the contact angle (cosθ) of the aqueous solution of the ET + RL + TX165 mixture for PTFE (a) and PMMA (b) vs. the reciprocal of the solution surface tension ($\frac{1}{\gamma_{LV}}$). Figure S11. A plot of the ET + RL + TX165 solution and water work of adhesion to PTFE ($W_{a}$) and the PTFE–solution ($\gamma_{\mathrm{SL}}$) and PTFE–air interface tension ($\gamma_{\mathrm{SV}}$) calculated from Equation (6), as well as the film pressure at the PTFE–solution interface ($\pi_{\mathrm{SL}}$) vs. the logarithm of the TX165 concentration $\left(\log C_{\mathrm{TX}165}\right)$ at the constant RL concentration equal to 0.01 mg/dm^3^ and the constant ET concentration equal to 1.07 mol/dm^3^ (a), 3.74 mol/dm^3^ (b), 6.69 mol/dm^3^ (c), and 10.27 mol/dm^3^ (d). Figure S12. A plot of the ET + RL + TX165 solution and water work of adhesion to PTFE ($W_{a}$), PTFE–solution ($\gamma_{\mathrm{SL}}$) and PTFE–air interface tension ($\gamma_{\mathrm{SV}}$) calculated from Equation (6) as well as film pressure at the PTFE–solution interface ($\pi_{\mathrm{SL}}$) vs. the logarithm of the TX165 concentration $\left(\log C_{\mathrm{TX}165}\right)$ at the constant RL concentration equal to 0.5 mg/dm^3^ and the constant ET concentration equal to 1.07 mol/dm^3^ (a), 3.74 mol/dm^3^ (b), 6.69 mol/dm^3^ (c), and 10.27 mol/dm^3^ (d). Figure S13. A plot of the ET + RL + TX165 solution and water work of adhesion to PTFE ($W_{a}$), the PTFE–solution ($\gamma_{SL}$) and PTFE–air interface tension ($\gamma_{SV}$) calculated from Equation (6), as well as the film pressure at the PTFE–solution interface ($\pi_{\mathrm{SL}}$) vs. the logarithm of the TX165 concentration $\left(\log C_{\mathrm{TX}165}\right)$ at the constant RL concentration equal to 5 mg/dm^3^ and the constant ET concentration equal to 1.07 mol/dm^3^ (a), 3.74 mol/dm^3^ (b), 6.69 mol/dm^3^ (c), and 10.27 mol/dm^3^ (d). Figure S14. A plot of the ET + RL + TX165 solution and water work of adhesion to PTFE ($W_{a}$) and the PTFE–solution ($\gamma_{\mathrm{SL}}$) and PTFE–air interface tension ($\gamma_{\mathrm{SV}}$) calculated from Equation (6), as well as film pressure at the PTFE–solution interface ($\pi_{\mathrm{SL}}$) vs. the logarithm of the TX165 concentration $\left(\log C_{\mathrm{TX}165}\right)$ at the constant RL concentration equal to 20 mg/dm^3^ and the constant ET concentration equal to 1.07 mol/dm^3^ (a), 3.74 mol/dm^3^ (b), 6.69 mol/dm^3^ (c), and 10.27 mol/dm^3^ (d). Figure S15. A plot of the ET + RL + TX165 solution work of adhesion to PMMA ($W_{a}$) calculated from the van Oss and the Young–Dupre equation, film pressure at the PMMA–air ($\pi_{\mathrm{SV}}$) and PMMA–solution interface ($\pi_{\mathrm{SL}}$), as well as PMMA–air interface tension ($\gamma_{\mathrm{SV}}$) calculated from Equation (6) vs. the logarithm of the TX165 concentration $\left(\log C_{\mathrm{TX}165}\right)$ at the constant RL concentration equal to 0.01 mg/dm^3^ and the constant ET concentration equal to 1.07 mol/dm^3^ (a), 3.74 mol/dm^3^ (b), 6.69 mol/dm^3^ (c), and 10.27 mol/dm^3^ (d). Figure S16. A plot of the ET + RL + TX165 solution work of adhesion to PMMA ($W_{a}$) calculated from the van Oss and the Young–Dupre equation, film pressure at the PMMA–air ($\pi_{\mathrm{SV}}$) and PMMA–solution interface ($\pi_{\mathrm{SL}}$), as well as PMMA–air interface tension ($\gamma_{\mathrm{SV}}$) calculated from Equation (6) vs. the logarithm of the TX165 concentration $\left(\log C_{\mathrm{TX}165}\right)$ at the constant RL concentration equal to 0.5 mg/dm^3^ and the constant ET concentration equal to 1.07 mol/dm^3^ (a), 3.74 mol/dm^3^ (b), 6.69 mol/dm^3^ (c), and 10.27 mol/dm^3^ (d). Figure S17. A plot of the ET + RL + TX165 solution work of adhesion to PMMA ($W_{a}$) calculated from the van Oss and the Young–Dupre equation, film pressure at the PMMA–air ($\pi_{\mathrm{SV}}$) and PMMA–solution interface ($\pi_{\mathrm{SL}}$), as well as PMMA–air interface tension ($\gamma_{\mathrm{SV}}$) calculated from Equation (6) vs. the logarithm of the TX165 concentration $\left(\log C_{\mathrm{TX}165}\right)$ at the constant RL concentration equal to 5 mg/dm^3^ and the constant ET concentration equal to 1.07 mol/dm^3^ (a), 3.74 mol/dm^3^ (b), 6.69 mol/dm^3^ (c), and 10.27 mol/dm^3^ (d). Figure S18. A plot of the ET + RL + TX165 solution work of adhesion to PMMA ($W_{a}$) calculated from the van Oss and the Young–Dupre equation, film pressure at the PMMA–air ($\pi_{\mathrm{SV}}$) and PMMA–solution interface ($\pi_{\mathrm{SL}}$), as well as PMMA–air interface tension ($\gamma_{\mathrm{SV}}$) calculated from Equation (6) vs. the logarithm of the TX165 concentration $\left(\log C_{\mathrm{TX}165}\right)$ at the constant RL concentration equal to 20 mg/dm^3^ and the constant ET concentration equal to 1.07 mol/dm^3^ (a), 3.74 mol/dm^3^ (b), 6.69 mol/dm^3^ (c), and 10.27 mol/dm^3^ (d). Figure S19. A plot of the surface concentration calculated from the Frumkin equation (Γ) for TX165, RL, and ET at the PTFE–solution interface, as well as their sum at the PTFE–solution and water–air interface (curve 5) vs. the logarithm of the TX165 concentration $\left(\log C_{\mathrm{TX}165}\right)$ at the constant RL concentration equal to 0.01 mg/dm^3^ and the constant ET concentration equal to 1.07 mol/dm^3^ (a), 3.74 mol/dm^3^ (b), 6.69 mol/dm^3^ (c), and 10.27 mol/dm^3^ (d). Figure S20. A plot of the surface concentration calculated from the Frumkin equation (Γ) for TX165, RL, and ET at the PTFE–solution interface and their sum at this interface, as well as their sum at the water–air interface vs. the logarithm of the TX165 concentration $\left(\log C_{\mathrm{TX}165}\right)$ at the constant RL concentration equal to 0.5 mg/dm^3^ and the constant ET concentration equal to 1.07 mol/dm^3^ (a), 3.74 mol/dm^3^ (b), 6.69 mol/dm^3^ (c), and 10.27 mol/dm^3^ (d). Figure S21. A plot of the surface concentration calculated from the Frumkin equation (Γ) for TX165, RL, and ET at the PTFE–solution interface and their sum at this interface, as well as their sum at the water–air interface vs. the logarithm of the TX165 concentration $\left(\log C_{\mathrm{TX}165}\right)$ at the constant RL concentration equal to 5 mg/dm^3^ and the constant ET concentration equal to 1.07 mol/dm^3^ (a), 3.74 mol/dm^3^ (b), 6.69 mol/dm^3^ (c), and 10.27 mol/dm^3^ (d). Figure S22. A plot of the surface concentration calculated from the Frumkin equation (Γ) for TX165, RL, and ET at the PTFE–solution interface and their sum at this interface, as well as their sum at the water–air interface vs. the logarithm of the TX165 concentration $\left(\log C_{\mathrm{TX}165}\right)$ at the constant RL concentration equal to 20 mg/dm^3^ and the constant ET concentration equal to 1.07 mol/dm^3^ (a), 3.74 mol/dm^3^ (b), 6.69 mol/dm^3^ (c), and 10.27 mol/dm^3^ (d). Figure S23. A plot of the surface concentration calculated from the Frumkin equation (Γ) for TX165, RL, ET, and their sum, as well as their sum at the water–air interface vs. the logarithm of the TX165 concentration $\left(\log C_{\mathrm{TX}165}\right)$ at the constant RL concentration equal to 0.01 mg/dm^3^ and the constant ET concentration equal to 1.07 mol/dm^3^ (a), 3.74 mol/dm^3^ (b), 6.69 mol/dm^3^ (c), and 10.27 mol/dm^3^ (d). Figure S24. A plot of the surface concentration calculated from the Frumkin equation (Γ) for TX165, RL, ET, and their sum, as well as their sum at the water–air interface vs. the logarithm of the TX165 concentration $\left(\log C_{\mathrm{TX}165}\right)$ at the constant RL concentration equal to 0.5 mg/dm^3^ and the constant ET concentration equal to 1.07 mol/dm^3^ (a), 3.74 mol/dm^3^ (b), 6.69 mol/dm^3^ (c), and 10.27 mol/dm^3^ (d). Figure S25. A plot of the surface concentration calculated from the Frumkin equation (Γ) for TX165, RL, ET, and their sum, as well as their sum at the water–air interface vs. the logarithm of the TX165 concentration $\left(\log C_{\mathrm{TX}165}\right)$ at the constant RL concentration equal to 5 mg/dm^3^ and the constant ET concentration equal to 1.07 mol/dm^3^ (a), 3.74 mol/dm^3^ (b), 6.69 mol/dm^3^ (c), and 10.27 mol/dm^3^ (d). Figure S26. A plot of the surface concentration calculated from the Frumkin equation (Γ) for TX165, RL, ET, and their sum, as well as their sum at the water–air interface vs. the logarithm of the TX165 concentration $\left(\log C_{\mathrm{TX}165}\right)$ at the constant RL concentration equal to 20 mg/dm^3^ and the constant ET concentration equal to 1.07 mol/dm^3^ (a), 3.74 mol/dm^3^ (b), 6.69 mol/dm^3^ (c), and 10.27 mol/dm^3^ (d). Figure S27. A plot of the $\frac{C}{X^{S}}$ for ET (a) RL (b,c) and TX165 (d,e) vs. the logarithm of the TX165 concentration $\left(\log C_{\mathrm{TX}165}\right)$. Figure S28. A plot of the $\frac{C}{X^{S}}$ at the solution–air interface, as well as $\frac{a^{a}}{X^{S}}$ at the PMMA–air (a,b) and PMMA–solution interfaces (c) for ET vs. the logarithm of the TX165 concentration $\left(\log C_{\mathrm{TX}165}\right)$. Figure S29. A plot of the $\frac{C}{X^{S}}$ at the solution–air interface (Curves 1–16) and PMMA–air interface calculated for RL at its area equal to 69.09 Å^2^ (Curves 1′–16′) and 87.3 Å^2^ vs. the logarithm of the TX165 concentration $\left(\log C_{\mathrm{TX}165}\right)$. Figure S30. A plot of the $\frac{C}{X^{S}}$ at the PMMA–solution interface calculated for RL at its area equal to 69.09 Å^2^ and 87.3 Å^2^ vs. the logarithm of the TX165 concentration $\left(\log C_{\mathrm{TX}165}\right)$. Figure S31. A plot of the $\frac{C}{X^{S}}$ at the solution–air interface and PMMA–air interface calculated for TX165 at its area equal to 35.7 Å^2^ and 52.12 Å^2^ vs. the logarithm of the TX165 concentration $\left(\log C_{\mathrm{TX}165}\right)$. Figure S32. A plot of the $\frac{C}{X^{S}}$ at the PMMA–solution interface calculated for TX165 at its area equal to 35.7Å^2^ and 52.12 Å^2^ vs. the logarithm of the TX165 concentration $\left(\log C_{\mathrm{TX}165}\right)$. Figure S33. A plot of the standard Gibbs free energy of adsorption ($\Delta G_{\mathrm{ads}}^{0}$) at the PTFE–solution for ET (a) for RL (b,c) and TX165 (d,e) vs. the logarithm of the TX165 concentration $\left(\log C_{\mathrm{TX}165}\right)$. Figure S34. A plot of the standard Gibbs free energy of adsorption ($\Delta G_{\mathrm{ads}}^{0}$) for ET at the PMMA–air (a) and PMMA–solution (b) vs. the logarithm the TX165 concentration $\left(\log C_{\mathrm{TX}165}\right)$. Figure S35. A plot of the standard Gibbs free energy of adsorption ($\Delta G_{\mathrm{ads}}^{0}$) for RL at the PMMA–air (a,b) and PMMA–solution (c,d) vs. the logarithm of the TX165 concentration $\left(\log C_{\mathrm{TX}165}\right)$. Figure S36. A plot of the standard Gibbs free energy of adsorption ($\Delta G_{\mathrm{ads}}^{0}$) for TX165 at the PMMA–air (a,b) and PMMA–solution (c,d) vs. the logarithm of the TX165 concentration $\left(\log C_{\mathrm{TX}165}\right)$.

## Sample Availability

Samples of the compounds are not available from the authors.

## References

[1] Rosen M.J. (2004). Surfactants and Interfacial Phenomena.

[2] Zdziennicka A., Jańczuk B. (2020). Modification of adsorption, aggregation and wetting properties of surfactants by short chain alcohols. Adv. Colloid Interface Sci..

[3] Szymczyk K., Zdziennicka A., Jańczuk B. (2021). Properties of some nonionic fluorocarbon surfactants and their mixtures with hydrocarbon ones. Adv. Colloid Interface Sci..

[4] van Oss C.J. (1994). Interfacial Forces in Aqueous Media.

[5] Schultz J., Nardin M. (1992). Modern Approaches to Wettability: Theory and Applications.

[6] Young T. (1805). An essay on the cohesion of fluids. Philos. Trans. R. Soc..

[7] Adamson W., Gast A.P. (1997). Physical Chemistry of Surfaces.

[8] Adam N.K. (1957). Use of the term ‘Young’s equation for contact angle. Nature.

[9] Blake T.D., Tadros T.F. (1984). Wetting. Surfactants.

[10] Owens N.F., Richmond P., Gregory D., Mingins J., Chan D., Padday J.F. (1978). Contact angles of pure liquids and surfactants on low-energy surfaces. Wetting, Spreading and Adhesion.

[11] Wu S. (1979). Surface tension of solids: An equation of state analysis. J. Colloid Interface Sci..

[12] Fowkes F.M. (1964). Attractive forces at interfaces. Ind. Eng. Chem..

[13] Neumann A.W., Good R.J., Hope C.J., Sejpal M. (1974). An equation-of-state approach to determine surface tensions of low-energy solids from contact angles. J. Colloid Interface Sci..

[14] Kwok D.Y., Neumann A.W. (1999). Contact angle measurement and contact angle interpretation. Adv. Colloid Interface Sci..

[15] Fowkes F.M., McCarthy D.C., Mostafa A. (1980). Contact angles and the equilibrium spreading pressures of liquids on hydrophobic solids. J. Colloid Interface Sci..

[16] Good R.J. (1992). Contact angle, wetting, and adhesion: A critical review. J. Adhes. Sci. Technol..

[17] van Oss C.J., Constanzo P.M. (1992). Adhesion of anionic surfactants to polymer surfaces and low-energy materials. J. Adhes. Sci. Technol..

[18] Zhang Y., Placek T.L., Jahan R., Alexandridis P., Tsianou M. (2022). Rhamnolipid Micellization and Adsorption Properties. Int. J. Mol. Sci..

[19] Ikizler B., Arslan G., Kipcak E., Dirik C., Celenk D., Aktuglu T., Helvacı S.S., Peker S. (2017). Surface adsorption and spontaneous aggregation of rhamnolipid mixtures in aqueous solutions. Colloids Surf. A Physicochem. Eng. Asp..

[20] Mendes A.N., Filgueiras L.A., Pinto J.C., Nele M. (2015). Physicochemical Properties of Rhamnolipid Biosurfactant from Pseudomonas aeruginosa PA1 to Applications in Microemulsions. J. Biomater. Nanobiotechnol..

[21] Abalos A., Pinazo A., Casals M.R., García F., Manresa A. (2001). Physicochemical and antimicrobial properties of new rhamnolipids produced by Pseudomonas aeruginosa AT10 from soybean oil refinery wastes. Langmuir.

[22] Semkova S., Antov G., Illiev I., Tsoneva I., Lefterov P., Christova N., Nacheva L., Stoineva I., Kabaivanova L., Staneva G. (2021). Rhamnolipid Biosurfactants—Possible Natural Anticancer Agents and Autophagy Inhibitors. Separations.

[23] Ammar A., Lagenaur C., Jannetta P. (1990). Neural tissue compatibility of Teflon as an implant material for microvascular decompression, Neurosurg. Rev..

[24] Michael S., Godin M.D., Della Thore T.M.D. (2008). The use of expanded polytetrafluoroethylene (e-PTFE) implants in rhinoplasty. Oper. Tech. Otolaryngol..

[25] Leigh J.A. (1975). Use of PMMA in expansion dental implants. J. Biomed. Mater. Res..

[26] van Oss C.J., Good R.J. (1989). Surface tension and the solubility of polymers and biopolymers: The role of polar and apolar interfacial free energies. J. Macromol. Sci. Chem..

[27] van Oss C.J., Chaudhury M.K., Good R.J. (1987). Monopolar surfaces. Adv. Colloid Interface Sci..

[28] Neumann A.W. (1974). Contact angles and their temperature dependence. Thermodynamic status, measurements, interpretation and application. Adv. Colloid Interface Sci..

[29] Jańczuk B., Wójcik W., Zdziennicka A. (1993). determination of the components of the surface tension of some liquids from interfacial liquid-liquid tension measurements. J. Colloid Interface Sci..

[30] Zdziennicka A., Krawczyk J., Szymczyk K., Jańczuk B. (2017). Components and parameters of liquids and some polymers surface tension at different temperature. Coll. Surf. A.

[31] Della Volpe C., Siboni S., Mittal K.L. (2000). Acid–Base Interactions: Relevance to Adhesion Science and Technology.

[32] Mańko D., Zdziennicka A., Jańczuk B. (2014). Thermodynamic properties of rhamnolipid micellization and adsorption. Colloids Surf. B Biointerfaces.

[33] Chodzińska A., Zdziennicka A., Jańczuk B. (2012). Volumetric and surface properties of short chain alcohols in aqueous solution–air systems at 293 K. J. Sol. Chem..

[34] Grzywaczyk A., Smułek W., Kaczorek E., Zdziennicka A., Jańczuk B. (2023). Thermodynamic consideration of the solid saponin extract drop-air system. Molecules.

[35] Lucassen-Reynders E.H. (1963). Contact angles and adsorption on solids. J. Phys. Chem..

[36] Rekiel E., Zdziennicka A., Szymczyk K., Jańczuk B. (2022). Thermodynamic analysis of the adsorption and micellization activity of the mixtures of rhamnolipid and surfactin with Triton X-165. Molecules.

[37] Vogel A.I. (2006). Preparatyka Organiczna, WYD. 3.

