# Supplementary material for: Wetting Behaviour of Water, Ethanol, Rhamnolipid, and Triton X-165 Mixture in the Polymer–Solution Drop–Air System
Source: Molecules. 2023 Aug 3;28(15):5858. doi: 10.3390/molecules28155858 (PMC10421433; doi:10.3390/molecules28155858)
Supplement: Supplementary file 1 [file molecules-28-05858-s001.zip › molecules-2505078-supplementary.pdf]

Supplementary Material

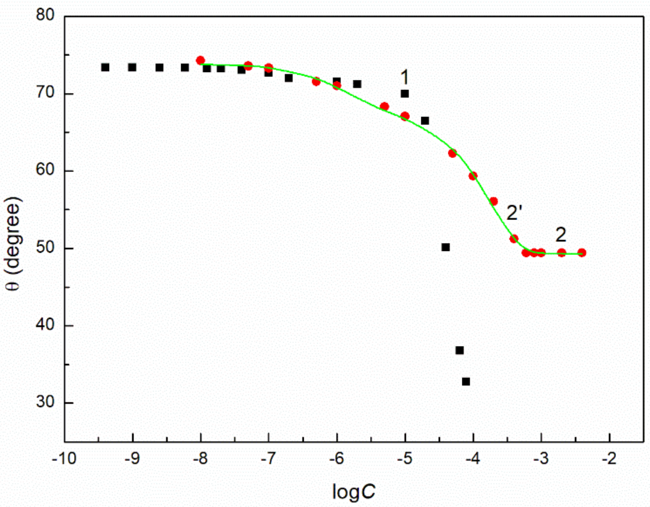

Figure S1. A plot of the contact angle (θ) of the RL (points1) and TX165 (points 2 and curve 2′) aqueous solutions on the PMMA vs. the logarithm of its concentration ($\log C$). Points 1 and 2 correspond the measured values, curve 2′ corresponds to the values calculated from the exponential function of the second order.

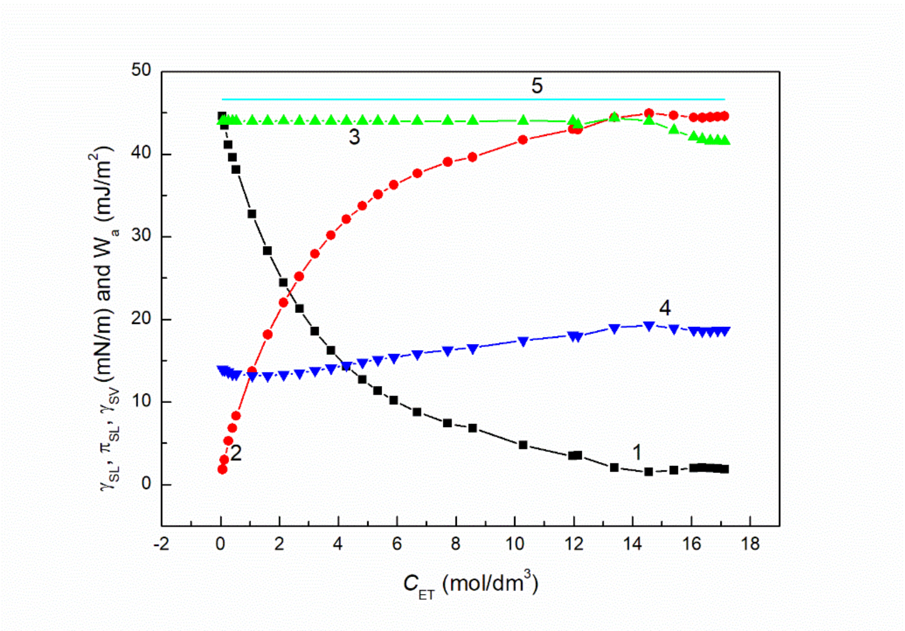

Figure S2. A plot of the PTFE-ET interface tension (γ_{SL}) (curve 1), film pressure (π = PTFE-water interface tension minus PTFE-solution one) (curve 2), PTFE-air interface tension calculated from Equation (6) (γ_{SV}) (curve 4) and adhesion work of ET solution to PTFE (W_a) (curve 3) vs. its concentration (C_{ET}). Curve 5 corresponds to the adhesion work of water to PTFE.

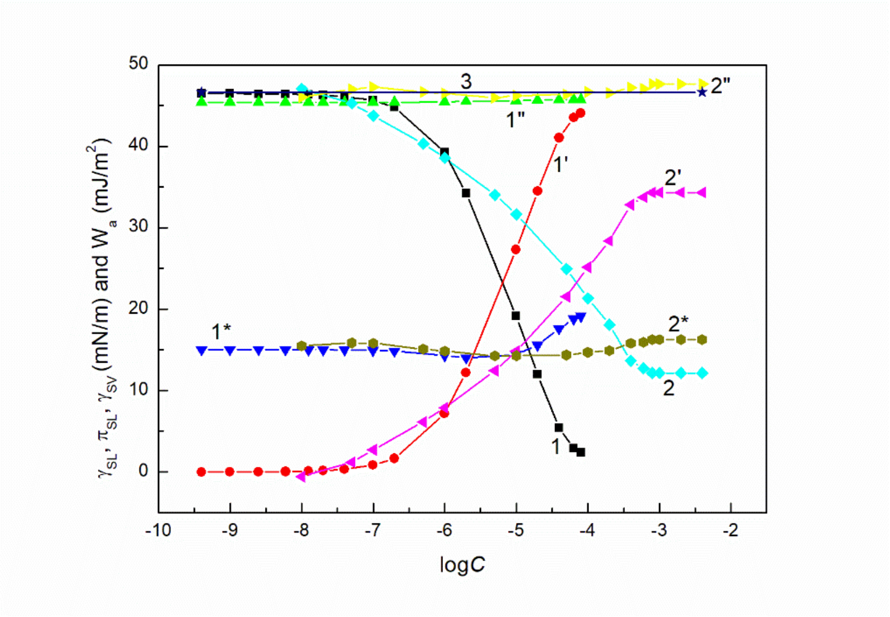

Figure S3. A plot of the PTFE-solution interface tension (γ_{SL}) (curves 1 and 2), film pressure (π = PTFE-water interface tension minus PTFE-solution one) (curves 1′ and 2′), PTFE-air interface tension (γ_{SV}) calculated from Eq. (6) (curves 1″ and 2″) and adhesion work (W_a) (curves 1* and 2*) vs. the logarithm of its concentration (log$C$). Curve 3 corresponds to the adhesion work of water to PTFE. Curves 1 – 1* correspond to the RL aqueous solution, curves 2 – 2* correspond to the TX165 aqueous solution.

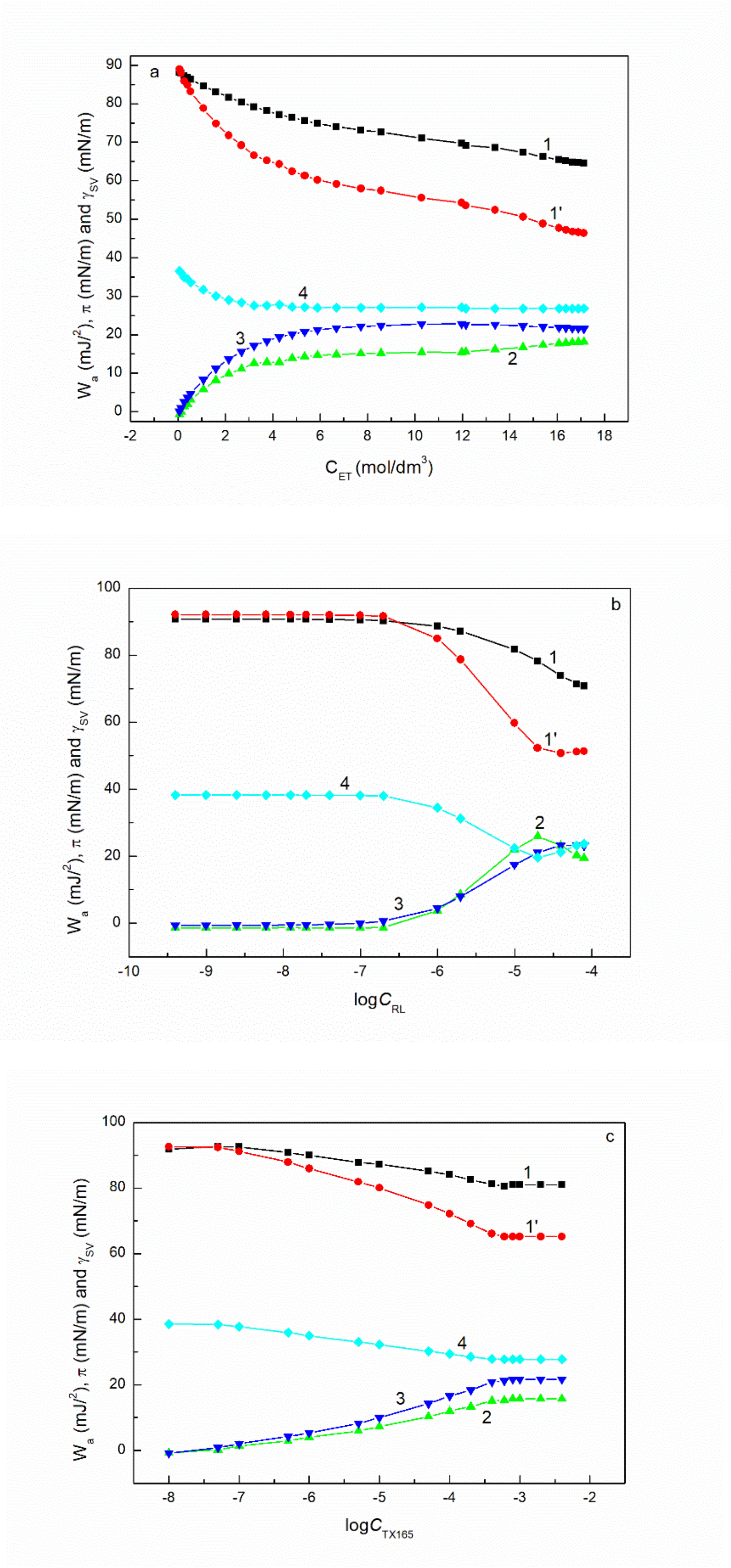

Figure S4. A plot of the solution adhesion work to PMMA (W_a) calculated from Equation (3) (curve 1) and Young-Dupre equation (curve 1′), film pressure (π) at the PMMA-air (curve 2) and PMMA-solution (curve 3) interfaces aa well as PMMA-air interface tension calculated from Eq. (6) (γ_{SV}) (curve 4) for ET vs. its concentration (C_{ET}) (a) as well as for RL (b) and TX165 (c) vs. the logarithm of their concentration (log$C$).

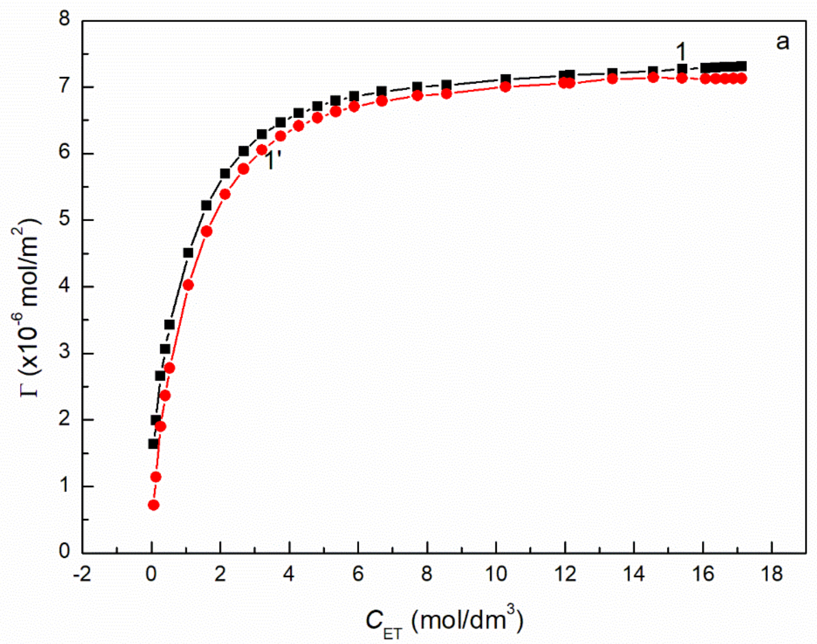

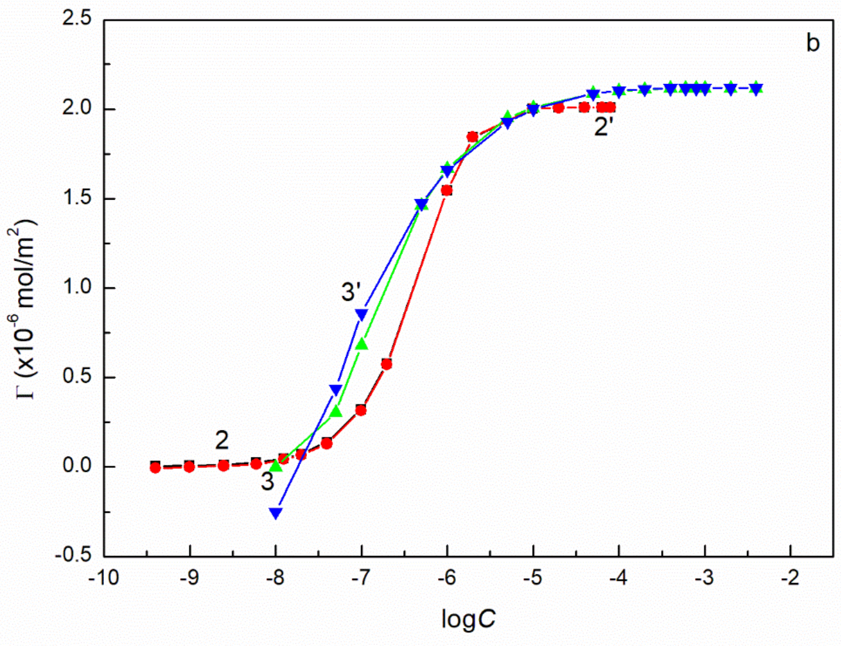

Figure S5. A plot of the Frumkin surface concentration (Γ) at the S-A (cures 1 – 3) and PTFE-solution (curves 1′ – 3′) for ET (curves 1 and 1′) vs. its concentration (a) as well for RL (curves 2 and 2′) (b) and TX165 (curves 3 and 3′) (c) vs. the logarithm of their concentration (log$C$).

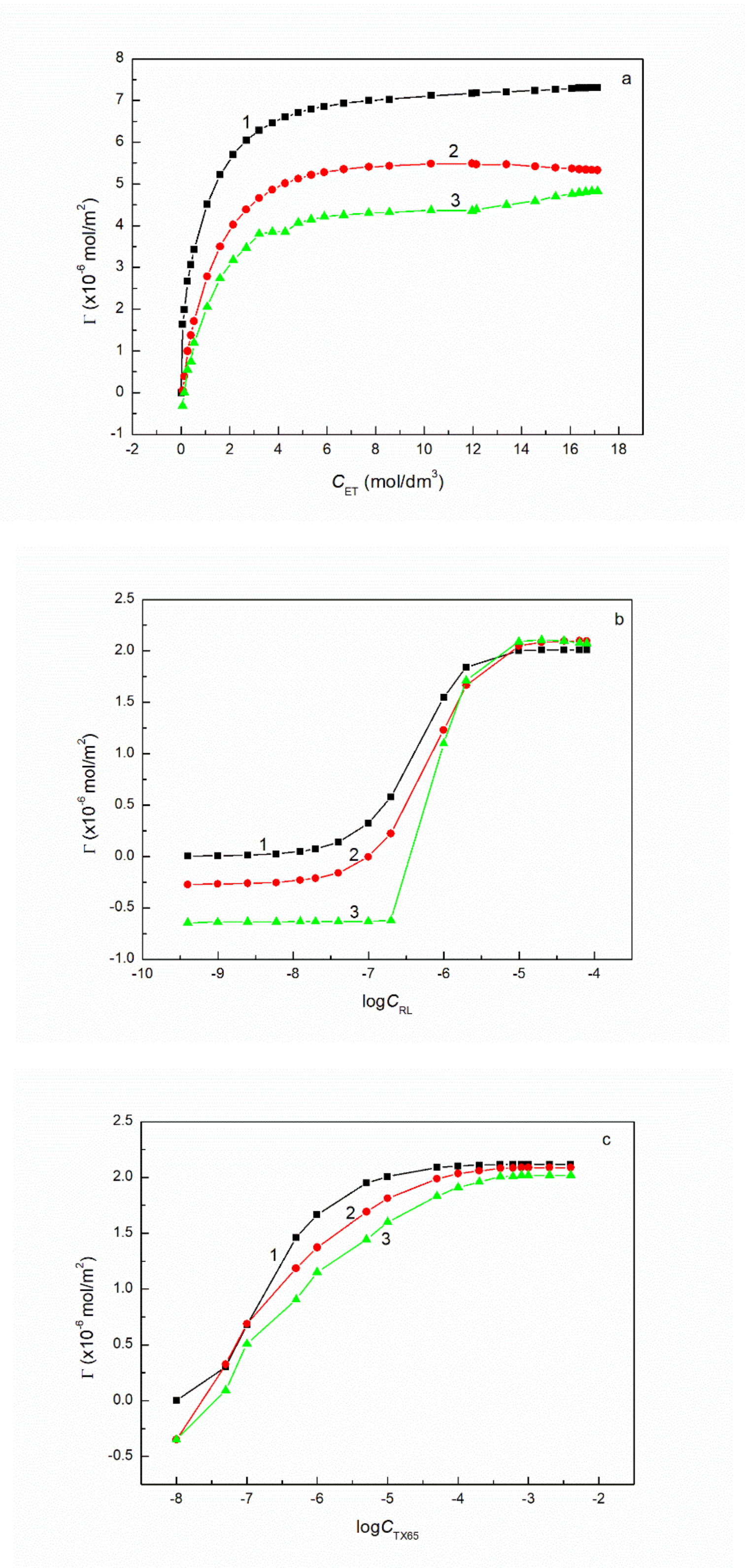

Figure S6. A plot of the Frumkin surface concentration (Γ) at the S-A (cure 1), PMMA-solution (curve 2) and PMMA-air (curve 3) for ET vs. its concentration (a), as well for RL (b) and TX165 (c) vs. the logarithm of their concentration (log*C*).

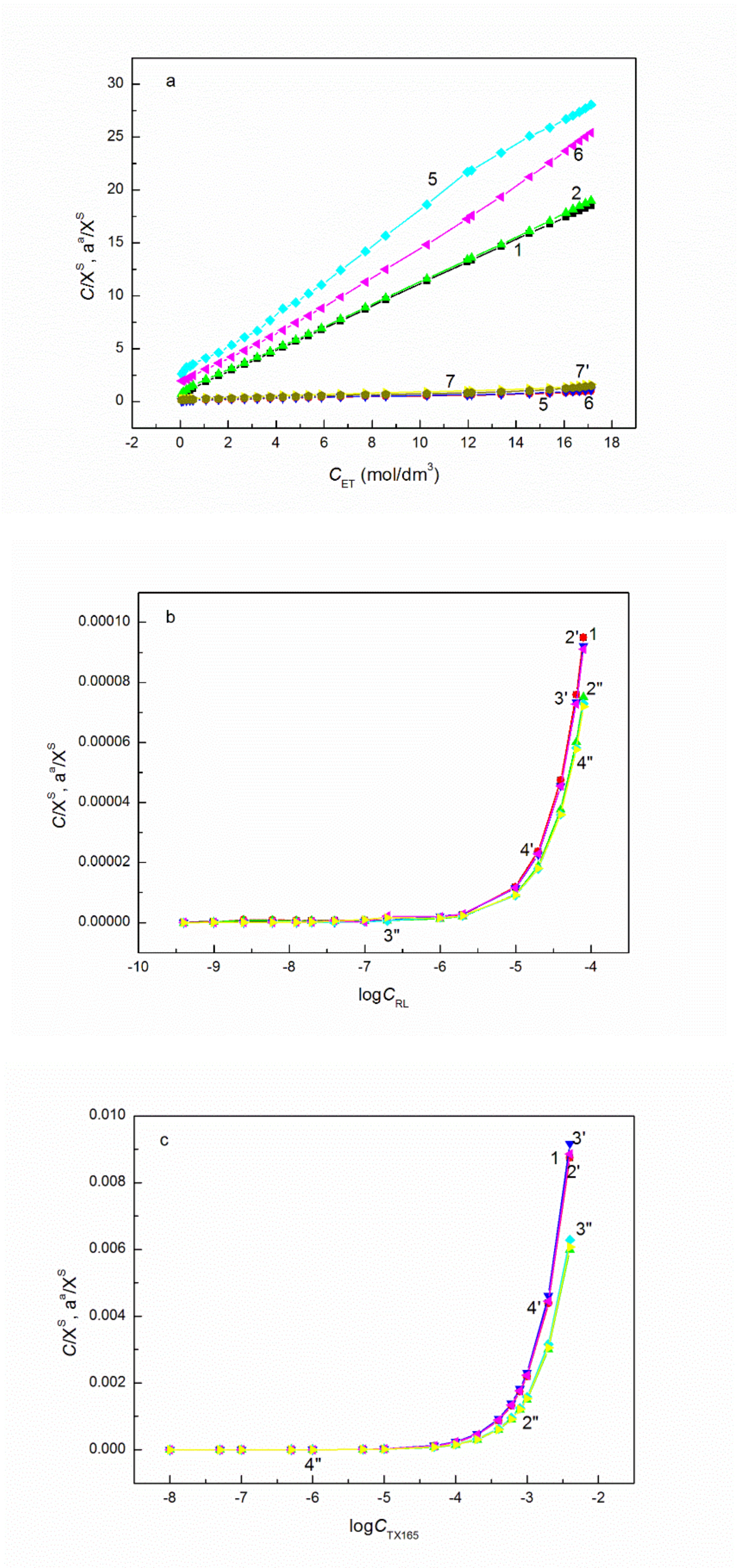

Figure S7. A plot of the $\frac{C}{X^S}$ at the solution-air (curve 1), PTFE-S (curves 2, 2′ and 2″), PMMA-A (curves 3, 3′ and 3″) and PMMA-S (curves 4, 4′ and 4″) as well as $\frac{a^a}{X^S}$ for S-A (curve 5), PTFE-S (curve 6), PMMA-A (curve 7) and PMMA-S (curve 7′) for ET vs. its concentration (a), as well for RL (b) and TX165 (c) vs. the logarithm of their concentration (log$C$). Curves 2′, 3′ and 4′ correspond to the RL area equal to 69.09 Å^2 and for TX165 35.7 Å^2, curves 2″, 3″ and 4″ correspond to the RL area equal to 87.3 Å^2 and for TX165 52.12 Å^2.

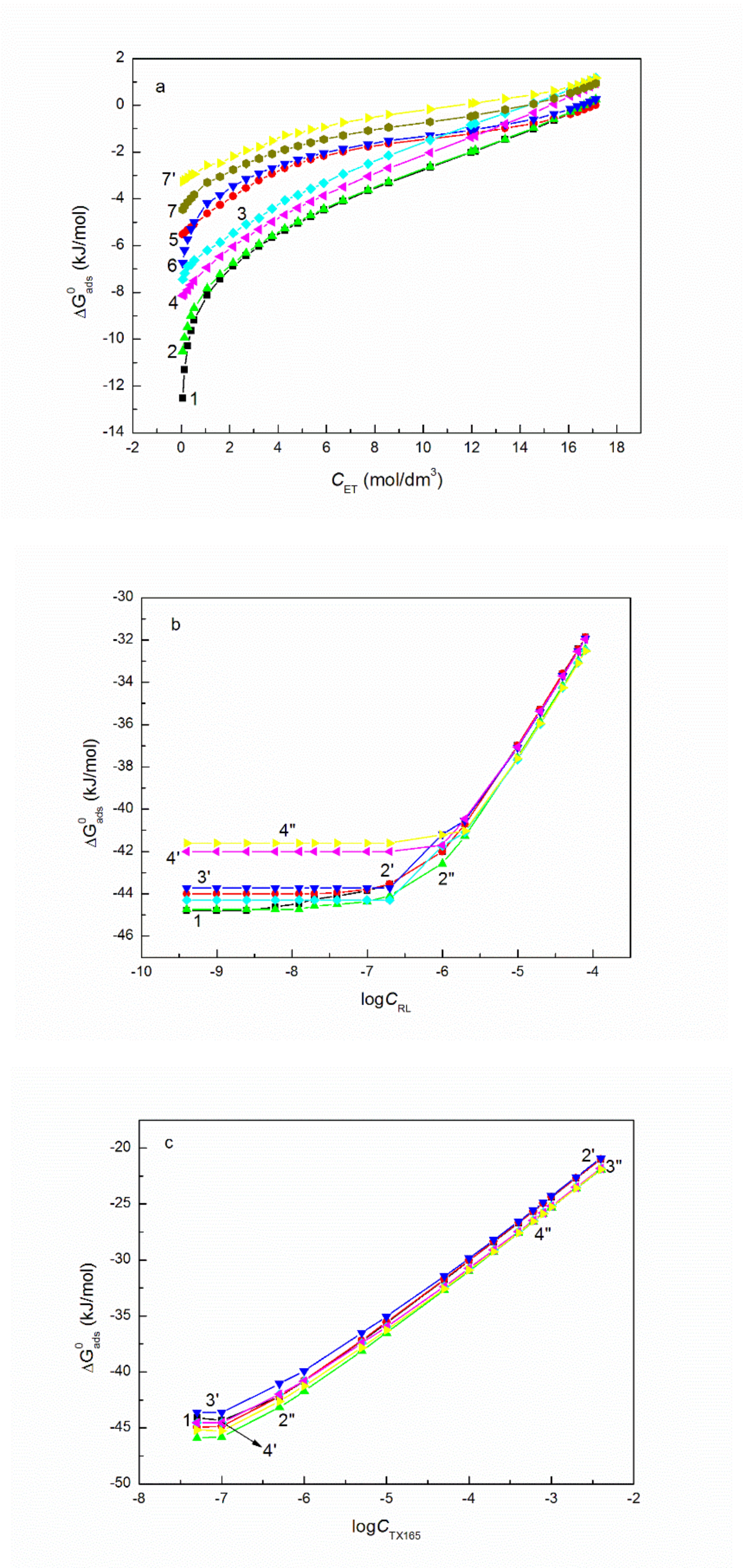

Figure S8. A plot of the standard Gibbs free energy of adsorption (ΔG^0_{ads}) for ET vs. its concentration (C_{ET}) (a) as well as for RL (b) and TX165 (c) vs. the logarithm of their concentration (log$C$). The values of ΔG^0_{ads} obtained based on the data presented in Figure S7. The designation of the curves corresponds to their designation in Figure 7.

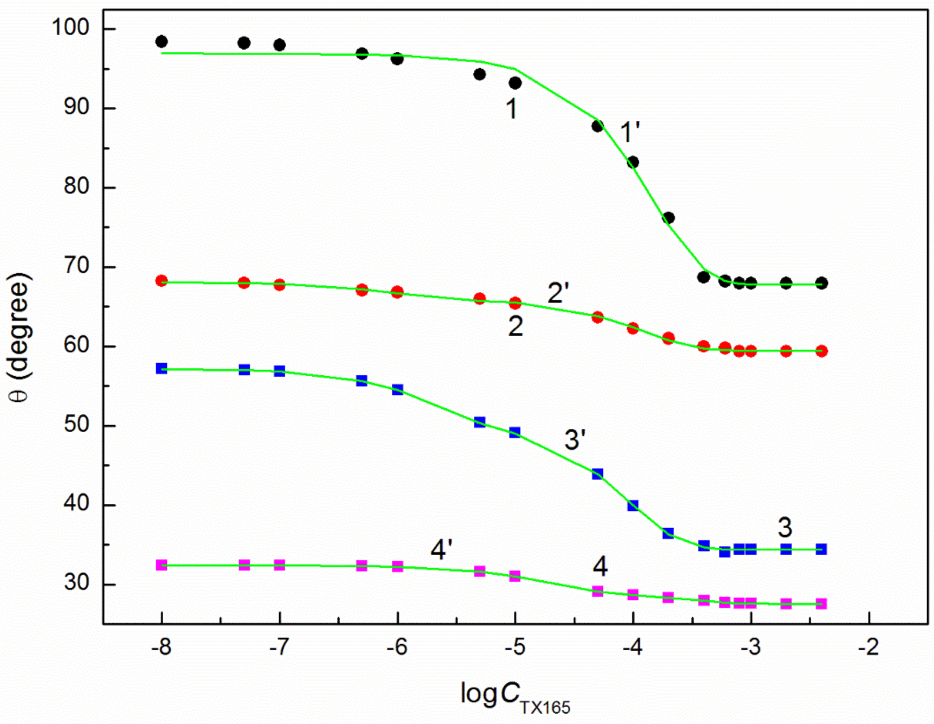

Figure S9. A plot of the contact angle (θ) of the ET + RL + TX165 mixture aqueous solutions on the PTFE measured (curves 1 and 2) and calculated from the exponential function of the second order (curves 1′ and 2′) as well as on PMMA measured (cures 3 and 4) and calculated form the exponential function of the second order vs. the logarithm of TX165 concentration ($\log C_{TX165}$). Curves 1, 1′, 3 and 3′correspond to the constant RL concentration equal to 0.01 mg/dm^3 and ET 6.69 mol/dm^3, curves 2, 2′, 4 and 4′correspond to the constant RL concentration equal to 5 mg/dm^3 and ET 1.07 mol/dm^3, respectively.

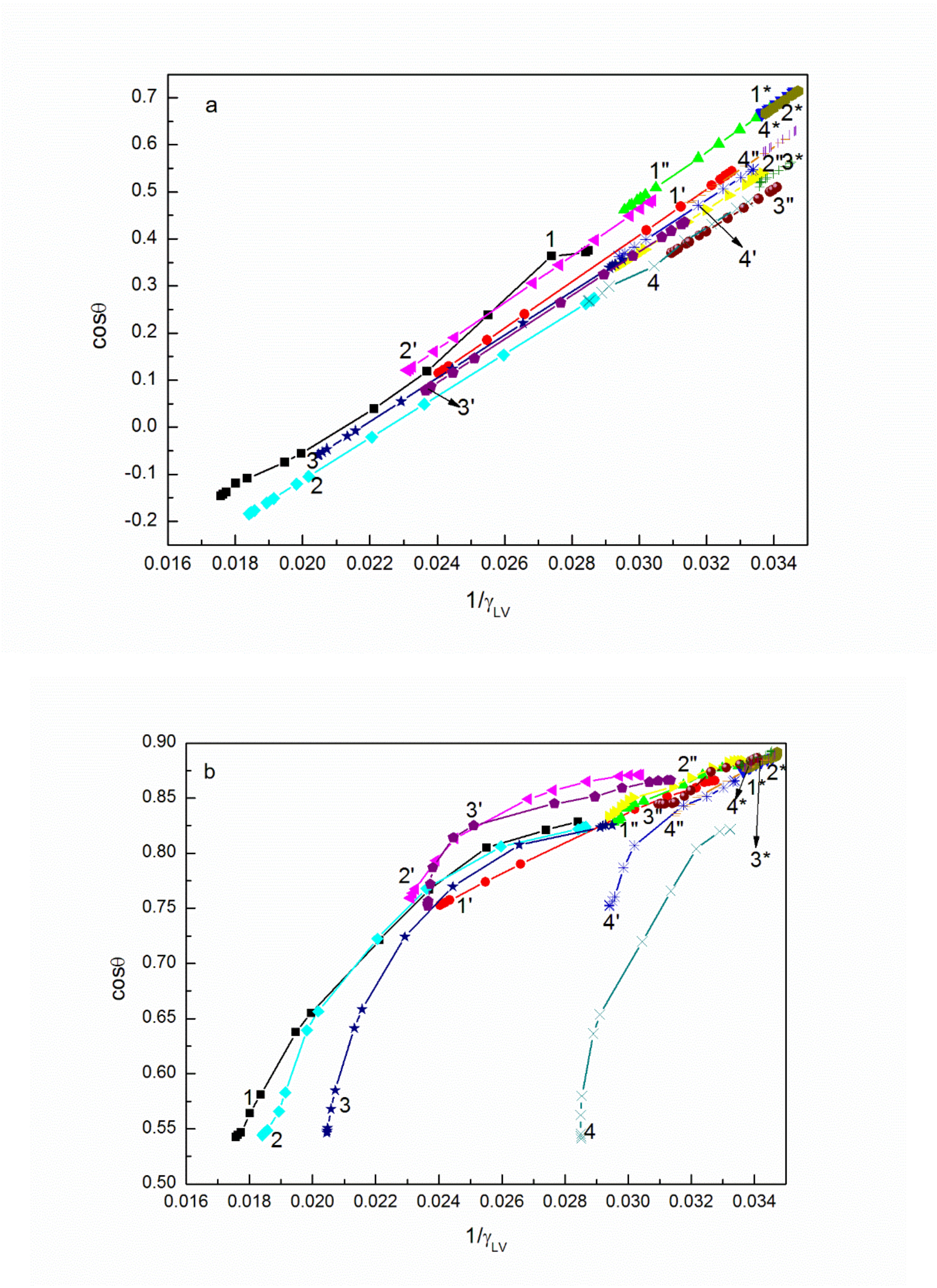

Figure S10. A plot of the cosine of the contact angle ($\cos\theta$) of the aqueous solution of the ET + RL + TX165 mixture for PTFE (a) and PMMA (b) vs. the reciprocal of the solution surface tension ($\frac{1}{\gamma_{LV}}$). Curves 1 – 1*, 2 – 2*, 3 – 3* and 4 – 4* correspond to the constant RL concentration equal to 0.01, 0.5, 5 and 20 mg/dm^3, respectively. Curves 1 – 4, 1′ – 4′, 1″ – 4″ and 1* - 4* correspond to the constant ET concentration equal to 1.07, 3.74, 6.69 and 10.27 mol/dm^3, respectively.

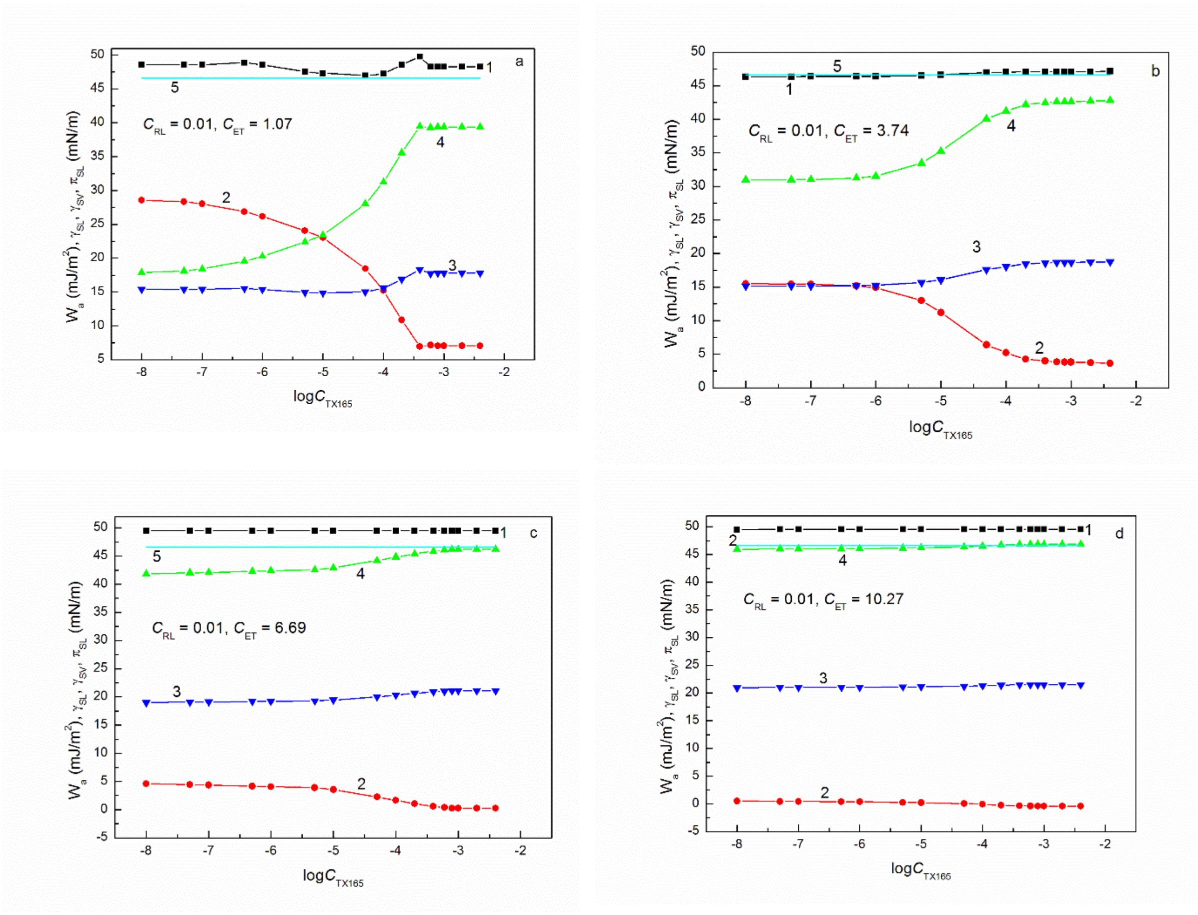

Figure S11. A plot of the ET + RL + TX165 solution (curve 1) and water (curve 5) work of adhesion to PTFE (W_a), PTFE-solution (γ_{SL}) (curve 2) and PTFE-air interface tension (γ_{SV}) (curve 3) calculated from Equation (6) as well as film pressure at the PTFE-solution interface (π_{SL}) (curve 4) vs. the logarithm of TX165 concentration ($\log C_{\mathrm{TX165}}$) at the constant RL concentration equal to 0.01 mg/dm^3 and the constant ET concentration equal to 1.07 mol/dm^3 (a), 3.74 mol/dm^3 (b), 6.69 mol/dm^3 (c) and 10.27 mol/dm^3 (d).

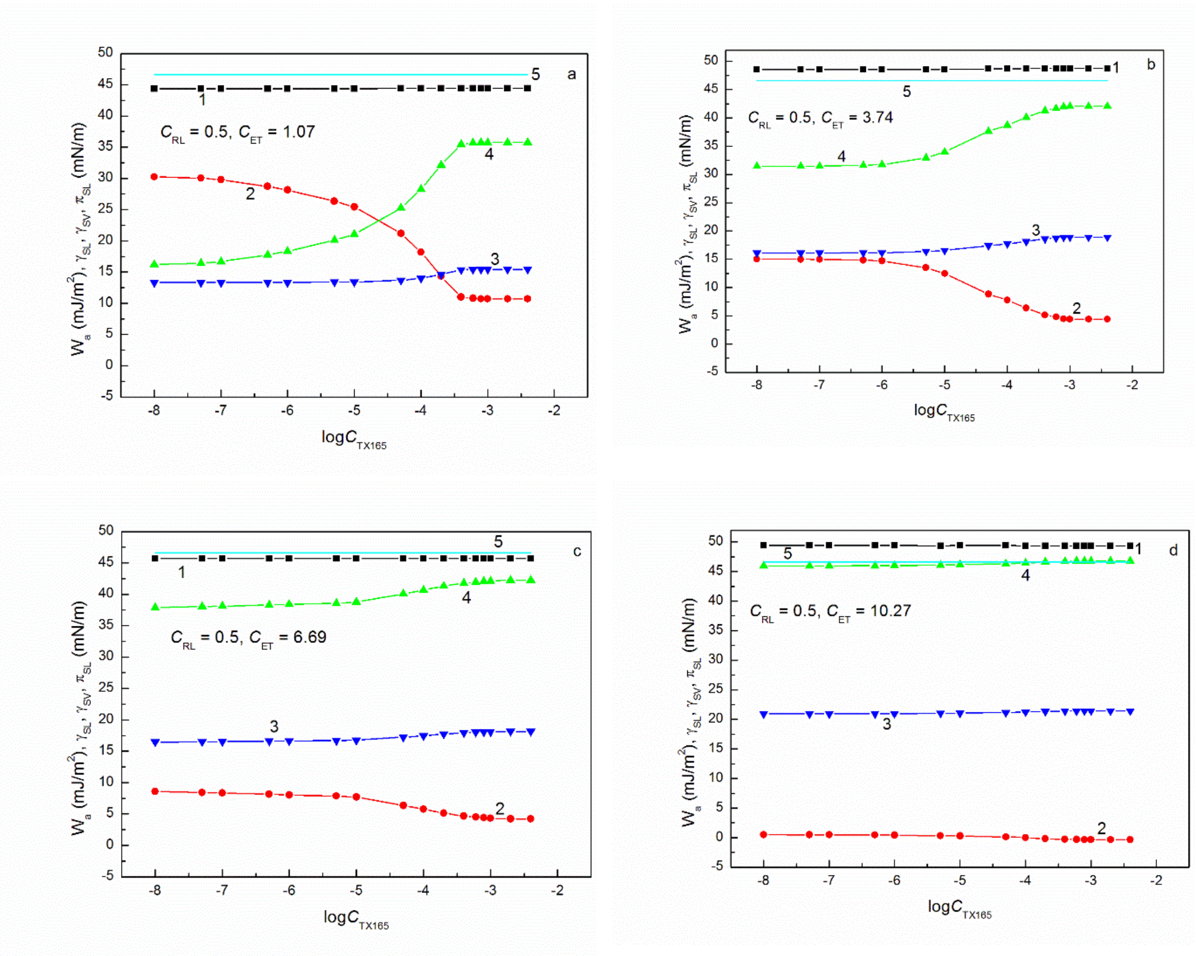

Figure S12. A plot of the ET + RL + TX165 solution (curve 1) and water (curve 5) work of adhesion to PTFE (W_a), PTFE-solution (γ_{SL}) (curve 2) and PTFE-air interface tension (γ_{SV}) (curve 3) calculated from Equation (6) as well as film pressure at the PTFE-solution interface (π_{SL}) (curve 4) vs. the logarithm of TX165 concentration (logC_{TX165}) at the constant RL concentration equal to 0.5 mg/dm^3 and the constant ET concentration equal to 1.07 mol/dm^3 (a), 3.74 mol/dm^3 (b), 6.69 mol/dm^3 (c) and 10.27 mol/dm^3 (d).

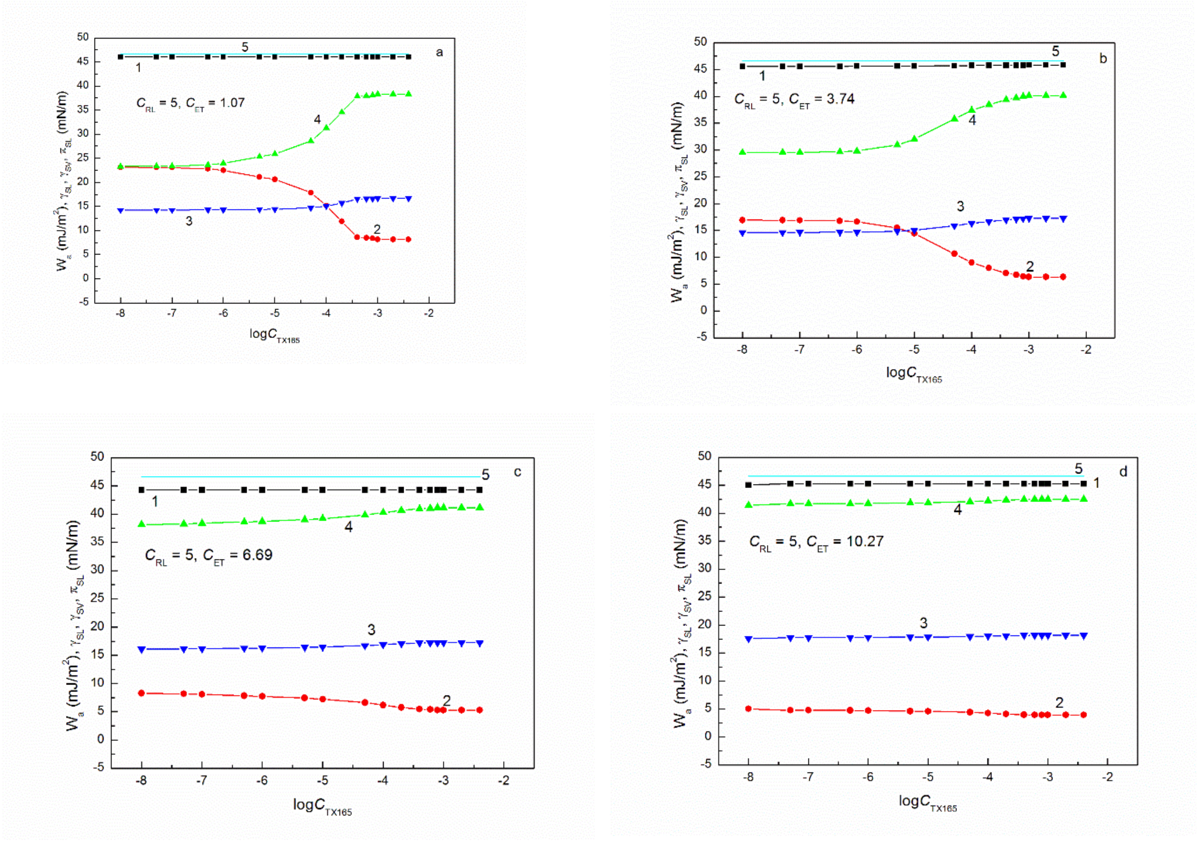

Figure S13. A plot of the ET + RL + TX165 solution (curve 1) and water (curve 5) work of adhesion to PTFE (W_a), PTFE-solution (γ_{SL}) (curve 2) and PTFE-air interface tension (γ_{SV}) (curve 3) calculated from Equation (6) as well as film pressure at the PTFE-solution interface (π_{SL}) (curve 4) vs. the logarithm of TX165 concentration ($\log C_{\mathrm{TX165}}$) at the constant RL concentration equal to 5 mg/dm^3 and the constant ET concentration equal to 1.07 mol/dm^3 (a), 3.74 mol/dm^3 (b), 6.69 mol/dm^3 (c) and 10.27 mol/dm^3 (d).

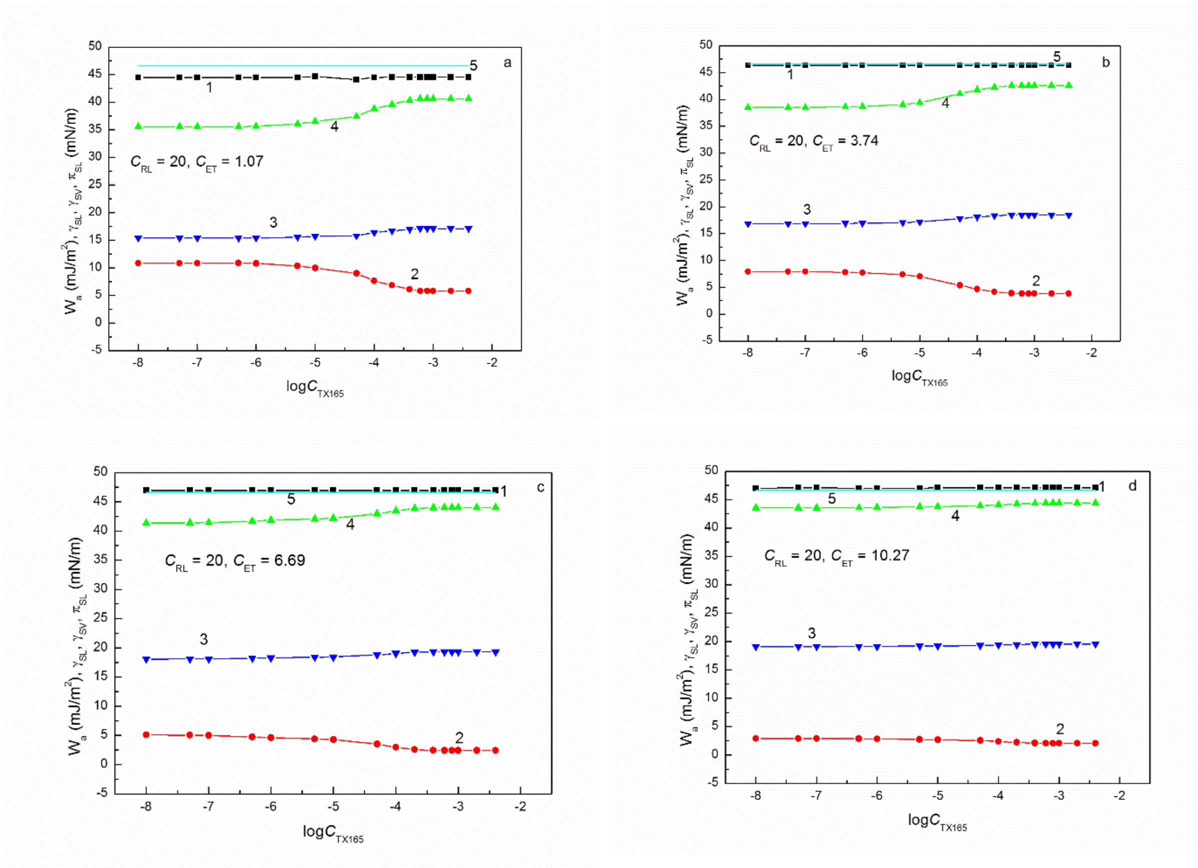

Figure S14. A plot of the ET + RL + TX165 solution (curve 1) and water (curve 5) work of adhesion to PTFE (W_a), PTFE-solution (γ_{SL}) (curve 2) and PTFE-air interface tension (γ_{SV}) (curve 3) calculated from Equation (6) as well as film pressure at the PTFE-solution interface (π_{SL}) (curve 4) vs. the logarithm of TX165 concentration (logC_{TX165}) at the constant RL concentration equal to 20 mg/dm^3 and the constant ET concentration equal to 1.07 mol/dm^3 (a), 3.74 mol/dm^3 (b), 6.69 mol/dm^3 (c) and 10.27 mol/dm^3 (d).

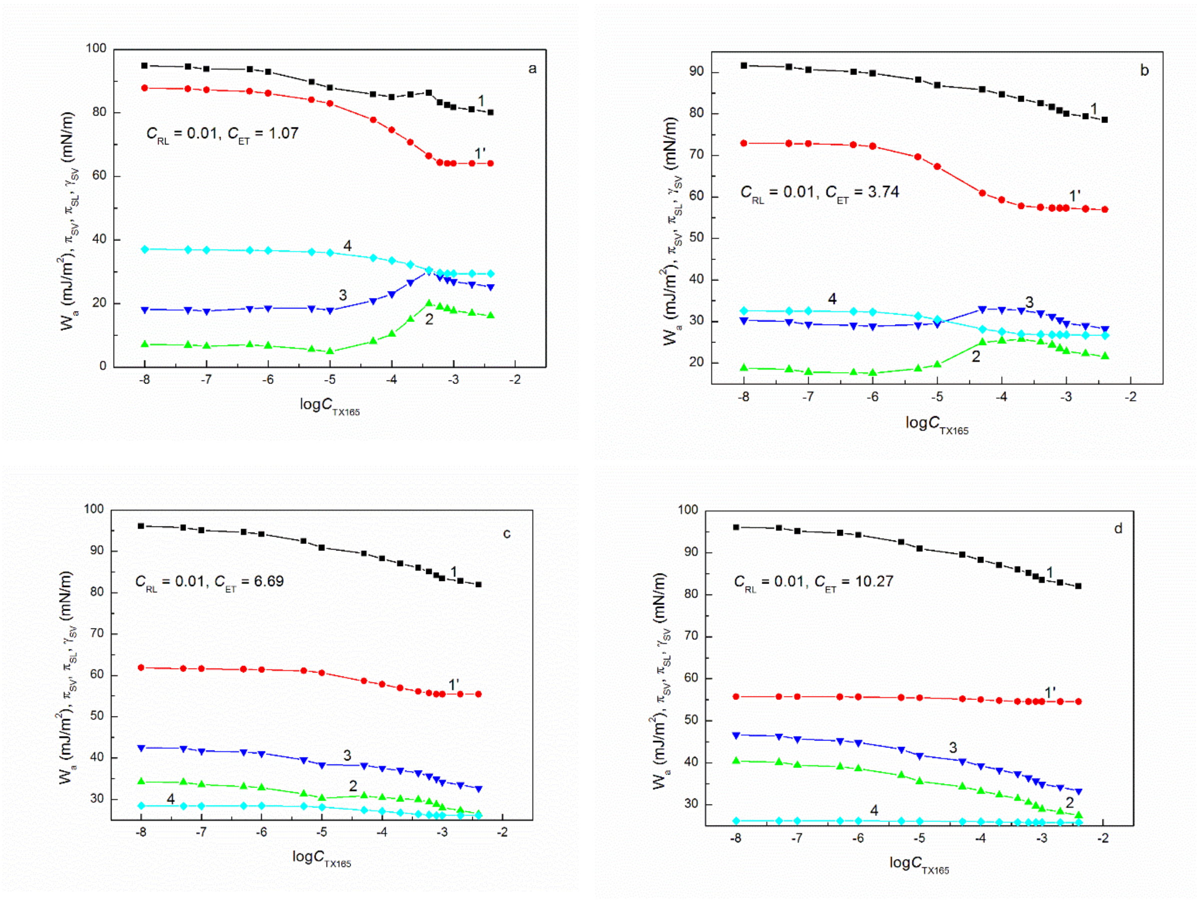

Figure S15. A plot of the ET + RL + TX165 solution work of adhesion to PMMA (W_a) calculated from the van Oss (curve 1) and the Young-Dupre equation (curve 1′), film pressure at the PMMA-air (π_{SV}) (curve 2) and PMMA-solution interface (π_{SL}) (curve 3) as well as PMMA-air interface tension (γ_{SV}) (curve 4) calculated from Equation (6) vs. the logarithm of TX165 concentration ($\log C_{TX165}$) at the constant RL concentration equal to 0.01 mg/dm^3 and the constant ET concentration equal to 1.07 mol/dm^3 (a), 3.74 mol/dm^3 (b), 6.69 mol/dm^3 (c) and 10.27 mol/dm^3 (d).

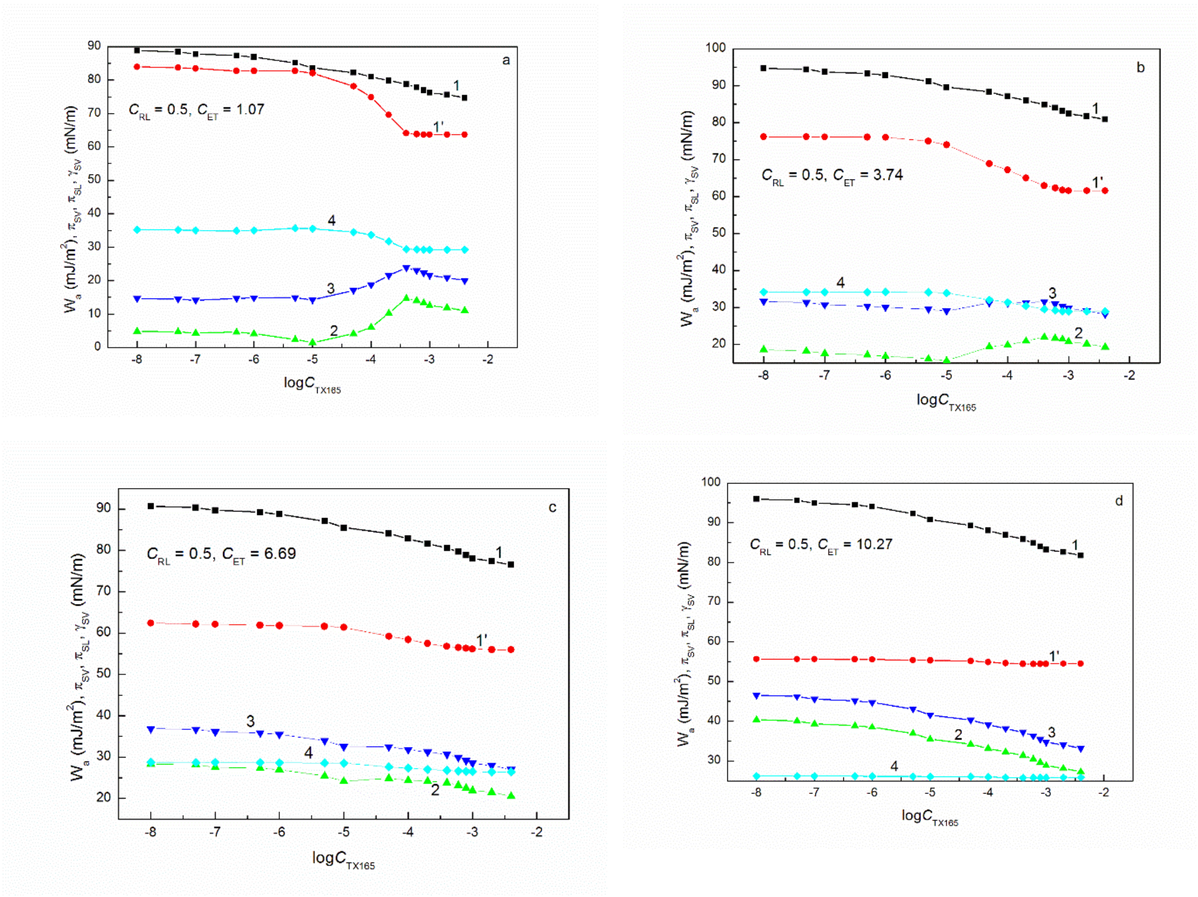

Figure S16. A plot of the ET + RL + TX165 solution work of adhesion to PMMA (W_a) calculated from the van Oss (curve 1) and the Young-Dupre equation (curve 1′), film pressure at the PMMA-air (π_{SV}) (curve 2) and PMMA-solution interface (π_{SL}) (curve 3) as well as PMMA-air interface tension (γ_{SV}) (curve 4) calculated from Equation (6) vs. the logarithm of TX165 concentration (logC_{TX165}) at the constant RL concentration equal to 0.5 mg/dm^3 and the constant ET concentration equal to 1.07 mol/dm^3 (a), 3.74 mol/dm^3 (b), 6.69 mol/dm^3 (c) and 10.27 mol/dm^3 (d).

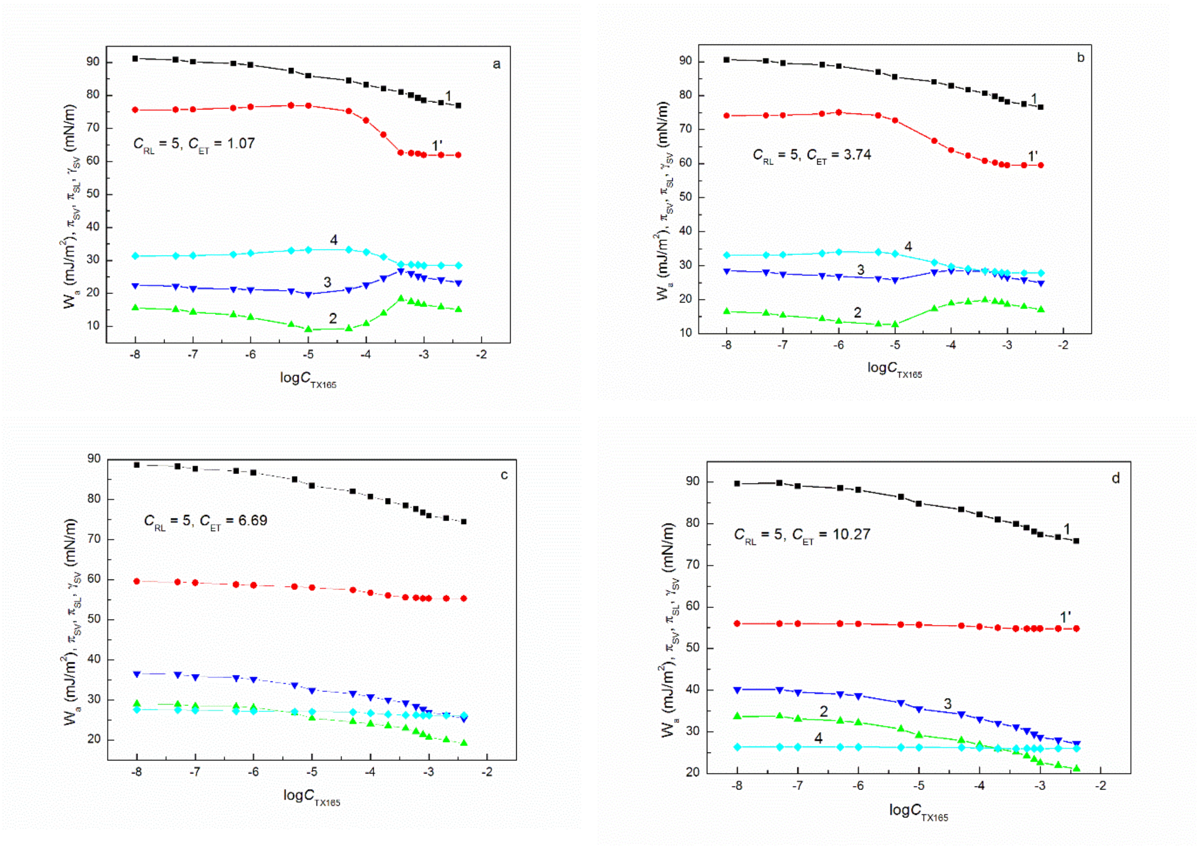

Figure S17. A plot of the ET + RL + TX165 solution work of adhesion to PMMA (W_a) calculated from the van Oss (curve 1) and the Young-Dupre equation (curve 1′), film pressure at the PMMA-air (π_{SV}) (curve 2) and PMMA-solution interface (π_{SL}) (curve 3) as well as PMMA-air interface tension (γ_{SV}) (curve 4) calculated from Equation (6) vs. the logarithm of TX165 concentration (logC_{TX165}) at the constant RL concentration equal to 5 mg/dm^3 and the constant ET concentration equal to 1.07 mol/dm^3 (a), 3.74 mol/dm^3 (b), 6.69 mol/dm^3 (c) and 10.27 mol/dm^3 (d).

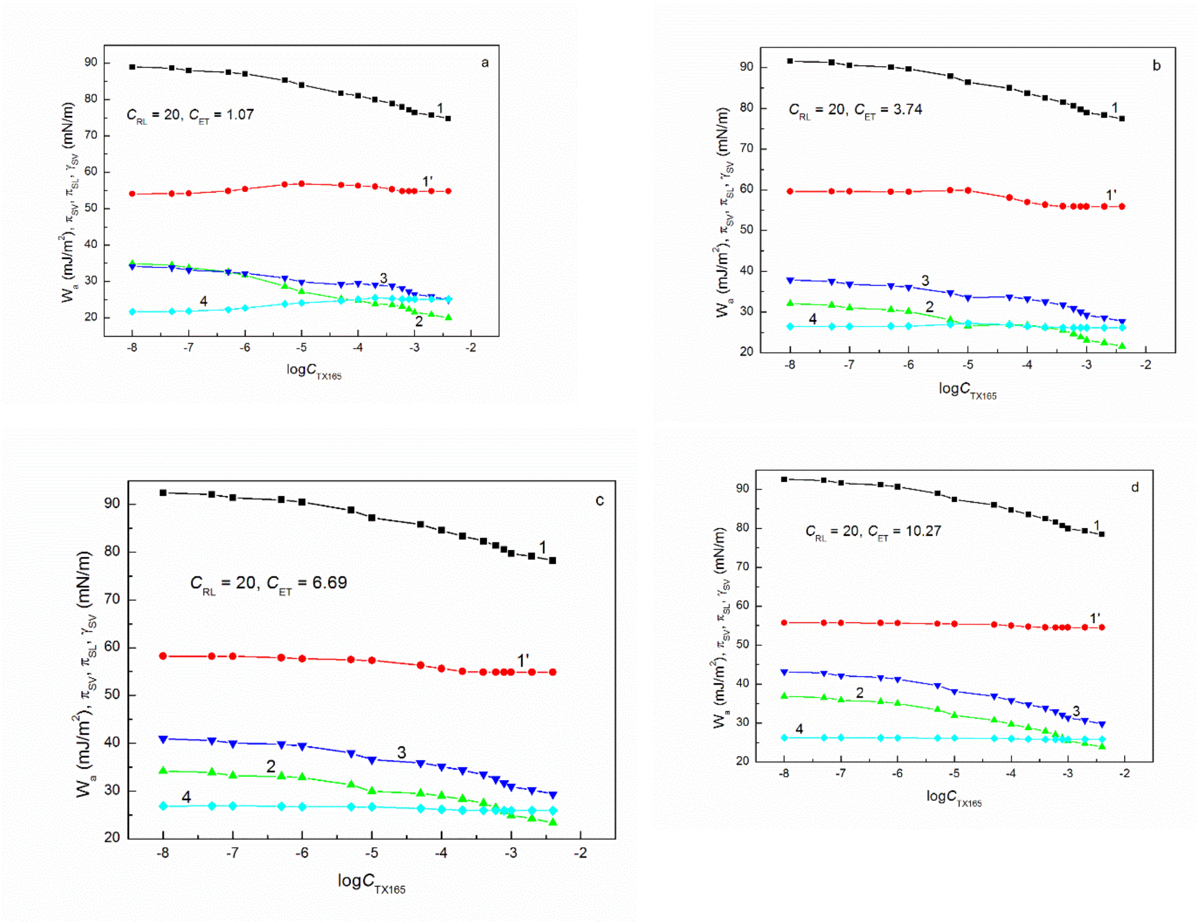

Figure S18. A plot of the ET + RL + TX165 solution work of adhesion to PMMA (W_a) calculated from the van Oss (curve 1) and the Young-Dupre equation (curve 1′), film pressure at the PMMA-air (π_{SV}) (curve 2) and PMMA-solution interface (π_{SL}) (curve 3) as well as PMMA-air interface tension (γ_{SV}) (curve 4) calculated from Equation (6) vs. the logarithm of TX165 concentration (logC_{TX165}) at the constant RL concentration equal to 20 mg/dm^3 and the constant ET concentration equal to 1.07 mol/dm^3 (a), 3.74 mol/dm^3 (b), 6.69 mol/dm^3 (c) and 10.27 mol/dm^3 (d).

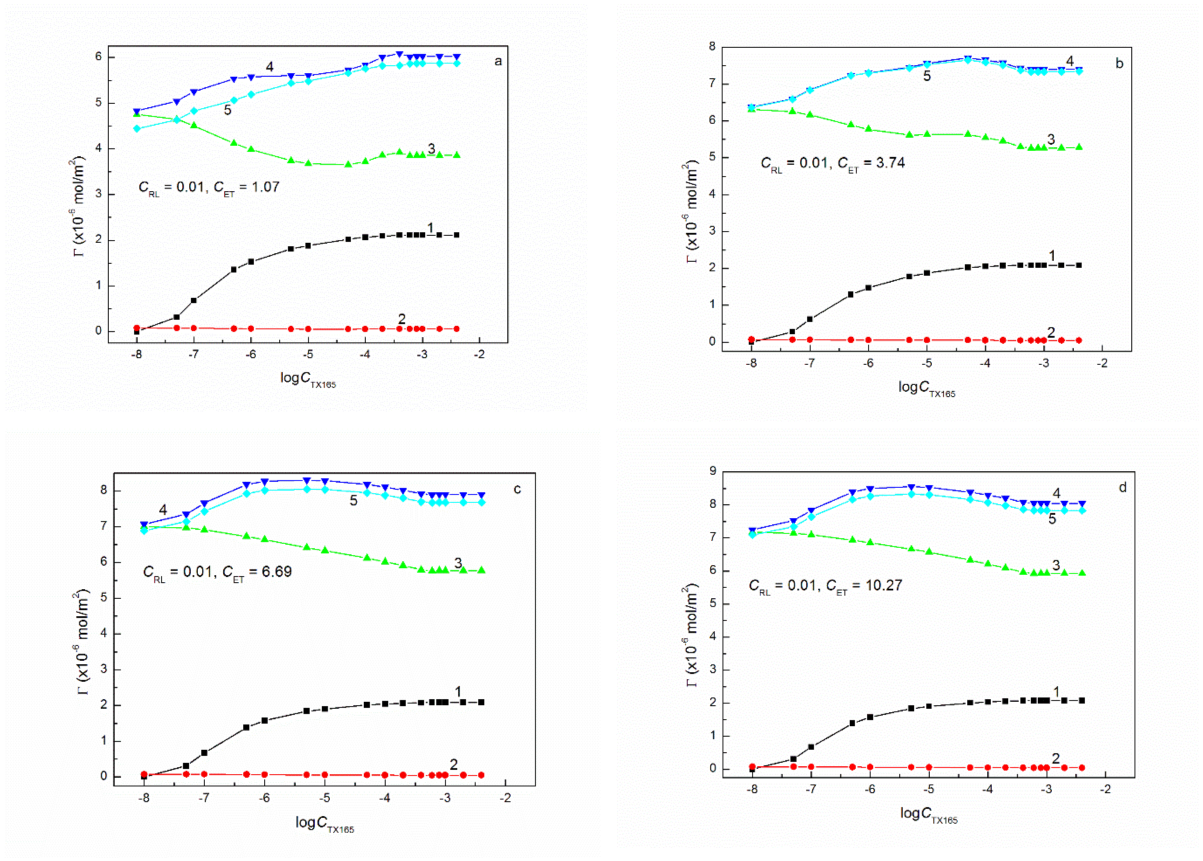

Figure S19. A plot of the surface concentration calculated from the Frumkin equation (Γ) for TX165 (curve 1), RL (curve 2), ET (curve 3) at the PTFE-solution interface as well their sum at the PTFE-solution (curve 4) and water-air interface (curve 5) vs. the logarithm of TX165 concentration (logC_{TX165}) at the constant RL concentration equal to 0.01 mg/dm^3 and the constant ET concentration equal to 1.07 mol/dm^3 (a), 3.74 mol/dm^3 (b), 6.69 mol/dm^3 (c) and 10.27 mol/dm^3 (d).

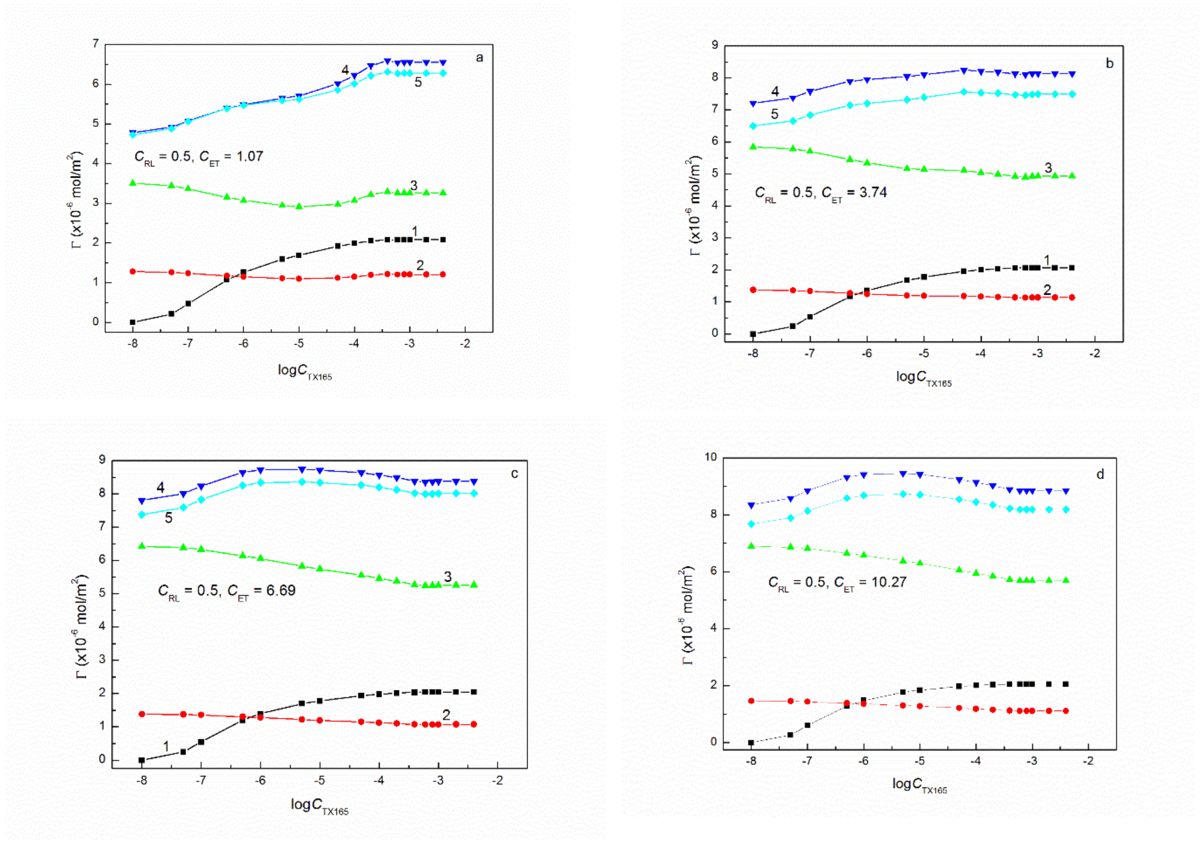

Figure S20. A plot of the surface concentration calculated from the Frumkin equation (Γ) for TX165 (curve 1), RL (curve 2), ET (curve 3) at the PTFE-solution interface (curve 3) and their sum at this interface (curve 4) as well as their sun at the water-air interface (curve 5) vs. the logarithm of TX165 concentration (logC_{TX165}) at the constant RL concentration equal to 0.5 mg/dm^3 and the constant ET concentration equal to 1.07 mol/dm^3 (a), 3.74 mol/dm^3 (b), 6.69 mol/dm^3 (c) and 10.27 mol/dm^3 (d).

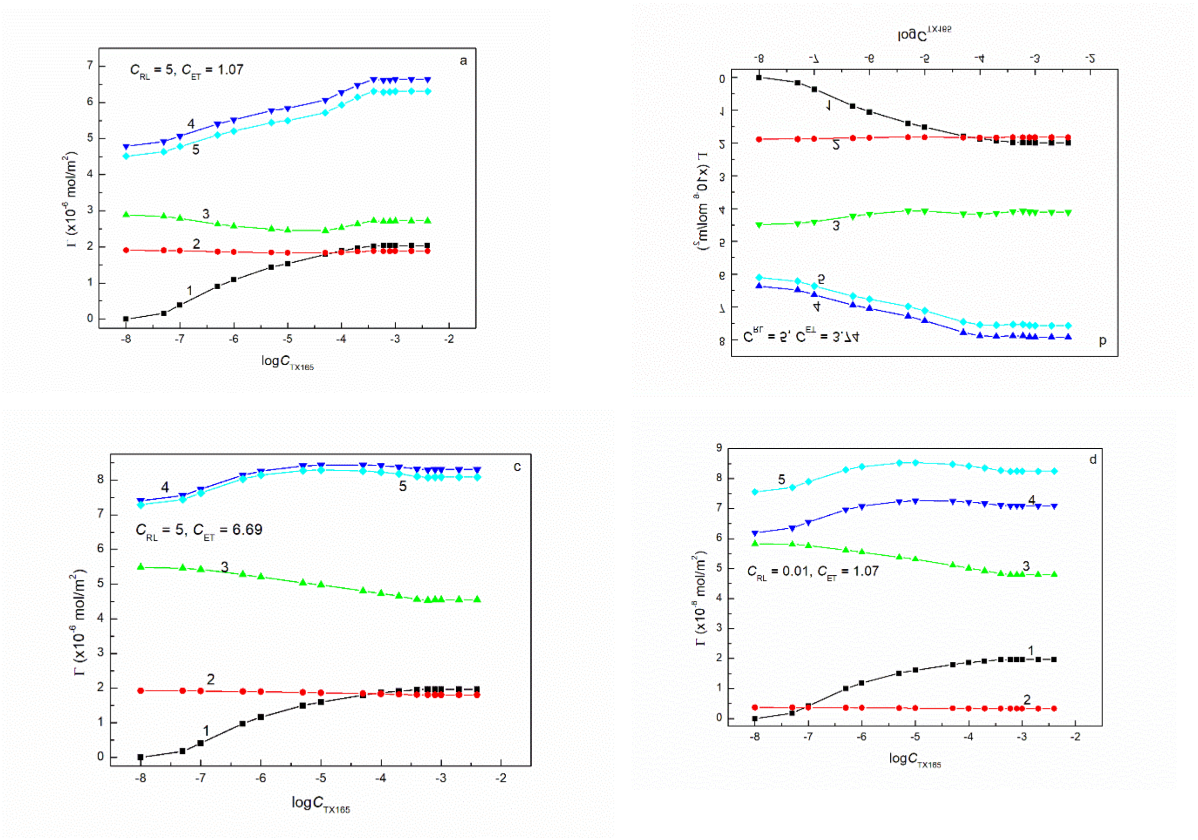

Figure S21. A plot of the surface concentration calculated from the Frumkin equation (Γ) for TX165 (curve 1), RL (curve 2), ET (curve 3) at the PTFE-solution interface (curve 3) and their sum at this interface (curve 4) as well as their sum at the water-air interface (curve 5) vs. the logarithm of TX165 concentration ($\log C_{TX165}$) at the constant RL concentration equal to 5 mg/dm^3 and the constant ET concentration equal to 1.07 mol/dm^3 (a), 3.74 mol/dm^3 (b), 6.69 mol/dm^3 (c) and 10.27 mol/dm^3 (d).

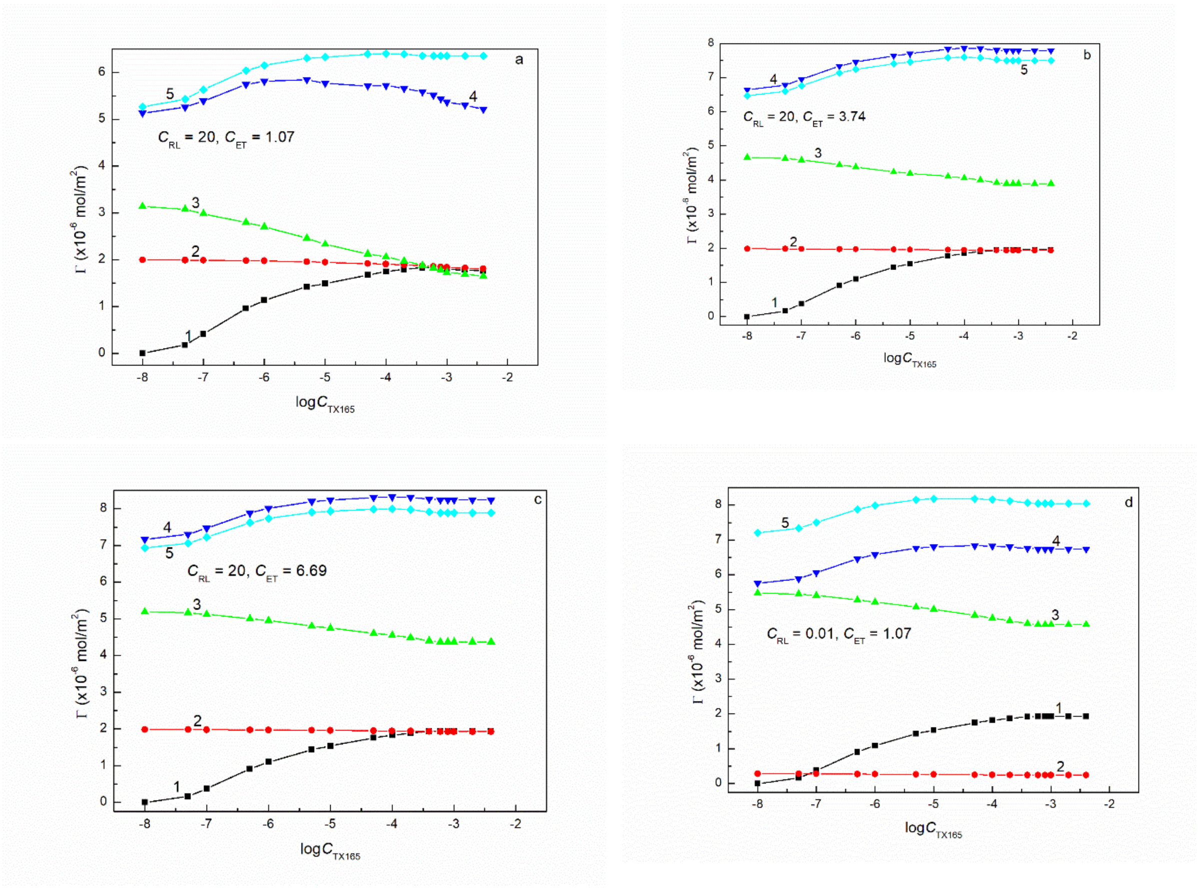

Figure S22. A plot of the surface concentration calculated from the Frumkin equation (Γ) for TX165 (curve 1), RL (curve 2), ET (curve 3) at the PTFE-solution interface (curve 3) and their sum at this interface (curve 4) as well as their sum at the water-air interface (curve 5) vs. the logarithm of TX165 concentration (logC_{TX165}) at the constant RL concentration equal to 20 mg/dm^3 and the constant ET concentration equal to 1.07 mol/dm^3 (a), 3.74 mol/dm^3 (b), 6.69 mol/dm^3 (c) and 10.27 mol/dm^3 (d).

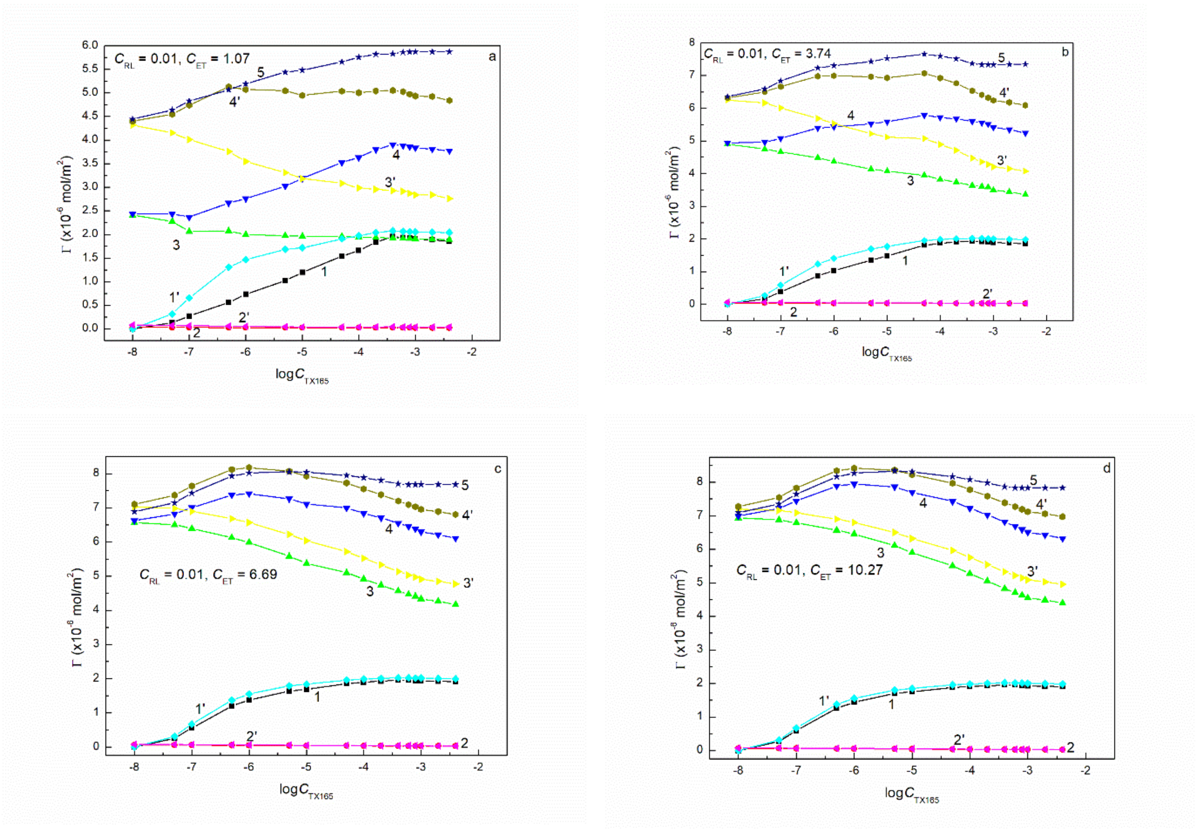

Figure S23. A plot of the surface concentration calculated from the Frumkin equation (Γ) for TX165 (curves 1 and 1′), RL (curves 2 and 2′), ET (curves 3 and 3′) and their sum (curves 4 and 4′) as well their sum at the water-air interface (curve 5) vs. the logarithm of TX165 concentration ($\log C_{\mathrm{TX165}}$) at the constant RL concentration equal to 0.01 mg/dm^3 and the constant ET concentration equal to 1.07 mol/dm^3 (a), 3.74 mol/dm^3 (b), 6.69 mol/dm^3 (c) and 10.27 mol/dm^3 (d). Curves 1 – 4 correspond to the Γ at the PMMA-air interface, curves 1′ – 4′ correspond the PMMA-solution interface.

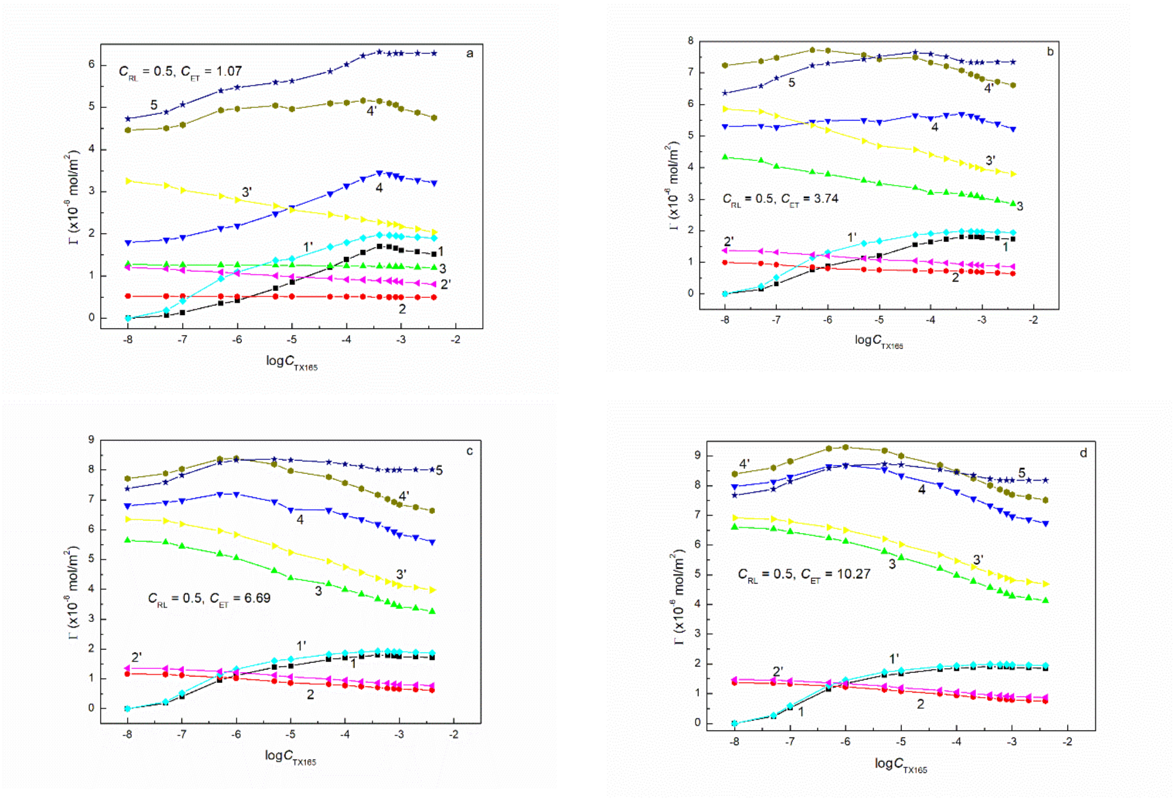

Figure S24. A plot of the surface concentration calculated from the Frumkin equation (Γ) for TX165 (curves 1 and 1′), RL (curves 2 and 2′), ET (curves 3 and 3′) and their sum (curves 4 and 4′) as well their sum at the water-air interface (curve 5) vs. the logarithm of TX165 concentration ($\log C_{\mathrm{TX165}}$) at the constant RL concentration equal to 0.5 mg/dm^3 and the constant ET concentration equal to 1.07 mol/dm^3 (a), 3.74 mol/dm^3 (b), 6.69 mol/dm^3 (c) and 10.27 mol/dm^3 (d). Curves 1 – 4 correspond to the Γ at the PMMA-air interface, curves 1′ – 4′ correspond the PMMA-solution interface.

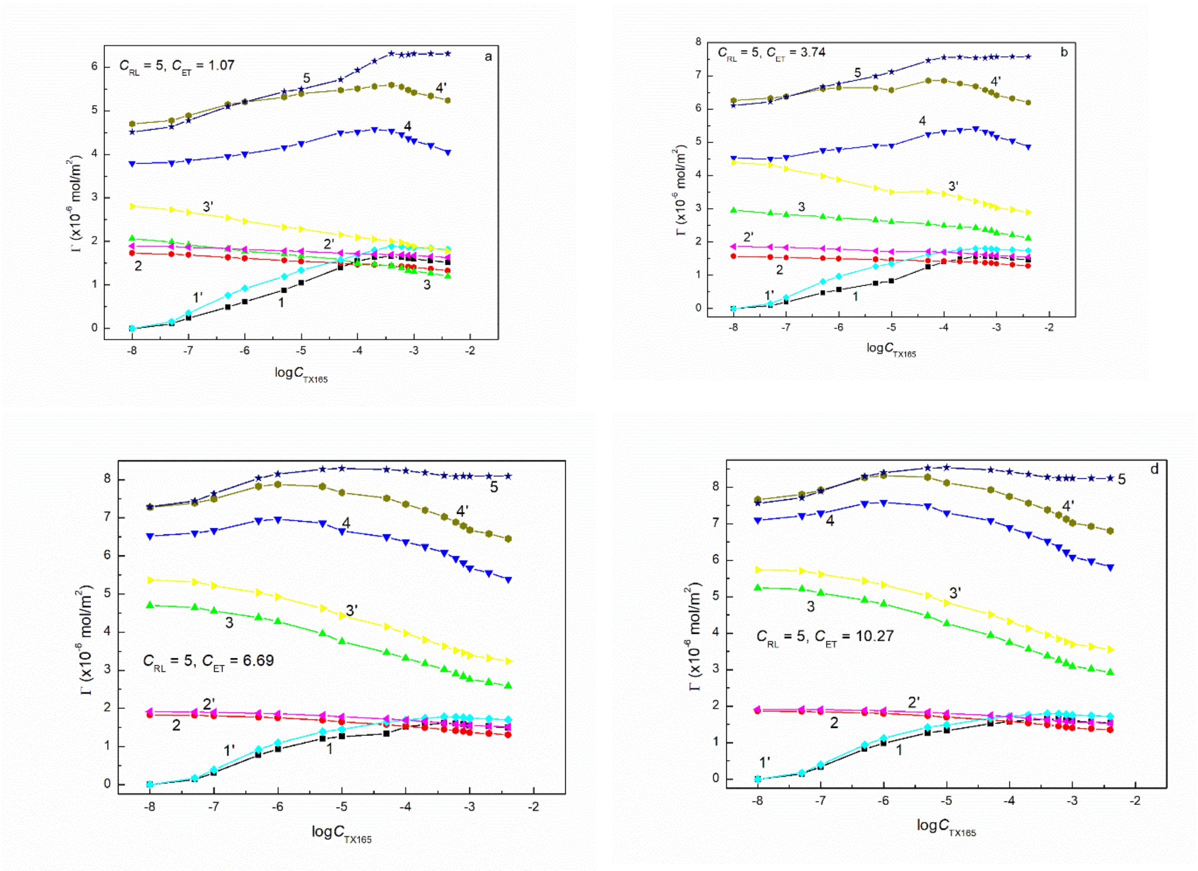

Figure S25. A plot of the surface concentration calculated from the Frumkin equation (Γ) for TX165 (curves 1 and 1′), RL (curves 2 and 2′), ET (curves 3 and 3′) and their sum (curves 4 and 4′) as well their sum at the water-air interface (curve 5) vs. the logarithm of TX165 concentration (logC_{TX165}) at the constant RL concentration equal to 5 mg/dm^3 and the constant ET concentration equal to 1.07 mol/dm^3 (a), 3.74 mol/dm^3 (b), 6.69 mol/dm^3 (c) and 10.27 mol/dm^3 (d). Curves 1 – 4 correspond to the Γ at the PMMA-air interface, curves 1′ – 4′ correspond the PMMA-solution interface.

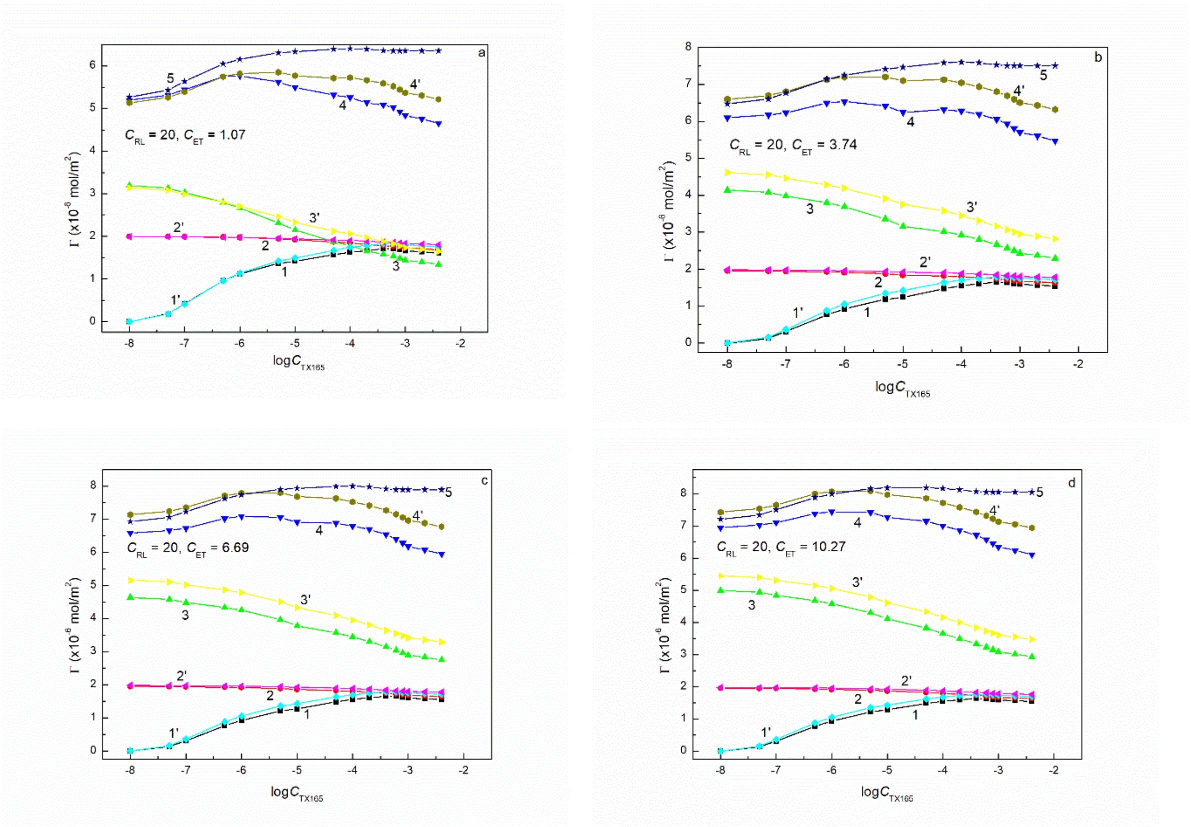

Figure S26. A plot of the surface concentration calculated from the Frumkin equation (Γ) for TX165 (curves 1 and 1′), RL (curves 2 and 2′), ET (curves 3 and 3′) and their sum (curves 4 and 4′) as well their sum at the water-air interface (curve 5) vs. the logarithm of TX165 concentration ($\log C_{TX165}$) at the constant RL concentration equal to 20 mg/dm^3 and the constant ET concentration equal to 1.07 mol/dm^3 (a), 3.74 mol/dm^3 (b), 6.69 mol/dm^3 (c) and 10.27 mol/dm^3 (d). Curves 1 – 4 correspond to the Γ at the PMMA-air interface, curves 1′ – 4′ correspond the PMMA-solution interface.

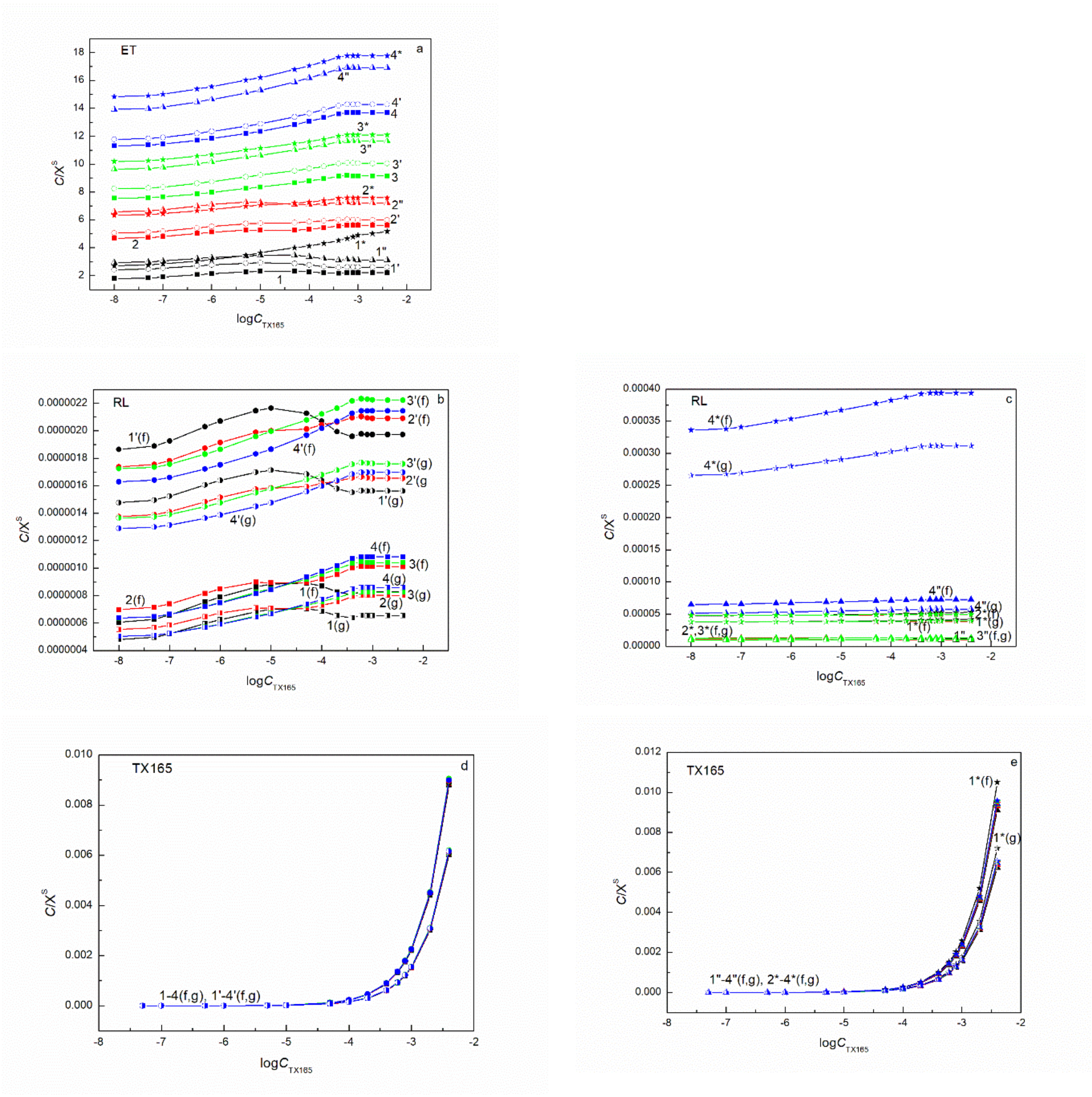

Fig. S27. A plot of the $\frac{C}{X^S}$ for ET (a) RL (b, c) and TX165 (d, e) vs. the logarithm of the TX165 concentration ($\log C_{\mathrm{TX165}}$). Curves 1 – 4, 1′ – 4′, 1″ – 4″ and 1* - 4* correspond the constant RL concentration equal to 0.01, 0.5, 5 and 20 mg/dm^3, respectively. Curves, 1, 1′, 1″ and 1* correspond to the constant ET concentration equal to 1.07 mol/dm^3, curves 2, 2′, 2″ and 2* to 3.74 mol/dm^3, curves 3, 3′, 3″ and 3* to curves 4, 4′ 4″ and 4* to 6.69 mol/dm^3. The symbol f corresponds to the RL and TX165 area equal to 69.09 and 35.7 Å^2, symbol g to 87.3 and 52.12 Å^2, respectively.

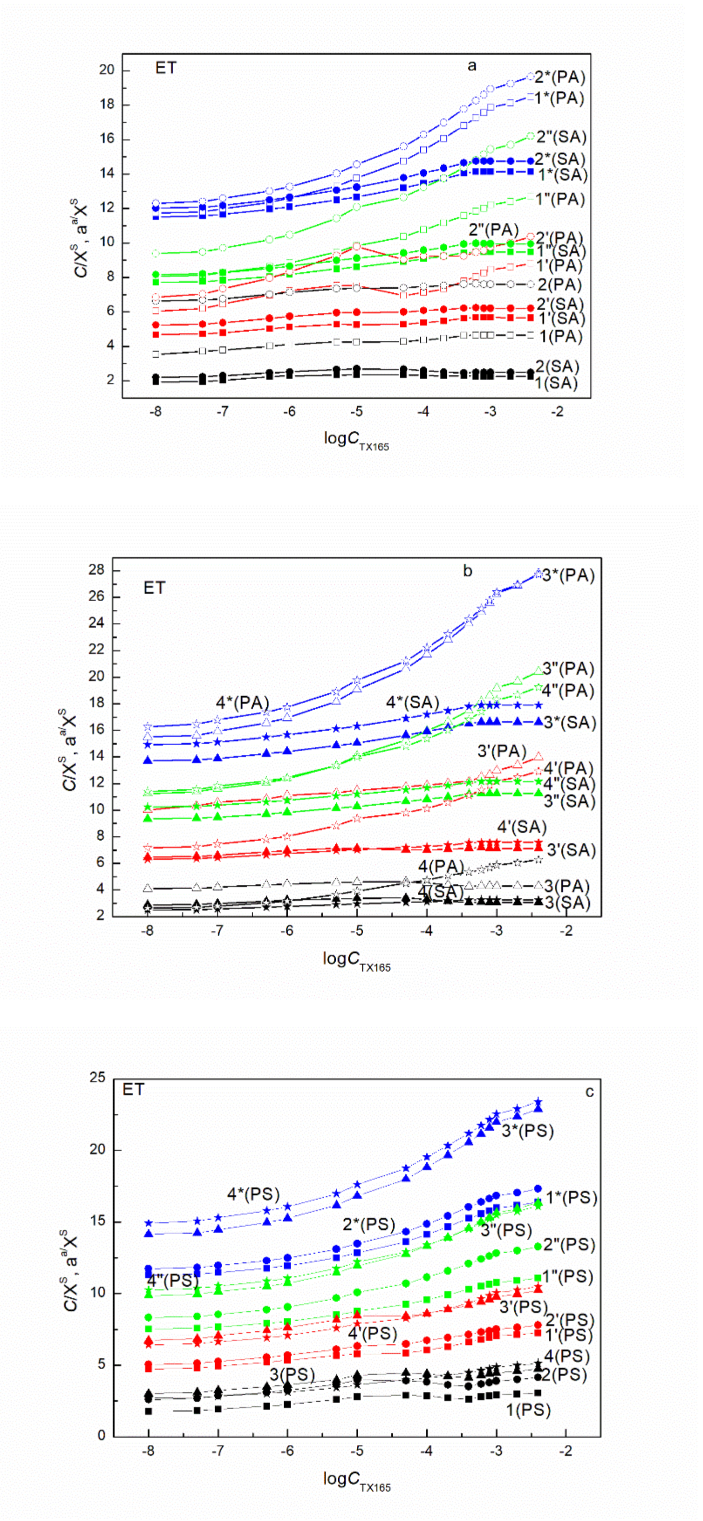

Figure S28. A plot of the $\frac{C}{X^S}$ at the solution-air interface as well as $\frac{a^a}{X^S}$ at the PMMA-air (a, b) and PMMA-solution interfaces (c) for ET (curves 1 – 4, 1′ – 4′, 1″ – 4″, 1* - 4*) vs. the logarithm of the TX165 concentration ($\log C_{\mathrm{TX165}}$). Curves 1, 1′, 1″ and 1* correspond to the constant RL concentration equal to 0.01 mg/dm^3, curves 2, 2′, 2″ and 2* to 0.5 mg/dm^3, curves 3, 3′, 3″ and 3* to 5 mg/dm^3 and curves 4, 4′, 4″ and 4* to 20 mg/dm^3. Curves 1 – 4 correspond to the ET constant concentration equal to 1.07 mol/dm^3, curves 1′ – 4′ to 3.74 mol/dm^3, curves 1″ – 4″ to 6.69 mol/dm^3 and curves 1* - 4* to10.27 mol/dm^3 (d). The symbol SA, PA and PS correspond to the solution-air, PMMA-air and PMMA-solution interfaces, respectively.

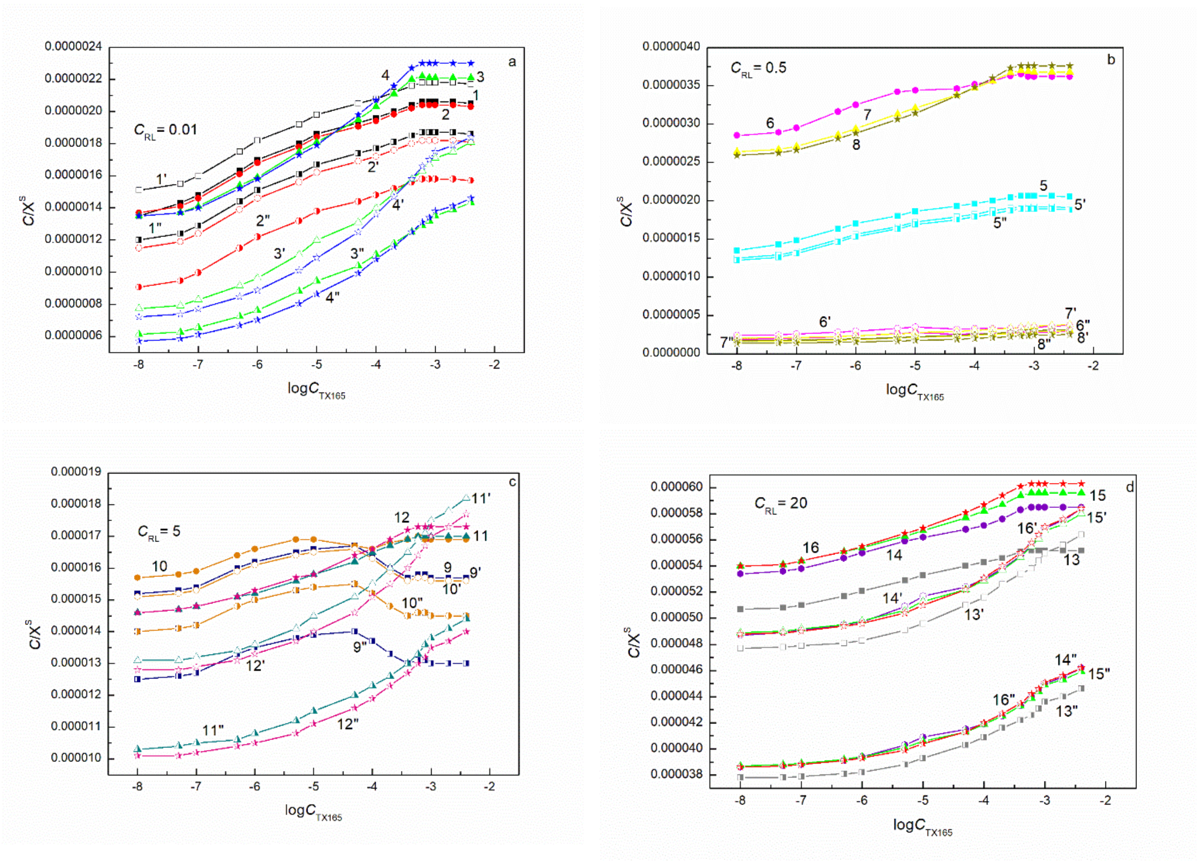

Figure S29. A plot of the $\frac{C}{X^S}$ at the solution-air interface (curves 1 – 16) and PMMA-air interface calculated for RL at its area equal to 69.09 Å^2 (curves 1′ – 16′) and 87.3 Å^2 (curves 1″ – 4″) vs. the logarithm of the TX165 concentration ($\log C_{TX165}$). Curves 1 – 4, 1′ – 4′ and 1″ – 4″ correspond to the constant RL concentration equal to 0.01 mg/dm^3 (a), curves 5 – 8, 5′ – 8′ and 5″ – 8″ to 0.5 mg/dm^3 (b), curves 9 – 12, 9′ – 12′ and 9″ – 12″ to 5 mg/dm^3 (c) and curves 13 – 16, 13′ – 16′ and 13″ – 16″ to 20 mg/dm^3 (d). Curves 1, 1′, 1″, 5, 5′, 5″, 9, 9′, 9″, 13, 13′ and 13″ correspond to the ET constant concentration equal to 1.07 mol/dm^3, 2, 2′, 2″, 6, 6′, 6″, 10, 10′, 10″, 14, 14′ and 14″ to 3.74 mol/dm^3, 3, 3′, 3″, 7, 7′, 7″, 11, 11′, 11″, 15, 15′ and 15″ to 6.69 mol/dm^3 and 4, 4′, 4″, 8, 8′, 8″, 12, 12′, 12″, 16, 16′ and 16″ to10.27 mol/dm^3.

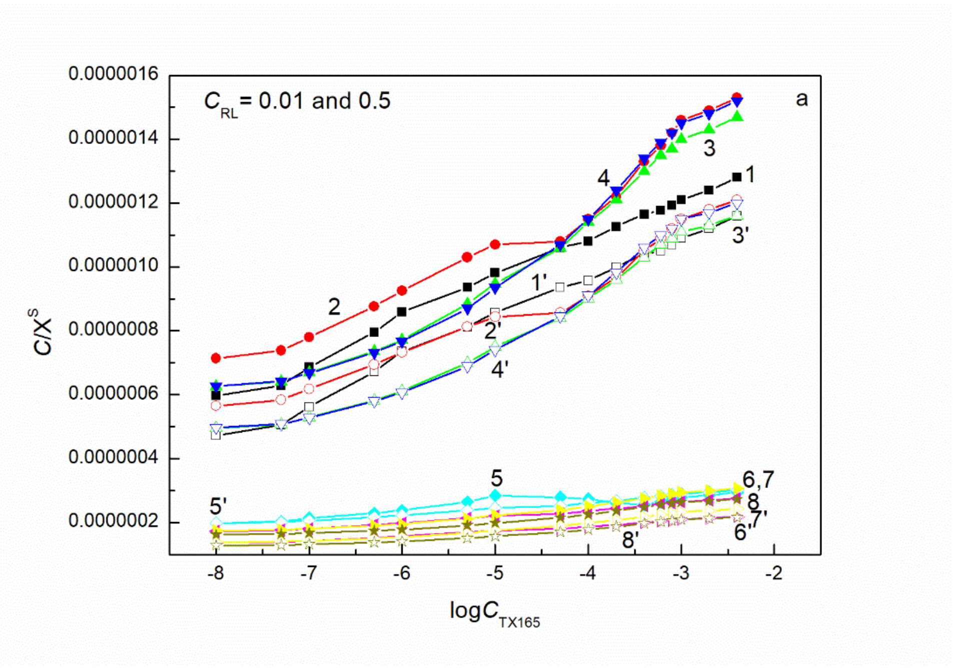

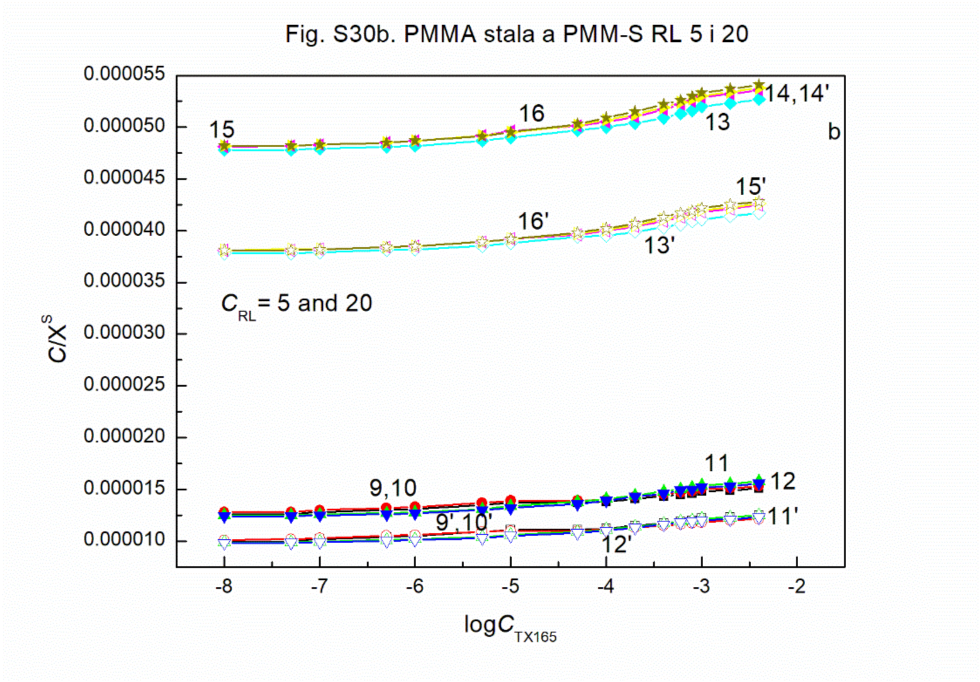

Figure S30. A plot of the $\frac{C}{X^S}$ at the PMMA-solution interface (curves 1 – 16 and 1′ – 16′) calculated for RL at its area equal to 69.09 Å^2 (curves 1 – 16) and 87.3 Å^2 (curves 1′ – 16′) vs. the logarithm of the TX165 concentration ($\log C_{TX165}$). Curves 1 – 4 and 1′ – 4′ correspond to the constant RL concentration equal to 0.01 mg/dm^3 (a), curves 5 – 8 and 5′ – 8′ to 0.5 mg/dm^3 (a), curves 9 – 12 and 9′ – 12′ to 5 mg/dm^3 (b) and curves 13 – 16 and 13′ – 16′ to 20 mg/dm^3 (d). Curves 1, 1′, 5, 5′, 9, 9′, 13 and 13′ correspond to the ET constant concentration equal to 1.07 mol/dm^3, 2, 2′, 6, 6′, 10, 10′, 14 and 14′ to 3.74 mol/dm^3, 3, 3′, 7, 7′, 11, 11′, 15 and 15′ to 6.69 mol/dm^3 and 4, 4′, 8, 8′, 12, 12′, 16 and 16′ to10.27 mol/dm^3 .

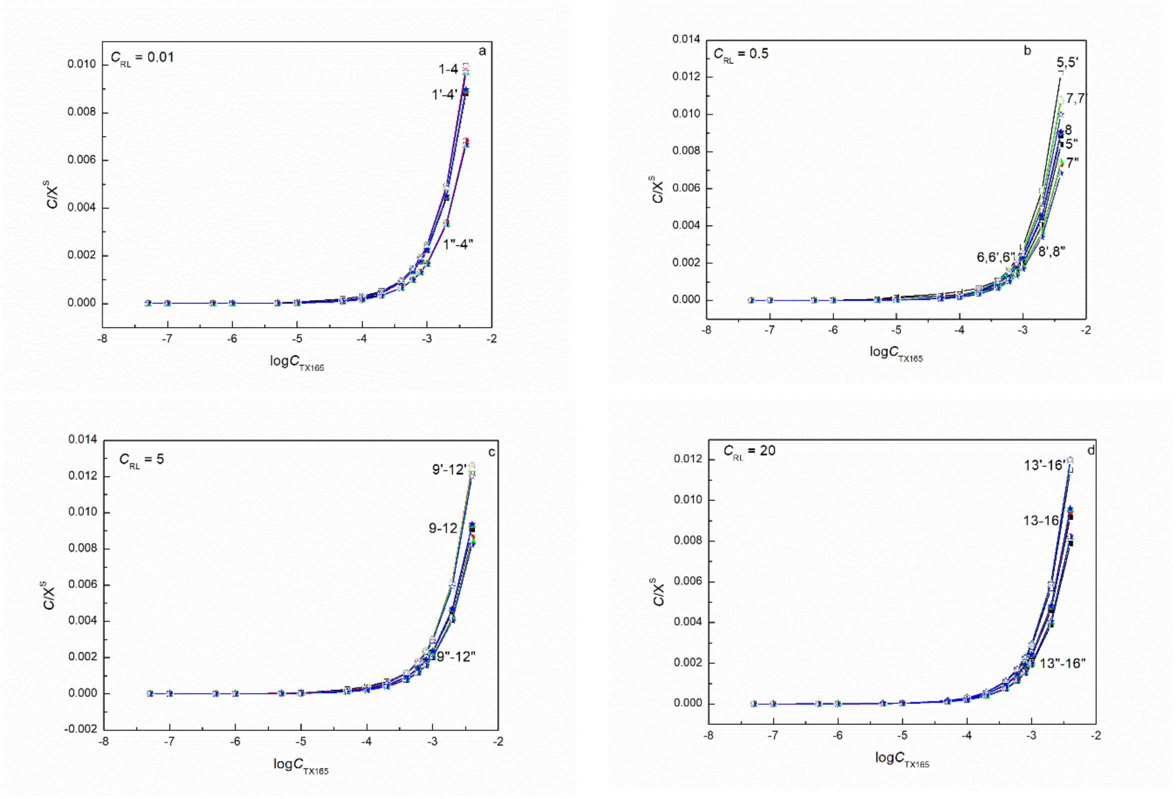

Figure S31. A plot of the $\frac{C}{X^S}$ at the solution-air interface (curves 1 – 16) and PMMA-air interface calculated for TX165 at its area equal to 35.7 Å^2 (curves 1′ – 16′) and 52.12 Å^2 (curves 1″ – 4″) vs. the logarithm of the TX165 concentration ($\log C_{TX165}$). Curves 1 – 4, 1′ – 4′ and 1″ – 4″ correspond to the constant RL concentration equal to 0.01 mg/dm^3 (a), curves 5 – 8, 5′ – 8′ and 5″ – 8″ to 0.5 mg/dm^3 (b), curves 9 – 12, 9′ – 12′ and 9″ – 12″ to 5 mg/dm^3 (c) and curves 13 – 16, 13′ – 16′ and 13″ – 16″ to 20 mg/dm^3 (d). Curves 1, 1′, 1″, 5, 5′, 5″, 9, 9′, 9″, 13, 13′ and 13″ correspond to the ET constant concentration equal to 1.07 mol/dm^3, 2, 2′, 2″, 6, 6′, 6″, 10, 10′, 10″, 14, 14′ and 14″ to 3.74 mol/dm^3, 3, 3′, 3″, 7, 7′, 7″, 11, 11′, 11″, 15, 15′ and 15″ to 6.69 mol/dm^3 and 4, 4′, 4″, 8, 8′, 8″, 12, 12′, 12″, 16, 16′ and 16″ to10.27 mol/dm^3.

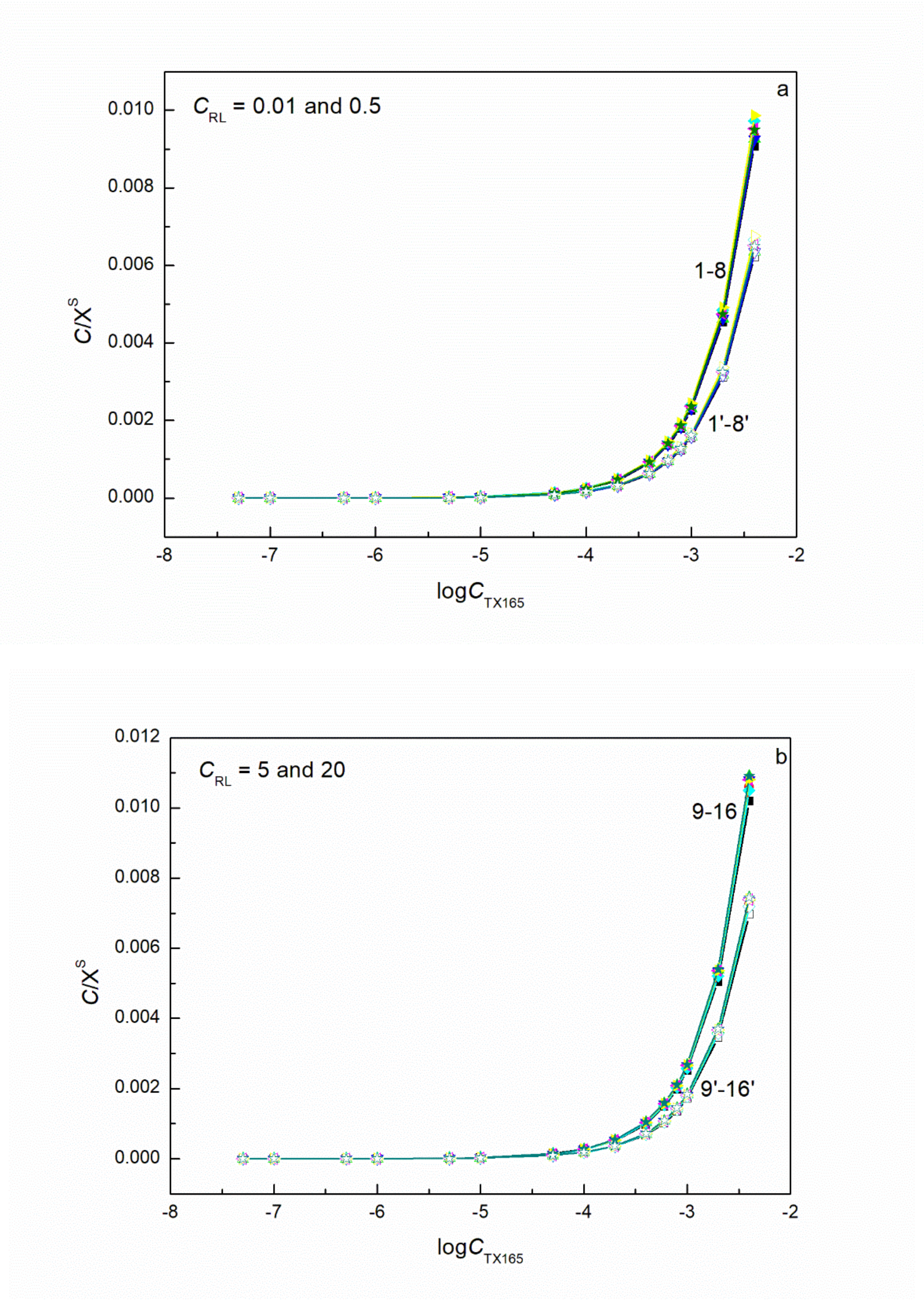

Figure S32. A plot of the $\frac{C}{X^S}$ at the PMMA-solution interface (curves 1 – 16 and 1′ – 16′) calculated for TX165 at its area equal to 35.7Å^2 (curves 1 – 16) and 52.12 Å^2 (curves 1′ – 16′) vs. the logarithm of the TX165 concentration ($\log C_{TX165}$). Curves 1 – 4 and 1′ – 4′ correspond to the constant RL concentration equal to 0.01 mg/dm^3 (a), curves 5 – 8 and 5′ – 8′ to 0.5 mg/dm^3 (a), curves 9 – 12 and 9′ – 12′ to 5 mg/dm^3 (b) and curves 13 – 16 and 13′ – 16′ to 20 mg/dm^3 (d). Curves 1, 1′, 5, 5′, 9, 9′, 13 and 13′ correspond to the ET constant concentration equal to 1.07 mol/dm^3, 2, 2′, 6, 6′, 10, 10′, 14 and 14′ to 3.74 mol/dm^3, 3, 3′, 7, 7′, 11, 11′, 15 and 15′ to 6.69 mol/dm^3 and 4, 4′, 8, 8′, 12, 12′, 16 and 16′ to10.27 mol/dm^3 .

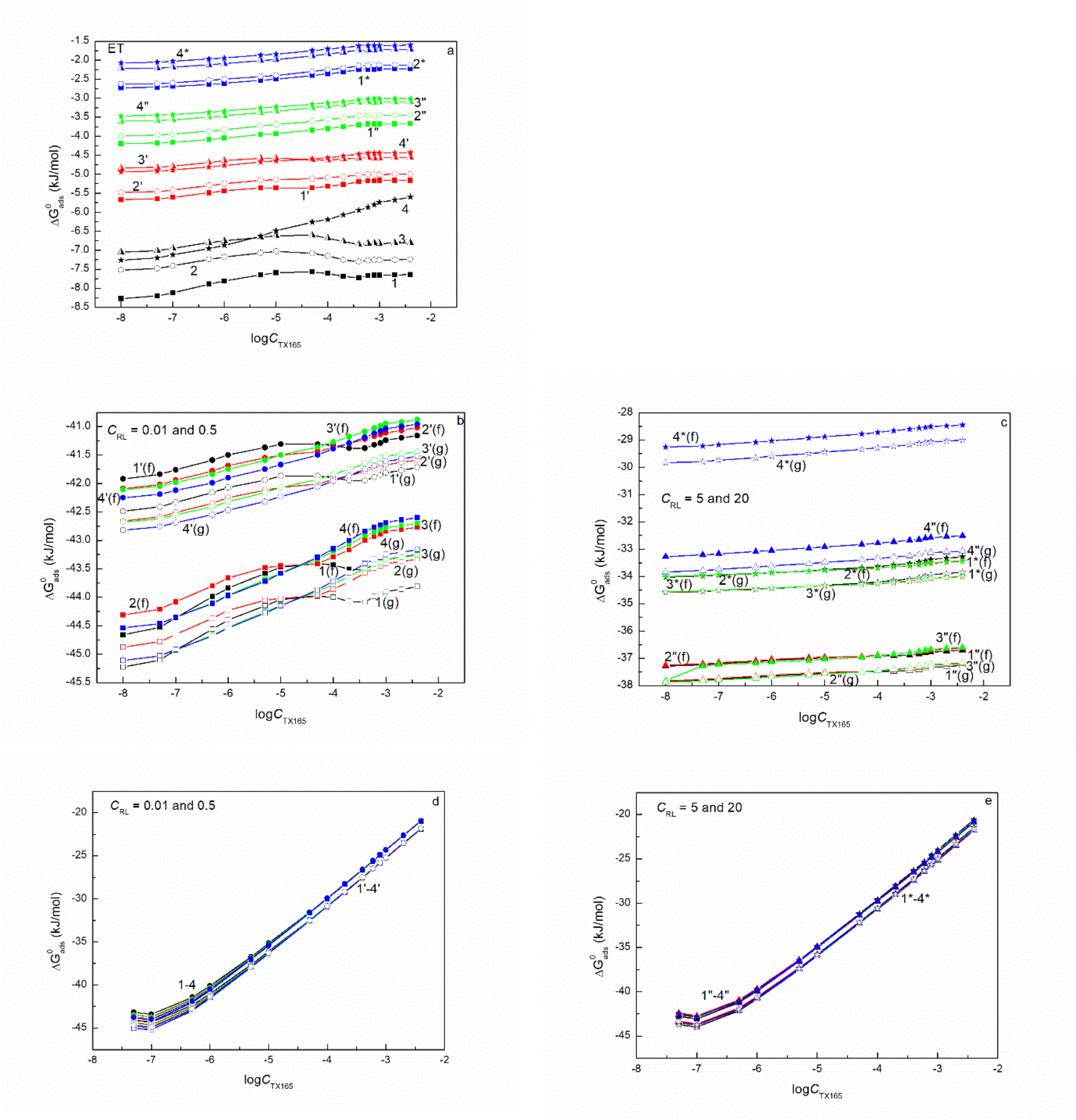

Figure S33. A plot of the standard Gibbs free energy of adsorption (ΔG^0_{ads}) at the PTFE-solution for ET (a) for RL (b, c) and TX165 (d, e) vs the logarithm of the TX165 concentration ($\log C_{TX165}$). Curves 1 – 4, 1′ – 4′, 1″ – 4″ and 1* - 4* correspond the constant RL concentration equal to 0.01, 0.5, 5 and 20 mg/dm^3, respectively. Curves, 1, 1′, 1″ and 1* correspond to the constant ET concentration equal to 1.07 mol/dm^3, curves 2, 2′, 2″ and 2* to 3.74 mol/dm^3, curves 3, 3′, 3″ and 3* to curves 4, 4′ 4″ and 4* to 6.69 mol/dm^3. The symbol f corresponds to the RL and TX165 area equal to 69.09 and 35.7 Å^2, symbol g to 87.3 and 52.12 Å^2, respectively.

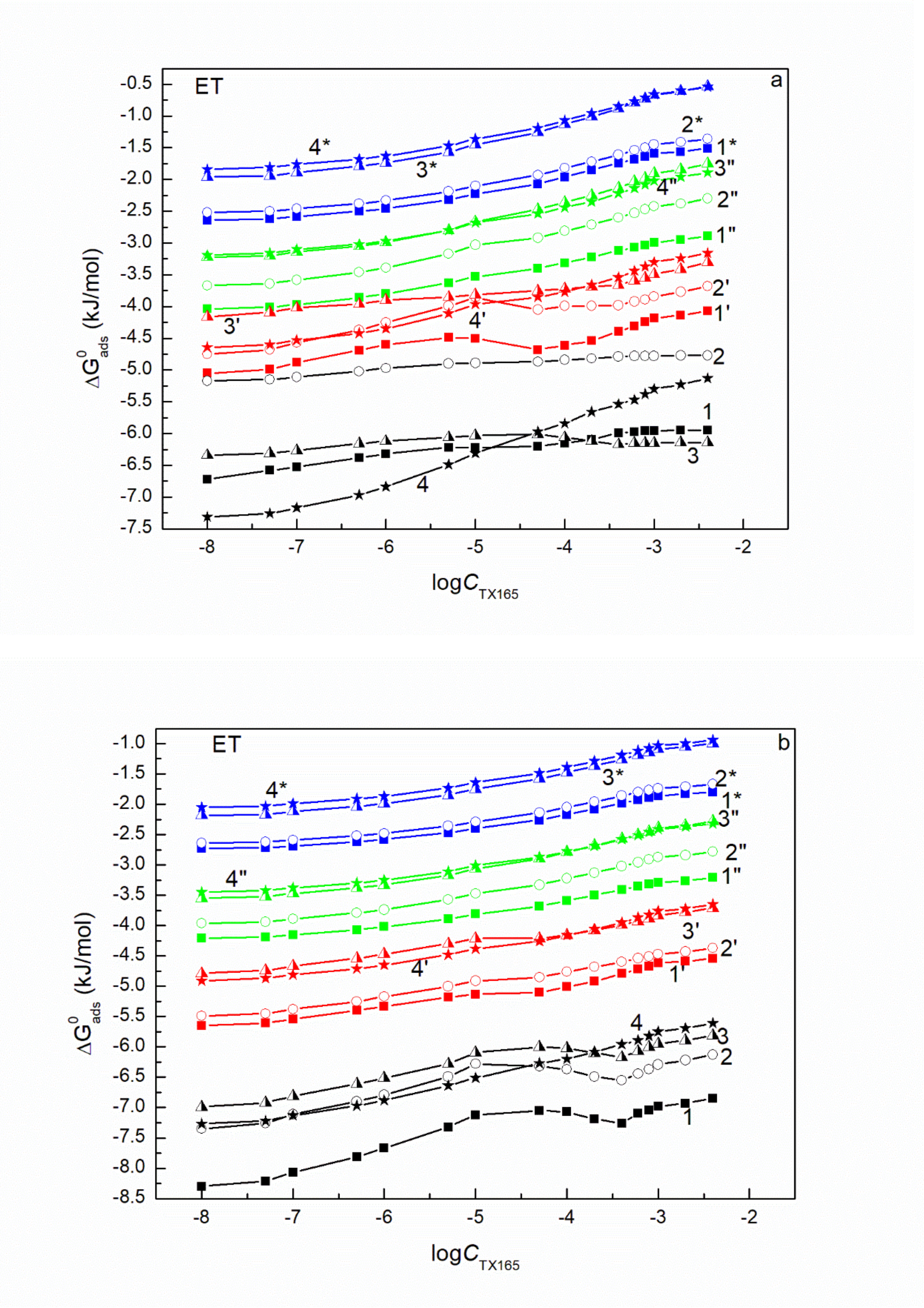

Figure S34. A plot of the standard Gibbs free energy of adsorption (ΔG^0_{ads}) for ET at the PMMA-air (a) and PMMA-solution (b) vs the logarithm the TX165 concentration ($\log C_{TX165}$). Curves 1 – 4, 1′ – 4′, 1″ – 4″ and 1* - 4* correspond the constant RL concentration equal to 0.01, 0.5, 5 and 20 mg/dm^3, respectively. Curves, 1, 1′, 1″ and 1* correspond to the constant ET concentration equal to 1.07 mol/dm^3, curves 2, 2′, 2″ and 2* to 3.74 mol/dm^3, curves 3, 3′, 3″ and 3* to curves 4, 4′ 4″ and 4* to 6.69 mol/dm^3.

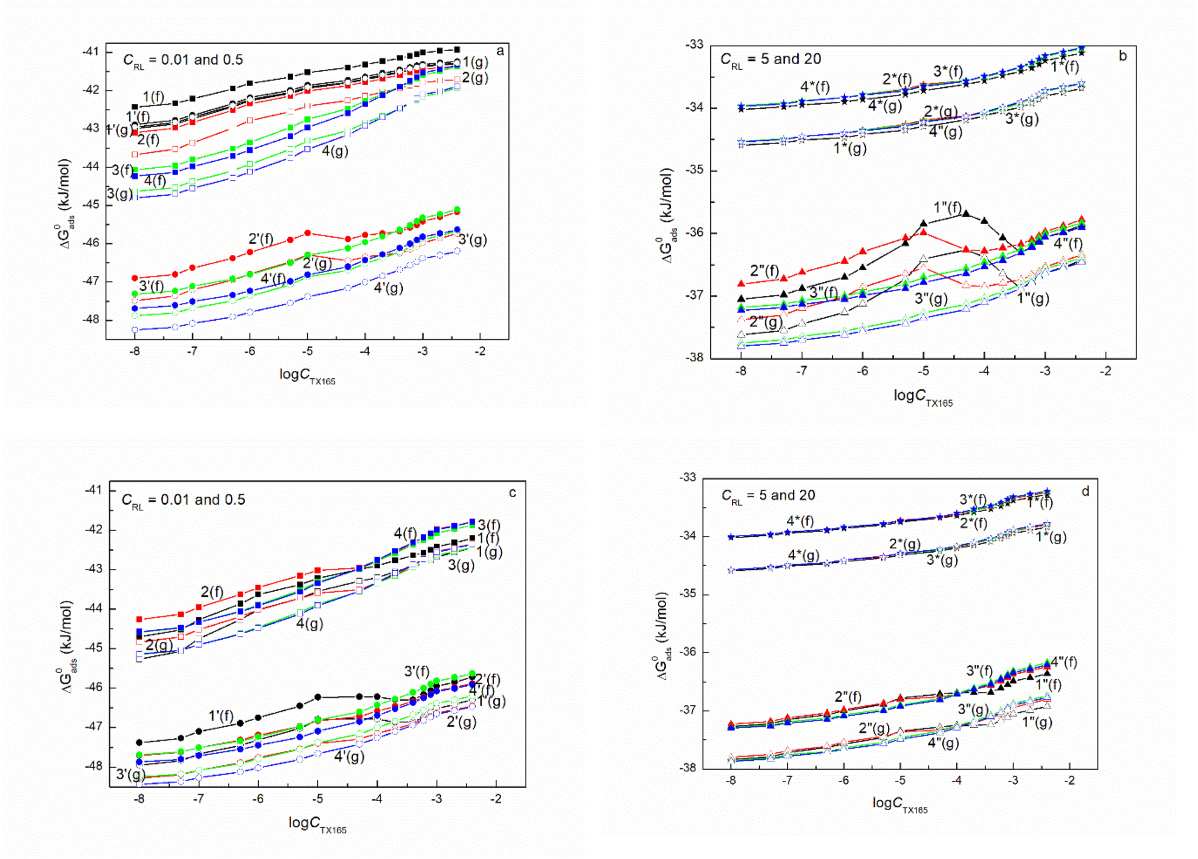

Figure S35. A plot of the standard Gibbs free energy of adsorption (ΔG^0_{ads}) for RL at the PMMA-air (a, b) and PMMA-solution (c, d) vs the logarithm of the TX165 concentration (logC_{TX165}). Curves 1 – 4, 1′ – 4′, 1″ – 4″ and 1* - 4* correspond the constant RL concentration equal to 0.01, 0.5, 5 and 20 mg/dm^3, respectively. Curves, 1, 1′, 1″ and 1* correspond to the constant ET concentration equal to 1.07 mol/dm^3, curves 2, 2′, 2″ and 2* to 3.74 mol/dm^3, curves 3, 3′, 3″ and 3* to curves 4, 4′ 4″ and 4* to 6.69 mol/dm^3. The symbol f corresponds to the RL area equal to 69.09 Å^2, symbol g to 87.3 Å^2, respectively.

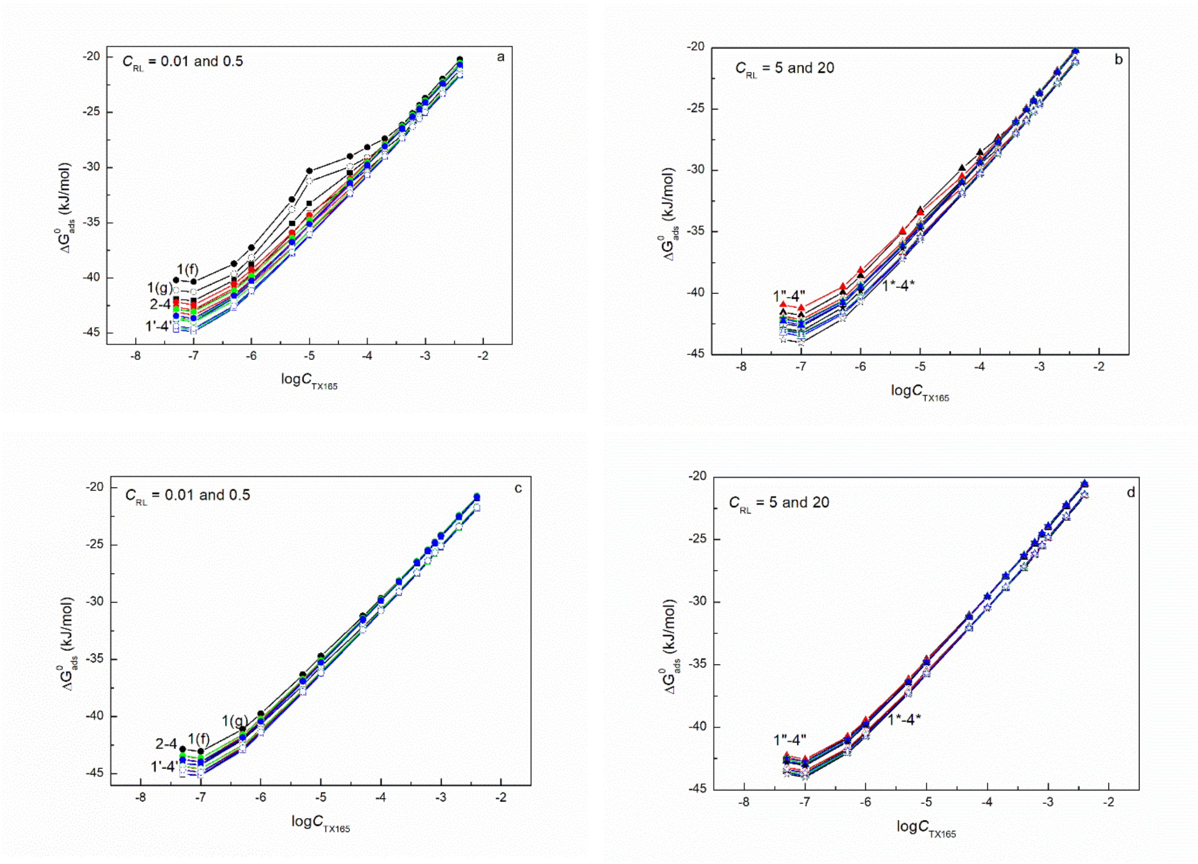

Figure S36. A plot of the standard Gibbs free energy of adsorption (ΔG^0_{ads}) for TX165 at the PMMA-air (a, b) and PMMA-solution (c, d) vs the logarithm of the TX165 concentration ($\log C_{TX165}$). Curves 1 – 4, 1′ – 4′, 1″ – 4″ and 1* - 4* correspond the constant RL concentration equal to 0.01, 0.5, 5 and 20 mg/dm^3, respectively. Curves, 1, 1′, 1″ and 1* correspond to the constant ET concentration equal to 1.07 mol/dm^3, curves 2, 2′, 2″ and 2* to 3.74 mol/dm^3, curves 3, 3′, 3″ and 3* to curves 4, 4′ 4″ and 4* to 6.69 mol/dm^3. The symbol f corresponds to the TX165 area equal to 35.7 Å^2, symbol g to 52.12 Å^2, respectively.
